# Trends in smoking prevalence in urban and rural China, 2007 to 2018: Findings from 5 consecutive nationally representative cross-sectional surveys

**DOI:** 10.1371/journal.pmed.1004064

**Published:** 2022-08-25

**Authors:** Mei Zhang, Ling Yang, Limin Wang, Yong Jiang, Zhengjing Huang, Zhenping Zhao, Xiao Zhang, Yichong Li, Shiwei Liu, Chun Li, Linhong Wang, Jing Wu, Xinhua Li, Zhengming Chen, Maigeng Zhou

**Affiliations:** 1 National Center for Chronic and Noncommunicable Disease Control and Prevention, Chinese Center for Disease Control and Prevention, Beijing, China; 2 Nuffield Department of Population Health, University of Oxford, Oxford, United Kingdom; 3 Beijing Tiantan Hospital, Capital Medical University, Beijing, China; 4 Fuwai Hospital Chinese Academy of Medical Sciences, Shenzhen, China; 5 Chinese Center for Disease Control and Prevention, Beijing, China; 6 People’s Medical Publishing House Co. LTD, Beijing, China; Fred Hutchinson Cancer Research Center, UNITED STATES

## Abstract

**Background:**

Tobacco smoking is a leading cause of premature death in China, especially among adult men. Since the implementation of the Framework Convention on Tobacco Control in 2005, nationwide tobacco control has been strengthened, but its long-term impact on smoking prevalence is unclear.

**Methods and findings:**

Five nationally representative surveys of the China Chronic Disease and Risk Factor Surveillance (CCDRFS) were conducted in 2007, 2010, 2013, 2015, and 2018. A total of 624,568 adults (278,605 men and 345,963 women) aged 18 to 69 years were randomly selected from 31 provinces (or equivalent) in China. Temporal changes in smoking prevalence and patterns (e.g., percentages of those smoking manufactured cigarettes, amount smoked, and age at smoking initiation) were analyzed, overall and by sex, urban or rural residence, year of birth, education and occupation, using linear regression methods. Among men, the standardized prevalence of current smoking decreased from 58.4% (95% confidence interval [CI]: 56.1 to 60.7) to 50.8% (95% CI: 49.1 to 52.5, *p* < 0.001) between 2007 and 2018, with annual decrease more pronounced in urban (55.7% [95% CI: 51.2 to 60.3] to 46.3% [95% CI: 43.7 to 49.0], *p* < 0.001) than rural men (59.9% [95% CI: 57.5 to 62.4] to 54.6% [95% CI: 52.6 to 56.6], *p* = 0.05) and in those born before than after 1980. Among rural men born after 1990, however, the prevalence increased from 40.2% [95% CI: 34.0 to 46.4] to 52.1% ([95% CI: 45.7 to 58.5], *p* = 0.007), with the increase taking place mainly before 2015. Among women, smoking prevalence remained extremely low at around 2% during 2007 to 2018. No significant changes of current smoking prevalence (53.9% to 50.8%, *p* = 0.22) were observed in male patients with at least 1 of major chronic diseases (e.g., hypertension, diabetes, myocardial infarction, stroke, chronic obstructive pulmonary disease (COPD)). In 2018, 25.6% of adults aged ≥18 years smoked, translating into an estimated 282 million smokers (271 million men and 11 million women) in China. Across 31 provinces, smoking prevalence varied greatly. The 3 provinces (Yunnan, Guizhou, and Hunan) with highest per capita tobacco production had highest smoking prevalence in men (68.0%, 63.4%, and 61.5%, respectively), while lowest prevalence was observed in Shanghai (34.8%). Since the children and teenage groups were not included in the surveys, we could not assess the smoking trends among youths. Furthermore, since the smoking behavior was self-reported, the smoking prevalence could be underestimated due to reporting bias.

**Conclusions:**

In this study, we observed that the smoking prevalence has decreased steadily in recent decades in China, but there were diverging trends between urban and rural areas, especially among men born after 1980. Future tobacco control strategies should target rural young men, regions with high tobacco production, and patients suffering from chronic diseases.

## Abstract 2

Mei Zhang, Ling Yang, Limin Wang, and colleagues report on trends in smoking prevalence in China based on nationally representative data from the China Chronic Disease and Risk Factor Surveillance surveys between 2007-2018.

## Author summary

**Why was this study done?:**

➢ Tobacco smoking is a leading cause of premature death in China. As the world’s largest producer and consumer of tobacco, China accounted for more than a third of the world’s tobacco consumption in 2019, almost exclusively by men. Following the ratification of the Framework Convention on Tobacco Control in 2005, nationwide tobacco control has been strengthened.
➢ Several studies had reported the prevalence of smoking in Chinese adults covering periods from 1984 to 2013. However, there were no nationwide studies on smoking prevalence trends and detailed geographic distribution beyond 2013.
➢ The trends in smoking prevalence among individuals diagnosed with major chronic diseases in the general populations are unclear.

**What did the researchers do and find?:**

➢ We used data from 5 national representative cross-sectional population-based surveys in 31 provinces (or equivalent) in mainland China during 2007 to 2018, involving 624,568 adults aged 18 to 69 years randomly sampled.
➢ The present study showed that between 2007 and 2018, the overall smoking prevalence among adults aged 18 to 69 years decreased from 30.8% to 26.7%, driven mainly by changes among men.
➢ Among men, smoking prevalence decreased from 58.4% in 2007 to 50.7% in 2018, which was more pronounced in urban (55.7% to 46.3%) than in rural areas (59.9% to 54.6%) and in those born before than after 1980. Among rural men born after 1990, however, the prevalence increased from 40.2% to 52.1%.
➢ Among women, smoking prevalence was very low and decreased slightly from 2.2% to 1.9%, although the change was not statistically significant.
➢ Compared with older generations, younger smokers tended to start smoking regularly at a much earlier age and to smoke exclusively manufactured cigarettes.
➢ Among current smokers, a relatively high proportion suffered from certain noncommunicable diseases (NCDs), with no apparent changes in prevalence between 2007 and 2018.

**What do these findings mean?:**

➢ These findings suggested that, while smoking prevalence had decreased steadily in China from 2007 to 2018, this trend was not uniform by sex, areas, birth cohorts, education attainment, and occupation. Of particular concern is the rising trend in prevalence among men born after 1990, especially in rural areas.
➢ Future smoking prevention and intervention efforts may need to be targeted more specifically on rural areas, regions with high tobacco production, and young generation.
➢ As part of secondary prevention, smokers who suffered from NCDs should be strongly encouraged to quit smoking, with adequate professional support and cessation advice along with necessary nicotine replacement therapies if needed.

## Introduction

In China, noncommunicable diseases (NCDs), mainly stroke, coronary heart disease, cancer, and chronic respiratory disease, now account for >90% of all adult deaths [1,2]. Tobacco smoking was the leading cause of disability-adjusted life years (DALYs) and the second leading cause of premature death in China in 2017 [3]. As the world’s largest producer and consumer of tobacco, China accounted for more than a third of the world’s tobacco consumption in 2019, almost exclusively by men [4]. It has been projected that the annual number of deaths attributable to tobacco would rise from approximately 1 million in 2010 to 3 million in 2050 unless there is widespread cessation among adult men [5]. In China, the WHO Framework Convention for Tobacco Control (WHO FCTC) was officially ratified in 2005, with some of its key measures (e.g., major increase in tobacco taxation and ban on smoking in public places) not formally introduced and implemented until 2015. It remains unclear, however, as to whether the nationwide tobacco control measures introduced after 2005 has had a major impact on smoking prevalence in the general population, both nationally and in specific regions or population subgroups (e.g., by education and occupation). Such evidence is critical not only for evaluating the effectiveness of existing tobacco control measures but also for informing more targeted tobacco control strategies in the future.

Since 1980, large reductions in the estimated prevalence of daily smoking were observed at the global level for both men and women [6]. Since mid-1980s, several large epidemiological studies in China, either nationally representative surveys or population-based cohort studies in particular regions, have reported on the smoking prevalence in Chinese adults. In general, these studies have demonstrated consistently the substantial sex difference in smoking prevalence, with Chinese men having the world’s highest and Chinese women having one of the lowest (at least among those who were born after the 1950s) smoking prevalence. In recent decades, while the female smoking prevalence remained extremely low and little changed, the smoking prevalence among men increased before the 1990s, decreased steadily through the early 2000s, then plateaued until the early 2010s [7–11]. However, there were no nationwide data on smoking prevalence after 2015. Moreover, few previous studies have comprehensively examined variations and long-term trends in smoking prevalence across different regions of China.

Using data from 5 large nationally representative surveys, we examined the trends of smoking prevalence in adult Chinese men and women between 2007 and 2018. Moreover, we further assessed regional variation of smoking prevalence across 31 provinces and estimated the total number of smokers in China in 2018.

## Methods

### Study design and population

The present study used data collected from 5 consecutive surveys of the China Chronic Disease and Risk Factor Surveillance (CCDRFS) conducted in year 2007, 2010, 2013, 2015, and 2018, respectively. CCDRFS was established in 2004 with the aim of providing periodic and nationally representative data for the whole of mainland China on the prevalence of smoking and other major lifestyle factors for NCDs (e.g., alcohol drinking, adiposity, physical activity, and diet) [12–16]. CCDRFS was based on the nationally representative Disease Surveillance Points (DSPs) system, which now covers 605 points (each point represents 1 rural county or urban district) from all 31 provinces, autonomous regions, or municipalities in mainland China [17,18]. The number of DSPs covered by CCDRFS has increased steadily from 79 points in Survey 2004, 161 in Surveys 2007 and 2010, to 298 in Surveys 2013, 2015, and 2018, all selected independently at random from the DSP areas [12]. In 2004, the smoking questionnaire used differed importantly from those used in subsequent surveys, so they were not included in the present report.

The target sampling population of CCDRFS was all adults aged ≥18 years (except in 2007, which covered 15 to 69 years) in DSPs covered areas who did not have any of the following situations: (a) living for less than 6 months in the current address in the preceding 12 months; (b) pregnant; and (c) with serious health condition or illness that would prevent the individual from participating, including intellectual disability or language disorder.

Each survey used a stratified, 4-stage (township/subdistrict, village/residence area, household, and individual) random sampling method to select study participants. The participants in each survey were randomly selected, with little overlap between surveys. From 2007 to 2013, only 1 family member was selected randomly among all members who met the inclusion criteria in a selected household. Since 2015, all eligible family members were invited to participate (see further details in **Appendix A in S1 Text and Fig A in S2 Text**).

Across the 5 surveys, a total of 743,391 individuals were invited and 712,969 (overall response rate: 95.9%) participated, including 51,050 (99.1%) in 2007, 98,174 (90.5%) in 2010, 189,115 (97.6%) in 2013, 189,754 (97.4%) in 2015, and 184,876 (94.9%) in 2018 (**Fig A in S2 Text**).

### Data collection

The details of the data collection procedure for CCDRFS survey have been reported previously [12–16]. Briefly, each survey included: (a) a face-to-face interviews, covering tobacco use, alcohol consumption, diet intake, physical activity, and medical history (e.g., hypertension, stroke, myocardial infarction, diabetes, chronic obstructive pulmonary disease (COPD)); (b) physical measurement of height, weight, waist circumstance, and blood pressure; and (c) laboratory tests (since 2010) of oral glucose tolerance, lipids, etc. Since the 2007 survey, all the questions related to smoking behavior remained unchanged (**Table A in S3 Text)**, while for certain other lifestyle factors (e.g., alcohol consumption, physical activity, and diet), there were small variations in the questionnaires used.

Using a standard protocol, trained health workers conducted the field survey at primary health care stations in local communities or village clinics. Data quality was maintained through standard quality control in measures. For questionnaire interviews before 2015, data collected from the paper questionnaire was double entered locally, and the database was sent to China CDC by hard copy or internet. Since 2015, a tablet-based questionnaire with built-in logic checking was used in the survey, which, with the agreement from the participant, recorded the whole process of the interview to facilitate timely quality control checks locally and centrally.

### Assessment of smoking behaviors

The information collected on tobacco use included smoking frequency, type, and numbers of cigarettes smoked daily, the age first started daily smoking and smoking cessation (**Table A in S3 Text)**. Participants were categorized into 4 smoking groups: regular, occasional, former, and never smokers. Regular smokers were defined as individuals who reported currently smoking tobacco products daily. Occasional smokers were those who currently smoke but not on a daily basis. Former smokers were those who had habitually (i.e., daily or occasional) smoked in the past but had stopped completely for at least 1 month before the survey. We then defined regular and occasional smokers as “current smokers” and current and former smokers as “ever-smokers.” The quitting ratio was defined as the proportion of former smokers divided by the number of ever smokers. Manufactured cigarettes smokers were those who smoked at least 1 manufactured cigarette per day (daily smoker) or per week (occasional smoker).

### Statistical analysis

To ensure consistency, among the 712,969 participants who completed the interviews, we excluded 74,741 who had their age outside of the 18 to 69 years range and 9,660 who had missing information on sociodemographic characteristics (e.g., sex, education attainment, occupation). We further excluded 4,000 participants with missing information on smoking behavior. After these exclusions, 624,568 (278,605 men and 345,963 women) adults aged 18 to 69 years remained for the main analyses.

All analyses accounted for small difference in sampling methods by incorporating stratification, clustering, and sampling weights. To ensure nationally representative and comparable estimates (prevalence, proportions, and means) across 5 surveys, we used standardization method based on the 2010 China census population to account for changes over time in age structure, population migration, and nonresponses [19]. This helped to harmonize the sample structure of the 5 surveys by adopting in analysis the complex sampling weights that considered nonresponse, age structure, and population migration over time (see further details in **Appendix A in S1 Text**).

The linear trends of smoking prevalence over the 5 surveys were tested using logistic regression models, both overall and by certain selected subgroups of individuals (e.g., sex, age, residence areas, education), in which both the survey years and the birth cohorts were treated as ordered categorical variables. For overall and certain key subgroup results, we further estimated the rate of annual change in smoking prevalence based on the fitted regression lines across 5 time periods. Linear regression models were used to examine the trends of mean age first started daily smoking and the number of cigarettes smoked daily by year of birth. Participants living in districts of cities are classified as urban residents, while those living in rural counties are considered as rural residents. Apart from urban and rural categories, the 31 provinces (or equivalent) in China were grouped into 7 major geographic regions to assess regional differences in smoking prevalence and patterns (**Appendix C in S1 Text**). Moreover, using 2018 China population census data and the 2018 survey data, we used the Horvitz–Thompson estimator to compute the population totals and their variance for current and former smokers for the whole of China.

A detailed analyses plan for the present study was prospectively planned and developed in 2019 (**Appendix C in S1 Text**), and no deviations from the plan took place during the execution of the analyses nor during the review process.

All statistical analyses were performed using SAS software version 9.4 (SAS Institute, Cary, North Carolina, United States) with sampling error estimated using Taylor series linearization with finite population correction. Figures were plotted using GraphPad Prism 9 software version 9.1.0. Statistical significance was determined as a 2-sided *P* value < 0.05.

### Details of ethical approval

All CCDRFS surveys were approved by the National Health Commission of China (formerly Chinese Ministry of Health). The Ethical Committee of Chinese Center for Disease Control and Prevention (China CDC) approved CCDRFS 2015, and the Ethical Committee of National Center for Chronic and Noncommunicable Disease Control and Prevention (NCNCD), China CDC, approved all other surveys except CCDRFS 2015. All participants provided written informed consent. The present study was approved by the Ethical Committee of NCNCD (No. 202019).

This study is reported as per the Strengthening the Reporting of Observational Studies in Epidemiology (STROBE) guideline (**S1 STROBE Checklist**).

## Results

Overall, the participants included in different surveys were comparable for most of the key sociodemographic characteristics (**Table 1**), except that the proportions of participants who were classified as urban residents or aged ≥50 years increased slightly across surveys, consistent with general trends of urbanization and the population ageing in China over the time.

**Table 1:** The characteristics of participants enrolled in the 5 CCDRFS surveys during 2007–2018. Values are numbers (percentages).

	Men					Women				
	2007 (*N* = 22,997)	2010 (*N* = 41,353)	2013 (*N* = 66,021)	2015 (*N* = 78,538)	2018 (*N* = 69,696)	2007 (*N* = 25,683)	2010 (*N* = 49,323)	2013 (*N* = 89,949)	2015 (*N* = 90,754)	2018 (*N* = 90,254)
**Age,** years										
18–19	402 (1.7)	1,254 (3.0)	689 (1.0)	596 (0.6)	427 (0.6)	460 (1.8)	1,278 (2.6)	568 (0.6)	504 (0.6)	281 (0.3)
20–24	1,156 (5.0)	2,940 (7.1)	2,423 (3.7)	2,616 (2.7)	1,331 (1.9)	1,295 (5.0)	2,872 (5.8)	2,431 (2.7)	2,950 (3.3)	1,657 (1.8)
25–29	1,487 (6.5)	3,017 (7.3)	3,581 (5.4)	4,572 (4.7)	2,513 (3.6)	1,728 (6.7)	3,288 (6.7)	4,220 (4.7)	6,018 (6.6)	3,716 (4.1)
30–39	5,439 (23.7)	7,964 (19.3)	9,641 (14.6)	10,456 (14.3)	7,621 (10.9)	6,312 (24.6)	9,858 (20.0)	12,867 (14.3)	12,674 (14.0)	10,861 (12.0)
40–49	5,810 (25.3)	10,875 (26.3)	17,280 (26.2)	18,938 (28.9)	14,104 (20.2)	6,525 (25.4)	13,840 (28.1)	25,974 (28.9)	22,777 (25.1)	19,308 (21.4)
50–59	5,470 (23.8)	9,286 (22.4)	17,784 (26.9)	21,203 (28.5)	20,626 (29.6)	5,764 (22.4)	11,548 (23.4)	25,657 (28.5)	24,553 (27.1)	27,628 (30.6)
60–69	3,233 (14.1)	6,035 (14.6)	14,623 (22.1)	20,157 (20.3)	23,074 (33.1)	3,599 (14.0)	6,639 (13.5)	18,232 (20.3)	21,278 (23.4)	26,803 (29.7)
**Areas**										
Urban	8,607 (37.4)	15,832 (38.3)	25,660 (38.9)	32,241 (41.8)	30,676 (44.0)	10,399 (40.5)	19,946 (40.4)	37,643 (41.8)	39,065 (43.1)	42,063 (46.6)
Rural	14,390 (62.6)	25,521 (61.7)	40,361 (61.1)	46,297 (58.2)	39,020 (56.0)	15,284 (59.5)	29,377 (59.6)	52,306 (58.2)	51,689 (56.9)	48,191 (53.4)
**Region**										
North	3,343 (14.5)	5,715 (13.8)	9,425 (14.3)	11,177 (15.3)	9,966 (14.3)	3,664 (14.3)	7,158 (14.5)	13,787 (15.3)	13,228 (14.6)	13,488 (14.9)
Northeast	2,458 (10.7)	4,649 (11.2)	6,741 (10.2)	7,401 (9.6)	6,509 (9.3)	2,725 (10.6)	5,523 (11.2)	8,675 (9.6)	8,575 (9.5)	8,689 (9.6)
East	5,709 (24.8)	10,008 (24.2)	16,746 (25.4)	19,792 (24.1)	17,462 (25.1)	6,400 (24.9)	11,790 (23.9)	21,702 (24.1)	22,558 (24.9)	22,237 (24.6)
Middle	2,975 (12.9)	5,513 (13.3)	7,933 (12.0)	9,619 (13.0)	8,692 (12.5)	3,482 (13.6)	6,728 (13.6)	11,726 (13.0)	11,430 (12.6)	11,761 (13.0)
South	2,037 (8.9)	3,590 (8.7)	6,614 (10.0)	8,081 (10.0)	6,937 (10.0)	2,234 (8.7)	4,125 (8.4)	8,992 (10.0)	9,260 (10.2)	9,330 (10.3)
Southwest	3,597 (15.6)	6,862 (16.6)	9,493 (14.4)	12,008 (15.0)	10,548 (15.1)	4,078 (15.9)	7,901 (16.0)	13,498 (15.0)	14,156 (15.6)	13,153 (14.6)
Northwest	2,878 (12.5)	5,016 (12.1)	9,069 (13.7)	10,460 (12.9)	9,582 (13.7)	3,100 (12.1)	6,098 (12.4)	11,569 (12.9)	11,547 (12.7)	11,596 (12.9)
**Education**										
Not formal/primary school	8,708 (37.9)	13,802 (33.4)	24,203 (36.7)	30,045 (38.3)	26,158 (37.6)	12,771 (49.7)	23,285 (47.2)	46,756 (52.0)	47,788 (52.7)	47,126 (52.2)
Secondary school	8,447 (36.7)	15,600 (37.7)	25,453 (38.6)	29,538 (37.6)	26,525 (38.0)	7,420 (28.9)	14,661 (29.7)	26,298 (29.2)	25,327 (27.9)	25,392 (28.1)
High school	4,016 (17.5)	7,903 (19.1)	11,147 (16.9)	12,525 (16.0)	11,543 (16.5)	3,912 (15.2)	7,567 (15.3)	11,549 (12.8)	10,556 (11.6)	10,910 (12.1)
College/university	1,826 (7.9)	4,048 (9.8)	5,218 (7.9)	6,430 (8.2)	5,470 (7.8)	1,580 (6.2)	3,810 (7.7)	5,346 (5.9)	7,083 (7.8)	6,826 (7.6)
**Occupation**										
Agriculture	11,180 (48.6)	20,910 (50.6)	34,321 (52.0)	38,042 (48.4)	33,557 (48.1)	12,508 (48.7)	22,461 (45.5)	40,599 (45.1)	39,409 (43.4)	37,252 (41.3)
Manufacture	2,343 (10.2)	2,635 (6.4)	4,857 (7.4)	4,952 (6.3)	3,776 (5.4)	1,013 (3.9)	1,085 (2.2)	2,562 (2.8)	2,127 (2.3)	1,870 (2.1)
Service provider	1,335 (5.8)	2,531 (6.1)	3,303 (5.0)	4,480 (5.7)	3,755 (5.4)	1,867 (7.3)	2,999 (6.1)	4,399 (4.9)	5,564 (6.1)	4,729 (5.2)
Managers/professionals	2,740 (11.9)	5,993 (14.5)	8,036 (12.2)	9,121 (11.6)	6,974 (10.0)	2,037 (7.9)	5,007 (10.2)	6,985 (7.8)	7,110 (7.8)	6,572 (7.3)
Others	2,354 (10.2)	3,431 (8.3)	6,846 (10.4)	9,645 (12.3)	8,482 (12.2)	2,316 (9.0)	2,442 (5.0)	5,223 (5.8)	6,660 (7.3)	6,535 (7.2)
Unemployed/students	1,593 (6.9)	3,689 (8.9)	5,294 (8.0)	7,243 (9.2)	7,561 (10.8)	3,569 (13.9)	11,519 (23.4)	23,777 (26.4)	22,630 (24.9)	23,925 (26.5)
Retired	1,452 (6.3)	2,164 (5.2)	3,364 (5.1)	5,055 (6.4)	5,591 (8.0)	2,373 (9.2)	3,810 (7.7)	6,404 (7.1)	7,254 (8.0)	9,371 (10.4)
**Self-reported NCDs**										
At least 1 of diseases	3,206 (13.9)	7,370 (17.8)	14,317 (21.7)	17,056 (21.7)	19,668 (28.2)	3,702 (14.4)	8,667 (17.6)	19,512 (21.7)	18,957 (20.9)	23,558 (26.1)
Hypertension	2,150 (9.4)	5,168 (12.5)	10,140 (15.4)	10,854 (13.8)	12,283 (17.6)	2,828 (11.0)	6,669 (13.5)	14,805 (16.5)	13,548 (14.9)	16,303 (18.1)
Diabetes	441 (1.9)	1,656 (4.0)	3,383 (5.1)	2,943 (3.7)	4,515 (6.5)	513 (2.0)	1,844 (3.7)	4,925 (5.5)	3,779 (4.2)	6,002 (6.7)
Myocardial infarction	128 (0.6)	217 (0.5)	573 (0.9)	665 (0.8)	825 (1.2)	207 (0.8)	270 (0.5)	655 (0.7)	600 (0.7)	748 (0.8)
Stroke	241 (1.1)	260 (0.6)	1,248 (1.9)	2,590 (3.3)	3,468 (5.0)	205 (0.8)	226 (0.5)	1,248 (1.4)	2,366 (2.6)	3,644 (4.0)
COPD	785 (3.4)	1,276 (3.1)	2,052 (3.1)	4,298 (5.5)	4,461 (6.4)	610 (2.4)	1,075 (2.2)	1,934 (2.2)	3,464 (3.8)	3,873 (4.3)

NCDs refer to hypertension, diabetes, myocardial infarction, stroke, and COPD.

CCDRFS, China Chronic Disease and Risk Factor Surveillance; COPD, chronic obstructive pulmonary disease; NCD, noncommunicable disease.

### Smoking prevalence

**Fig 1:**
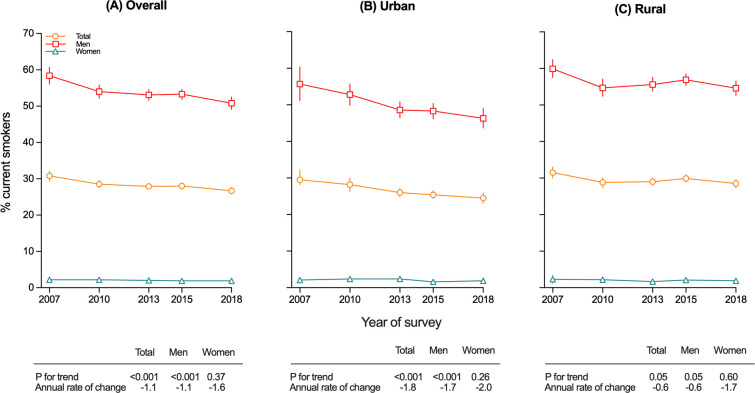
**Trends in prevalence of current smoking from 2007 to 2018 among adults aged 18–69 years, by sex for overall (A), urban (B), and rural (C).** Data from the 2007, 2010, 2013, 2015, and 2018 CCDRFS. CCDRFS, China Chronic Disease and Risk Factor Surveillance; CI: confidence interval.

Overall, among Chinese adults aged 18 to 69 years, the prevalence of current smoking decreased from 30.8% (95% confidence interval [CI]: 29.4 to 31.1) to 26.7% (95% CI: 25.8 to 27.6; *p* for trend <0.001) between 2007 and 2018 (**Fig 1 and Table 2**). Among men, the prevalence decreased on average by 1.3% per year, from 58.4% (95% CI: 56.1 to 60.7) in 2007 to 50.8% (95% CI: 49.1 to 52.5) in 2018 (*p* for trend <0.001; **Fig 1 and Table B in S3 Text**). Among women, the overall smoking prevalence was extremely low at around 2%, but decreased slightly, though not statistically significantly, between 2007 and 2018 (from 2.2% [95% CI: 1.5 to 2.9] to 1.9% [95% CI: 1.5 to 2.3]; *p* for trend = 0.37) (**Fig 1 and Table H in S3 Text**).

**Table 2:** The prevalence of current smoking among adults in China, 2007–2018.

	2007		2010		2013		2015		2018		Annual rate of change	*p* for trend
	n	% (95% CI)	n	% (95% CI)	n	% (95% CI)	n	% (95% CI)	n	% (95% CI)		
**Overall**	14,622	30.8 (29.4~32.1)	24,318	28.5 (27.5~29.6)	38,567	27.9 (27.2~28.7)	45,209	28.0 (27.2~28.8)	39,300	26.7 (25.8~27.6)	-1.1	<0.001
**Age, y**												
18–19	144	16.9 (12.0~21.8)	456	18.7 (16.2~21.3)	241	16.0 (12.9~19.1)	234	21.2 (17.1~25.2)	132	19.8 (15.0~24.7)	1.5	0.34
20–24	534	22.9 (20.0~25.9)	1,405	24.2 (22.1~26.3)	1,209	24.4 (22.3~26.5)	1,309	25.6 (23.5~27.7)	631	22.8 (20.2~25.3)	0.1	0.86
25–29	871	29.0 (26.3~31.7)	1,611	25.4 (23.5~27.3)	1,961	28.0 (26.2~29.8)	2,434	25.1 (23.1~27.1)	1,372	25.9 (24.0~27.8)	-0.9	0.09
30–39	3,553	32.7 (30.7~34.7)	4,828	29.8 (28.5~31.1)	5,433	27.7 (26.7~28.7)	5,786	27.8 (26.6~29.0)	4,070	26.5 (25.3~27.8)	-1.8	<0.001
40–49	3,976	34.9 (33.4~36.4)	6,797	31.4 (30.0~32.7)	10,599	29.9 (29.0~30.9)	11,268	29.7 (28.6~30.8)	8,163	28.1 (26.9~29.2)	-1.8	<0.001
50–59	3,666	33.8 (32.1~35.6)	5,861	31.3 (30.1~32.6)	11,037	31.1 (30.2~32.0)	13,112	31.4 (30.5~32.2)	12,366	29.7 (28.7~30.6)	-1.0	<0.001
60–69	1,878	29.6 (27.8~31.5)	3,360	28.0 (26.5~29.4)	8,087	27.4 (26.4~28.5)	11,066	27.4 (26.4~28.4)	12,566	26.9 (25.8~28.0)	-0.8	0.03
**Year of birth**												
1930–40s	2,557	30.8 (28.8~32.7)	2,927	27.9 (26.5~29.3)	4,117	26.2 (25.0~27.4)	3,354	25.0 (23.8~26.1)	943	24.5 (22.4~26.5)	-2.1	<0.001
1950s	3,798	34.7 (33.1~36.3)	5,831	31.1 (29.8~32.4)	10,820	30.4 (29.5~31.3)	12,773	29.9 (28.9~30.8)	12,684	27.8 (26.7~28.8)	-1.8	<0.01
1960s	4,239	34.3 (32.5~36.1)	6,656	31.5 (30.1~32.9)	11,135	30.6 (29.7~31.5)	13,291	31.2 (30.3~32.1)	12,438	29.0 (28.0~30.0)	-1.4	<0.001
1970s	2,931	32.0 (30.2~33.8)	5,180	30.4 (29.1~31.6)	7,342	28.6 (27.6~29.7)	8,610	28.7 (27.6~29.8)	7,514	28.1 (27.0~29.2)	-1.2	<0.001
1980s	1,097	22.6 (19.9~25.3)	3,038	24.5 (22.8~26.2)	4,104	27.0 (25.6~28.4)	5,197	26.5 (25.3~27.8)	3,961	26.4 (25.1~27.7)	1.5	0.007
1990s	NA	NA	686	20.3 (18.1~22.6)	1,049	21.9 (19.8~23.9)	1,984	24.9 (23.0~26.8)	1,760	23.4 (21.5~25.3)	2.1	0.07
**Areas**												
Urban	5,345	29.5 (26.9~32.1)	9,072	28.2 (26.3~30.0)	14,727	26.0 (24.9~27.1)	17,933	25.4 (24.4~26.5)	16,592	24.5 (23.1~25.9)	-1.8	<0.001
Rural	9,277	31.5 (30.0~33.0)	15,246	28.8 (27.4~30.1)	23,840	29.0 (28.0~30.0)	27,276	29.9 (28.9~30.9)	22,708	28.5 (27.3~29.6)	-0.6	0.05
**Region**												
North	2,118	29.8 (27.3~32.2)	3,390	28.7 (27.4~30.0)	5,673	28.3 (26.4~30.2)	6,639	29.1 (27.1~31.2)	5,852	26.9 (24.3~29.5)	-0.7	0.22
Northeast	1,745	33.8 (31.2~36.3)	2,987	32.3 (30.2~34.4)	4,357	28.4 (25.3~31.5)	4,569	27.6 (25.1~30.1)	3,998	26.2 (22.6~29.8)	-2.4	<0.001
East	3,474	29.4 (27.5~31.2)	5,483	26.6 (24.7~28.6)	9,070	26.0 (24.8~27.2)	10,694	25.3 (23.9~26.6)	8,994	23.4 (22.1~24.6)	-1.9	<0.001
Middle	1,889	32.5 (30.0~35.1)	3,270	29.2 (26.3~32.1)	4,888	30.1 (27.9~32.3)	5,543	28.9 (27.2~30.6)	5,043	29.0 (27.0~31.0)	-0.9	0.09
South	1,163	26.2 (24.3~28.1)	1,941	26.7 (24.5~28.8)	3,836	25.9 (23.9~27.9)	4,825	28.6 (26.0~31.2)	3,869	25.7 (22.9~28.5)	-0.1	0.86
Southwest	2,510	34.1 (28.1~40.0)	4,411	31.4 (28.0~34.8)	5,852	30.6 (28.6~32.6)	7,302	32.6 (30.5~34.8)	6,336	31.5 (29.1~34.0)	-0.5	0.56
Northwest	1,723	28.8 (22.4~35.1)	2,836	26.2 (21.0~31.5)	4,891	27.4 (24.6~30.2)	5,637	26.5 (24.0~29.0)	5,208	27.5 (24.7~30.3)	-0.3	0.86
**Education**												
No formal/primary school	6,005	28.9 (26.7~31.2)	8,880	25.5 (24.1~27.0)	15,157	25.2 (24.2~26.1)	18,620	26.2 (25.1~27.4)	16,151	25.5 (24.1~27.0)	-0.9	0.05
Secondary school	5,455	34.7 (33.0~36.5)	9,317	32.2 (30.6~33.7)	15182	32.1 (30.9~33.2)	17,268	32.7 (31.7~33.7)	15,072	31.3 (30.2~32.5)	-0.7	0.02
High school	2,354	30.0 (28.2~31.8)	4,333	30.0 (28.5~31.6)	5990	28.4 (26.9~29.9)	6,793	30.0 (28.6~31.4)	6,014	29.7 (28.0~31.3)	-0.1	0.79
	College/University	808	22.6 (19.9~25.3)	1,788	22.8 (21.0~24.5)	2238	21.1 (19.3~22.9)	2,528	18.9 (17.6~20.3)	2,063	16.8 (15.3~18.4)	-2.8	<0.001
**Occupation**												
Agriculture	7,529	31.9 (29.9~33.8)	12,761	31.1 (29.4~32.8)	20753	31.0 (29.9~32.1)	22,843	31.4 (30.3~32.5)	19,796	31.0 (29.7~32.2)	-0.2	0.64
Manufacture	1,575	48.3 (44.5~52.1)	1,619	42.5 (37.0~48.0)	2897	42.7 (40.2~45.3)	2,939	43.5 (40.9~46.2)	2,117	41.9 (39.4~44.5)	-1.0	0.04
Service provider	809	27.0 (24.1~30.0)	1,489	28.9 (26.5~31.3)	1837	27.7 (25.3~30.0)	2,491	27.1 (25.5~28.6)	1,990	25.7 (23.8~27.5)	-0.6	0.19
Managers/professionals	1,513	33.0 (29.8~36.2)	3,096	29.2 (27.2~31.1)	3948	27.4 (25.4~29.4)	4,542	27.1 (25.0~29.2)	3,236	23.1 (21.6~24.7)	-2.9	<0.001
Others	1,532	34.8 (32.0~37.7)	2,125	37.4 (35.2~39.7)	4081	36.4 (34.3~38.5)	5,725	37.0 (35.2~38.9)	4,844	33.6 (31.3~35.8)	-0.3	0.45
Unemployed/students	911	16.1 (14.2~17.9)	2,173	14.6 (13.3~16.0)	3422	13.0 (11.8~14.2)	4,266	14.2 (13.1~15.2)	4,601	16.4 (14.7~18.1)	0.0	0.58
Retired	753	21.5 (19.0~23.9)	1,055	19.5 (17.4~21.7)	1629	19.1 (17.6~20.7)	2,403	19.7 (18.6~20.9)	2,716	19.7 (17.7~21.6)	-0.6	0.34

CI, confidence interval; NA, data not available. Percentages and 95% CI are weighted.

Among men, the decreasing trend in smoking prevalence was more pronounced in urban (from 55.7% [95% CI: 51.2 to 60.3] to 46.3% [95% CI: 43.7 to 49.0], *p* for trend <0.001) than in rural areas (from 59.9% [95% CI: 57.5 to 62.4] to 54.6% [95% CI: 52.6 to 56.6]; *p* for trend = 0.50; *p* for difference = 0.03; **Fig 1B and 1C and Table B in S3 Text**). Within rural areas, the decreasing trend appeared less clear in those classified as poor rural regions (**Table F in S3 Text**). The downward trend was evident across all educational groups before 2010, but after 2010, it appeared much less obvious in those without college or university education (**Table B in S3 Text**). Similarly, across different occupations, the trend was not uniform, with the most striking decreasing trend observed among service providers (59.2% [95% CI: 54.0 to 64.4] to 46.4% [95% CI: 42.7 to 50.1]; 2.0% annual rate of decrease; *p* for trend <0.001; *p* for difference = 0.003) and professional occupations (53.0% [95% CI: 49.0 to 57.1] to 42.6% [95% CI: 39.9 to 45.3]; 1.8% annual rate of decrease; *p* for trend <0.001; *p* for deference < 0.01). Among men who were unemployed or students, there was a significant increasing trend (37.7% to 47.7%; 1.9% annual rate of increase; *p* for trend 0.006; **Table B in S3 Text**).

**Fig 2:**
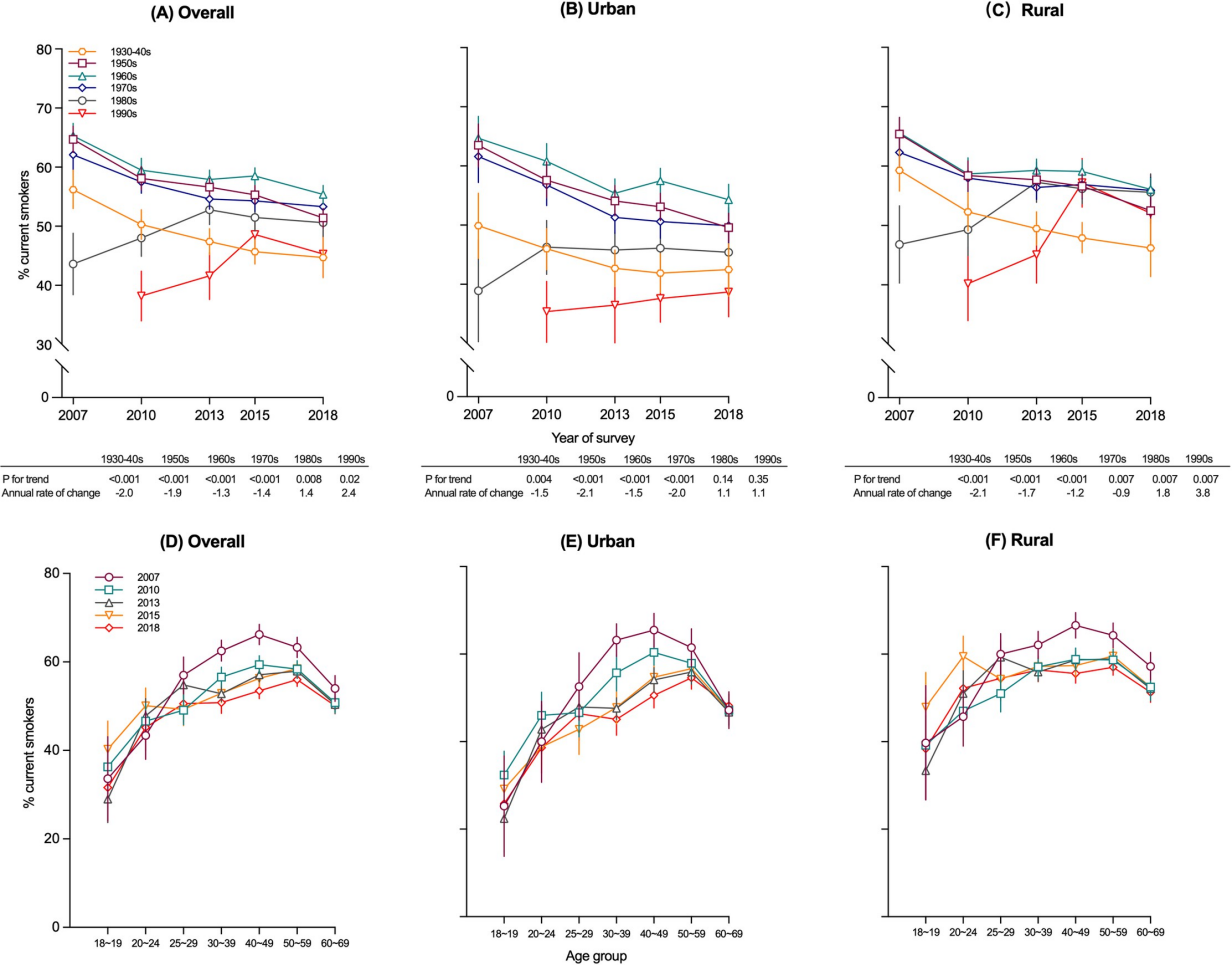
**Trends in prevalence of current smoking from 2007 to 2018 among men aged 18–69 years, by year of birth for overall (A), urban (B) and rural (C), or age group for overall (D), urban (E) and rural (F).** Data from the 2007, 2010, 2013, 2015, and 2018 CCDRFS. CCDRFS, China Chronic Disease and Risk Factor Surveillance; CI, confidence interval.

Across all surveys, men born in the 1960s consistently had the highest smoking prevalence compared with men born in other decades. In contrasting to a decreasing trend among men born before 1980, a significant increasing trend in the prevalence of current smoking among men born after 1980 was observed (*p* for trend <0.001 for 1980s and *p* for difference = 0.004), with a considerable increase in rural men born in the 1990s (from 40.2% [95% CI: 34.0 to 46.4] in 2010 to 52.1% [95% CI: 45.7 to 58.5] in 2018; *p* for trend = 0.007), especially before 2015 (**Fig 2 and Tables B and G in S3 Text**).

Among male smokers, the quitting ratio increased slightly from 11.3% (95% CI: 10.1 to 12.6) in 2007 to 13.4% (95% CI: 12.6 to 14.1) in 2018 (*p* for trend = 0.01; **Table L in S3 Text**). Although the quit ratio slightly increased in those aged 50 to 59 years (*p* for trend = 0.009) and 40 to 49 years (*p* for trend = 0.03), overall there was no statistically significant difference between age groups. No significant changes of current smoking prevalence (53.9% [95% CI: 51.3 to 56.6] to 50.8% [95% CI: 49.0 to 52.2], *p* for trend = 0.22) were observed in male patients with at least 1 of major chronic diseases (e.g., hypertension, diabetes, myocardial infarction, stroke, COPD). A decrease of current smoking prevalence was observed among urban men with COPD (66.8% to 48.3%, *p* for trend <0.001), but no significant changes were observed in those who had other prior medical history of chronic diseases (all *p* values >0.05; **Tables M and N in S3 Text**).

### Smoking patterns

**Fig 3:**
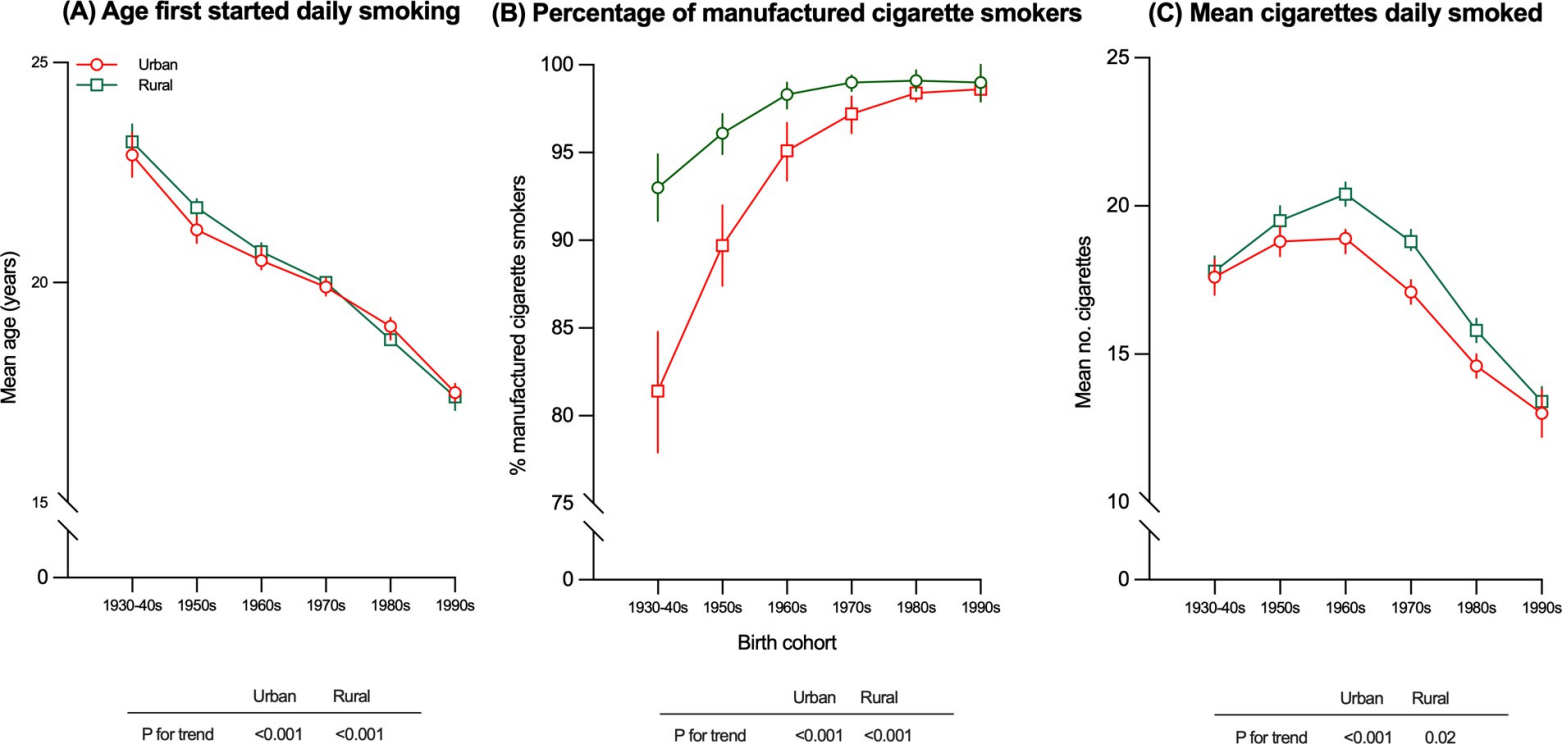
**Age first stared daily smoking (A), percentage of manufactured cigarette smokers (B), and mean cigarettes daily smoked (C) among male regular smokers aged 18–69 years, by year of birth.** Data from the 2007, 2010, 2013, 2015, and 2018 CCDRFS. CCDRFS, China Chronic Disease and Risk Factor Surveillance, CI, confidence interval.

Among male regular smokers, the reported mean age first started daily smoking changed little between 2007 and 2018 (**Table P in S3 Text**). However, when categorizing participants from all surveys by year of birth, there were progressively earlier mean age first started daily smoking across different birth cohorts, from 23.1 years among men born in the 1930s to 1940s to 17.4 years among men born in 1990s (*p* for trend <0.001; **Fig 3A and Table Q in S3 Text**). The proportion of smokers who reported smoking manufactured cigarettes was higher in urban than in rural men in each survey year and increased slightly over time, from 95.0% (95% CI: 93.7 to 96.2) in 2007 to 97.7% (95% CI: 97.0 to 98.4) in 2018 (*p* for trend <0.001; **Table R in S3 Text**). Across different birth cohorts included in all surveys, there was a progressive increase in the proportion of manufactured cigarette smokers (all *p* < 0.001), with the diminishing difference between urban and rural areas (*p* for difference = 0.04), especially among men born after 1990 (99.0% in urban versus 98.6% in rural; **Fig 3B and Table S in S3 Text**). Overall, there were few changes in the mean numbers of cigarettes smoked between 2007 and 2018 (17.9 [95% CI: 17.5 to 18.4] versus 17.2 [95% CI: 16.8 to 17.5] cigarettes/day, respectively; *p* for trend = 0.005; **Table T in S3 Text**). In general, rural men, older men, or those with poor education tended to smoke more in each survey. Across the different birth cohorts, men born in the 1960s tended to smoke more (19.8 [95% CI: 19.5 to 20.1] cigarettes/day) compared with other birth cohorts (*p* < 0.001; **Fig 3C and Table U in S3 Text**).

Among female smokers, there was also a decreasing trend in the mean age first started daily smoking across different birth cohorts, especially among urban women, from 30.5 years in the 1930s to 1940s down to 18.7 years in the 1990s birth cohort (*p* for trend <0.001; **Table Q in S3 Text**). Although the proportion of female smokers who smoked manufactured cigarettes only was generally lower than that observed among male smokers, it also increased steadily, particularly among rural women, from 72.1% (95% CI: 59.6 to 84.6) in 2007 to 93.0% (95% CI: 89.5 to 96.5) in 2018 (*p* for trend <0.001; **Table R in S3 Text**). Female smokers also tended to smoke less than their male counterparts (14.4 [95% CI: 11.6 to 17.2] versus 17.2 [95% CI: 16.8 to 17.5] cigarettes/day in 2018, *p* < 0.001; **Table T in S3 Text**).

### Regional variations in smoking prevalence

**Fig 4:**
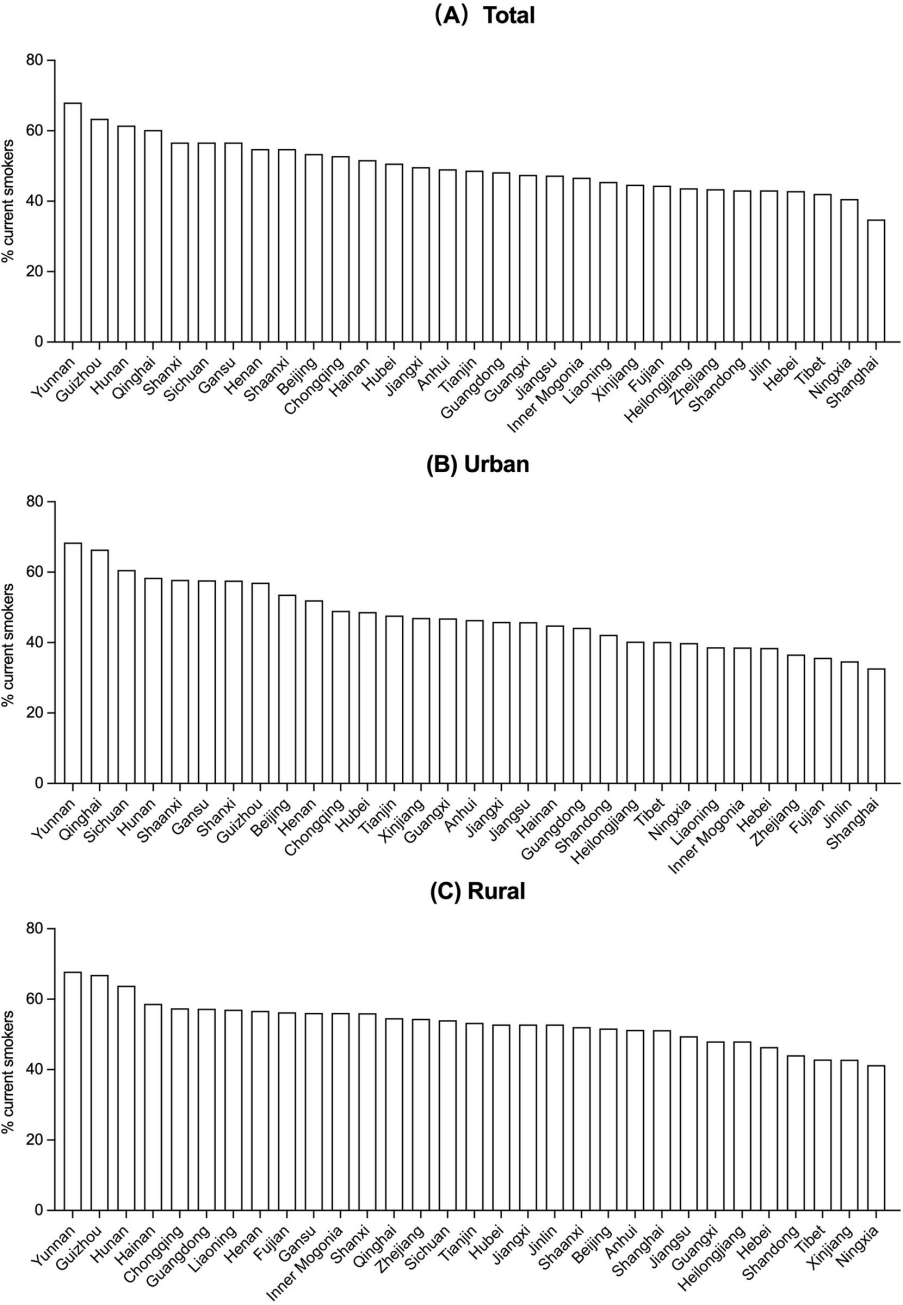
**Prevalence of current smoking among men aged ≥18 years in 2018, by province for overall (A), urban (B), and rural (C).** Data from the 2018 CCDRFS. CCDRFS, China Chronic Disease and Risk Factor Surveillance.

Among men, the prevalence of current smoking varied 2-fold across 31 provinces in 2018, from 34.8% (95% CI: 27.3 to 42.3) in Shanghai to 68.0% (95% CI: 63.0 to 73.1) in Yunnan (**Fig 4 and Table V in S3 Text**). Among women, it varied from 0.1% (95% CI: 0.0 to 0.1) in Hainan to 8.8% (95% CI: 6.0~11.6) in Jilin (**Fig B in S2 Text and Table V in S3 Text**). In general, provinces in southern China had a relatively higher male smoking prevalence than other provinces, with three southern provinces (Yunnan, Guizhou, and Hunan) that were the top three in per capita cigarette production in 2018 [20] having the highest smoking prevalence (68.0%, 63.4% and 61.5%, respectively) (**Tables V and W in S3 Text**). By contrast, provinces in north-eastern China had higher female smoking prevalence than other provinces, with three north-eastern provinces having the highest smoking prevalence (Jilin: 8.8%; Heilongjiang: 7.0%; and Liaoning: 6.5%), driven mainly by high prevalence among older generations (**Table V in S3 Text**).

### Estimated number of smokers in China

Based on 2018 China population data and the prevalence of current smoking (25.6%) and past smoking (5.0%) among adults aged ≥18 years in 2018, we estimated that overall in China, there were 282 million (95 CI: 272 to 292) current smokers (271 million men [95% CI: 262 to 281], 11 million women [95% CI: 10 to 12]) and 55 million [95% CI: 52 to 57] ex-smokers in 2018, including 264 million (95% CI: 254 to 273) smokers aged 18 to 69 years (255 million men [95% CI: 245 to 264], 9 million women [95% CI: 8 to 10]). Based on the mean reported number of cigarettes smoked, in total, these smokers consumed an estimated 1,545 billion (95% CI: 1,490 to 1,600) cigarettes in 2018, which were accounted for almost exclusively by men (1,497 billion cigarettes).

## Discussion

To the best of our knowledge, this is the first large nationwide study assessing the long-term trend in smoking prevalence in China since the implementation of WHO FCTC in 2005. Based on data from 5 large consecutive and nationally representative surveys between 2007 and 2018, the present study showed that the smoking prevalence among adult men in China, while remaining the world’s highest, had decreased steadily since 2007. However, the pace and patterns of change varied greatly between urban and rural areas and across different birth cohorts, with an increasing trend in smoking prevalence among rural men born in the 1990s. Moreover, there was a large difference in smoking prevalence across different provinces and a progressive decrease in mean age first start smoking daily and progressive increase in the proportion who smoked exclusively manufactured cigarettes. Among women in China, although the smoking prevalence remained extremely low, there appeared an upward trend among women born after the 1990s and those who were highly educated.

The Healthy China 2030 Plan set a specific tobacco control goal, which is to reduce the smoking prevalence among people aged over 15 years old to below 20% by 2030, with a “near-term goal” of prevalence not exceeding 25% by 2025 [21]. From 2007 to 2018, the overall smoking prevalence in Chinese adults aged 18 to 69 years had decreased by 13%, and in 2018, the smoking prevalence in China (26.7%) was lower than the global average (32.7%) [4]. Our pre-2015 results were generally consistent with previous reports in China, including those from the China Nutrition and Health Survey (CNHS) and National Health and Service Survey (NHSS) [7–9]. These nationwide studies showed the male smoking prevalence was highest before the mid-1990s at over 60%, then declined gradually for about 10 years before reaching a plateau of approximately 50% at around 2010 [7–9]. Although these 2 nationwide surveys included young people aged 15 to 17 years, they did not provide detailed information on the smoking patterns (e.g., age starting and types of tobacco smoked) nor data on apparent urban and rural difference and the large geographic variation in smoking prevalence across different parts of China. The latest NHSS conducted in 2013 [7] and the present study indicated if the current decreasing trend in smoking prevalence continues, then, China should be on track to reach the “near-term goal” by 2025. However, there were considerable differences between men and women, between urban and rural areas, and between different birth cohorts, highlighting the need for more targeted measures to further reduce smoking prevalence.

Our study showed among men, the pace of decrease in smoking prevalence appeared to have slowed in recent years, from 4.4% between 2007 and 2010 to 3.3% in the following 8 years. Of particular concern is the increasing trend in smoking prevalence among young men living in rural areas, which, if continues, will further slow or even reverse the overall decreasing trend among men in future decades. In high-income counties, while the overall smoking prevalence has been decreasing significantly in recent decades [4], individuals with low SES have higher smoking prevalence and these are declining more slowly [22]. The divergent trend between urban and rural men and the more sustained decreasing trend observed among men with high education observed in the present study was consistent with similar evident in certain other countries (e.g., US [23], Korea [24], and Finland [25]), suggesting that smoking will probably become a major public health concerns among people with low SES in China. The possible reasons for such transition are complex and may include poor access to—/or low levels of understanding and acceptance of—health knowledge among people with low SES, especially when effective tobacco control measures (e.g., effective package warnings) and regulations (e.g., high tobacco tax) are not in place or only partially implemented. On the other hand, however, we observed that the decreasing trends in smoking prevalence were more marked among men with service or professional occupations. This could be due in part to the ban on smoking in public places (including restaurants, trains, airlines) introduced in China since 2005, the year when FCTC was ratified. Moreover, the rising trend in smoking prevalence among rural men born during the 1990s appeared to have peaked in 2015, the year when regular increases in tobacco taxation were introduced.

Offering smoking cessation help is one of the essential tobacco control strategies recommended by WHO FCTC to reduce the future burden of smoking-related mortality and morbidity. There was evidence from large prospective studies that a high proportion of ex-smokers in China did not quit smoking voluntarily, but rather because of poor health, and were at a higher risk of subsequent mortality due to preexisting diseases [5]. Although the rate of smoking cessation among male smokers slightly increased between 2007 and 2019, currently only approximately 15% smokers in the present study had quit smoking, which is about half of that observed in the USA (>30%) [26]. Moreover, we observed no clear temporal trends in smoking cessation in smokers suffering from major NCDs. In recent decades, China has introduced various NCD prevention guidelines, including establishment of nationwide smoking quit-line service and cessation clinics to help smokers to quit, especially those diagnosed with NCDs. However, these services have not been used widely due in part to inadequate funding support, lack of qualified staff, and limited access by the general population [21,27]. Moreover, there were significant differences in service levels according to a 2019 survey covering 234 smoking cessation clinics in 23 provinces [28]. As in most previous studies, we did not collect detailed information about the severity of the diagnosed NCDs nor the likely reasons and how many times tried for smoking cessations previously, which could inform the development of intervention strategies and policies that better promote smoking cessation in the hospital setting and patients suffering from NCDs.

The present study showed that among men, there was a 2-fold difference in smoking prevalence across 31 provinces in 2018, with the prevalence particularly high in regions with high tobacco production (i.e., Yunnan, Guizhou, and Hunan). Among women, however, the geographic variation in smoking prevalence was not correlated with that in men. Indeed, in the 3 provinces with the highest male smoking prevalence and a high production of tobacco, the female smoking prevalence were all <2%. In contrast, the 2 provinces in Northeast China (i.e., Jilin and Heilongjiang) had a female smoking prevalence higher than 6%, but the male smoking in these 2 provinces was lower than in most other provinces. These findings suggest that the reasons underlying smoking uptake rates both now and in the past differed importantly between men and women and may also vary between different birth cohorts. There is good evidence from the present and previous studies that female smoking prevalence has declined progressively across different birth cohorts well before the introduction of tobacco control measures, from around 30% among women born during 1920s to 1940s to <5% among women born in 1960s to 1970s in certain parts of China (e.g., Heilongjiang and other northeastern regions), for reasons that are still poorly understood [5] and can only be speculated. During 1950s and 1980s, there were limited availabilities of manufactured cigarettes and no tobacco advertising in China due to introduction and implementation of the planned economy, in contrast to the situations before 1950. While making men less likely to start early in adult life and more likely to smoke non-cigarette tobacco products, these factors may have also helped to drive down the smoking prevalence among women as well as to change the acceptable social norm associated with female smoking over time. After 1980s, although manufactured cigarette has gradually become more widely available and affordable with introduction of the market economy, China passed a law to ban tobacco advertising during early 1990s, mainly to protect domestic market, which may have helped to maintain low smoking uptake rate among young women in China. Given the more than 20-fold difference in smoking prevalence between men and women in China, smoking is, and will continue to be, one of the important factors for large sex difference in adult mortality.

In the past few decades, smoking continued to be the major risk factor for a wide range of NCDs [29,30]. However, most high-income countries have witnessed a progressive and significant decline in smoking prevalence in adult populations, in line with various tobacco control measures introduced at different time points [4]. In the United Kingdom, for example, smoking prevalence declined significantly before the 1970s, decelerated between 1973 and 2000, then accelerated again after the introduction of the National “Smoking Kills” tobacco control plan, with nearly a linear decreased prevalence at about 0.7% per year. These findings indicated that the decline in smoking prevalence was responsive to major tobacco control initiatives [31]. In China, tobacco control has gradually gained the government’s and society’s attention since the 1970s. Although the FCTC was officially ratified in 2005 by the Chinese government, China has not yet issued national legislation targeting specially on key FCTC recommended tobacco control measures. In 2007, China launched the National Healthy Lifestyle Action, which included a non-smoking initiative, and by 2018, it had covered about 90% of all rural counties or urban districts. The stabilized smoking prevalence in recent years, as demonstrated in the present study, indicate that the effects of these policies or measures are not satisfactory. China urgently needs a unified and comprehensive tobacco control law that covers a wide range of policies and regulations addressing issues such as steadily increasing taxation cigarettes, smoke-free workplaces and indoor public places, large and effective graphic warnings on the cigarette pack, and cessation services. Without these effective measures, the current decreasing trend in smoking prevalence is not likely to continue in the coming years and decades.

### Strengths and limitations

Apart from large and nationally representative samples, each CCDRFS survey followed a standard study protocol, sampling method, core questionnaire, and quality control procedures that ensured the continuity and comparability of results over the years. Moreover, we were able to provide detailed analyses not only for the population overall but also by sex, regions, education, and occupations, revealing striking and large variations and divergent trends.

Nevertheless, the present study has limitations. Firstly, since CCDRFS is mainly concerned with NCDs in adults and their main risk factors, children or teenagers under age 18 were not included. Also, because of the limited resources, surveys were not done on an annual basis and those conducted before 2010 included only adults aged 18 to 69 years (according to the recommendation of WHO STEPS), and to ensure consistency across all 5 surveys, the main analyses were restricted only to people aged 18 to 69 years. Second, we did not use smoking-related biomarkers (e.g., exhaled CO [32] or blood cotinine) to objectively validate the accuracy of self-reported smoking prevalence, so it is possible that smoking prevalence could be underestimated due to reporting bias. However, previous studies demonstrated a high concordance between self-reported smoking behaviors and certain objectively assessed measures [33]. Moreover, all the interviews took place at home and covered not only smoking but also a wide range of other lifestyle factors, so it is unlikely the participants would deliberately underreport their smoking habits due to societal peer pressure. Third, for certain smoking categories, including smoking cessation, the definitions used in each survey changed slightly over time and also differed somewhat from those used in previous studies in China and elsewhere, making the direct comparisons difficult. Fourth, the history of NCDs diagnosis was self-reported, hence recall bias may exist. Fifth, we used linear regression to estimate the rate of annual changes during 2007 and 2018, both overall and in certain key subgroups. While appropriate in most cases, for certain subgroups, the approach may not generate most reliable estimates because the trends appeared nonlinear. For example, among rural men born during 1990s, there was a clear upward and apparently linear trend between 2007 and 2015 but a drop in prevalence between 2015 and 2018. However, since the decreasing trend only covered 2 time points, we were not able to estimate reliably the rate of change post-2015. Further surveys after 2018 should help to confirm nonlinearity of certain trends.

### Implications

Findings from our study showed that in China, smoking prevalence has decreased steadily in recent decades, but there were diverging trends between urban and rural areas, especially among men born after 1980. Future tobacco control strategies should target more specifically rural young men, regions with high tobacco production, and smokers who suffered from NCDs.

## Conclusions

In summary, the present study showed that while smoking prevalence had decreased steadily in China from 2007 to 2018, this trend was not uniform by sex, areas, birth cohorts, educational attainment, and occupation. Of particular concern is the rising trend in prevalence among men born after 1990, especially in rural areas. As well as nationwide legislation to fully implement various key measures recommended by the WHO FCTC (e.g., appropriate package warning and the significant increase in tobacco tax), future smoking prevention and intervention efforts may need to target more specifically at rural areas, regions with high tobacco production, and young people. Moreover, as part of secondary prevention, patients diagnosed with NCDs should be strongly encouraged to quit smoking, with adequate professional support and cessation advice along with necessary nicotine replacement therapies if needed.

## Supporting information

S1 Text Supporting appendix. Appendix A in S1 Text. Study design and sampling procedures of CCDRFS during 2007–2018. **Appendix B in S1 Text.** Data collection of CCDRFS during 2007–2018. **Appendix C in S1 Text.** Analysis plan.
(DOCX)

S2 Text Supporting figures. Fig A in S2 Text. Flow diagram of study design and sampling procedure of CCDRFS 2007–2018. **Fig B in S2 Text.** Regional variation in prevalence of current smoking among women aged ≥18 years in 2018. **Fig C in S2 Text.** Number of manufactured cigarettes produced annually in China from 2000–2020.
(DOCX)

S3 Text Supporting tables. Table A in S3 Text. Specific questions related to smoking in CCDRFS 2007–2018. **Table B in S3 Text.** The prevalence of current smoking among men in China, 2007–2018. Values are weighted percentages (95% CI). **Table C in S3 Text.** The prevalence of regular smoking among men in China, 2007–2018. Values are weighted percentages (95% CI). **Table D in S3 Text.** The prevalence of occasional smoking among men in China, 2007–2018. Values are weighted percentages (95% CI). **Table E in S3 Text.** The prevalence of former smoking among men in China, 2007–2018. Values are weighted percentages (95% CI). **Table F in S3 Text.** The prevalence of current smoking among men and women living in poor and non-poor rural areas in China, 2007–2018. Values are weighted percentages (95% CI). **Table G in S3 Text.** The prevalence of current smoking among men and women by area, age, and year of birth in China, 2007–2018. Values are weighted percentages (95% CI). **Table H in S3 Text.** The prevalence of current smoking among women in China, 2007–2018. Values are weighted percentages (95% CI). **Table I in S3 Text.** The prevalence of regular smoking among women in China, 2007–2018. Values are weighted percentages (95% CI). **Table J in S3 Text.** The prevalence of occasional smoking among women in China, 2007–2018. Values are weighted percentages (95% CI). **Table K in S3 Text.** The prevalence of former smoking among women in China, 2007–2018. Values are weighted percentages (95% CI). **Table L in S3 Text.** Percentages of former smokers among ever smokers among men and women in China, 2007–2018. Values are weighted percentages (95% CI). **Table M in S3 Text.** The prevalence of current smoking among adults with and without major NCDs in China, 2007–2018. Values are weighted percentages (95% CI). **Table N in S3 Text.** The prevalence of current and former smoking among men with at least 1 NCD or specific major NCDs, 2007–2018. **Table O in S3 Text.** Mean age of participants with at least 1 NCD or specific NCD across 5 CCDRFS. **Table P in S3 Text.** Age first started daily smoking among current regular smokers among men and women in China, 2007–2018. Values are weighted means (95% CI). **Table Q in S3 Text.** Age first started daily smoking among current regular smokers by sex, area, and year of birth. Values are weighted means (95% CI). **Table R in S3 Text.** Percentages of manufactured cigarette smokers among current regular smokers among men and women in China, 2007–2018. Values are weighted percentages (95% CI). **Table S in S3 Text.** Percentages of manufactured cigarette smokers among current regular smokers by sex, area, and year of birth. Values are weighted percentages (95% CI). **Table T in S3 Text.** Daily number of cigarettes smoked among current regular smokers among men and women in China, 2007–2018. **Table U in S3 Text.** Daily number of cigarettes smoked among current regular smokers by sex, residence, and year of birth. Values are weighted means (95% CI). **Table V in S3 Text.** The prevalence of current smoking among adults aged ≥18 years by province in China in 2018. Values are weighted percentages (95% CI). **Table W in S3 Text.** The cigarettes production by province in China in 2018*.
(DOCX)

S1 STROBE Checklist STROBE checklist. (DOCX)

## Abbreviations

CCDRFS: China Chronic Disease and Risk Factor Surveillance
CI: confidence interval
CNHS: China Nutrition and Health Survey
COPD: chronic obstructive pulmonary disease
DALY: disability-adjusted life year
NCD: noncommunicable disease
NHSS: National Health and Service Survey
WHO FCTC WHO: Framework Convention for Tobacco Control
