# Supplementary material for: Trends in smoking prevalence in urban and rural China, 2007 to 2018: Findings from 5 consecutive nationally representative cross-sectional surveys
Source: PLoS Med. 2022 Aug 25;19(8):e1004064. doi: 10.1371/journal.pmed.1004064 (PMC9409540; doi:10.1371/journal.pmed.1004064)
Supplement: S1 Text — Study design and sampling procedures of CCDRFS during 2007–2018. Appendix B in S1 Text. Data collection of CCDRFS during 2007–2018. Appendix C in S1 Text. Analysis plan. (DOCX) [file pmed.1004064.s001.docx]

**S1 TEXT: SUPPORTING APPENDIX**

# **Appendix A. Study design and sampling procedures of CCDRFS during 2007-2018**

**Overview**

The sample of CCDRFS was obtained from China’s national Disease Surveillance Points (DSPs) system [1]. One DSP unit covers a rural county or an urban district. The DSPs system were established during the early 1980s, using multi-stage stratified sampling with probability proportional to size (PPS) to ensure the representativeness of the national population of mainland China. Following further expansion and sampling enhancement, from the 2013 survey the selected DSPs were also representative at the provincial level. CCDRFS is planned and administrated by the National Center for Chronic and Non-communicable Disease Control and Prevention (NCNCD) of the Chinese Center for Disease Control and Prevention (CDC).

**Disease Surveillance Points system**

The DSPs system was piloted in 1978 and fully established in early 1980s covering initially 71 sites, expanding to 145 DSPs in 1989 and 161 DSPs in 2004 (covering a population of 73 million), in order to accommodate the societal and economic development during this period. For each expansion, the population characteristics of the selected DSPs were compared to the census population to ensure its nationally representativeness [2, 3]. In 2013, the Chinese government combined the DSPs system with the national vital registration system to form an integrated national mortality surveillance system, which increased the DSPs areas from 161 to 605. The DSPs system currently covers 324 million Chinese adults (24% of all Chinese population) in the mainland of China.

**Sampling procedure**

Embedded within the DSP system, the CCDRFS used stratified multi-stage cluster sampling to generate a nationally representative sample for each survey.

In the first stage, half of the DSPs were selected as primary sampling units (PSUs) using stratified sampling (in the surveys of 2013, 2015 and 2018), or all DSPs were selected as PSUs (in the surveys of 2007 and 2010). Since the 2013 survey, following the expansion of the DSPs system, 298 DSPs were selected from all 605 DSPs to generate a sample representative of both the national and provincial population in the 31 provinces/autonomous regions/municipalities of mainland China. The sampling of DSPs system was done centrally by the NCNCD.

Within each selected DSP, the following sampling steps were followed:

1. Townships (rural) or subdistricts (urban) were selected with the PPS sampling in each selected DSP.
2. Within the selected townships and subdistricts, villages (rural) or residential areas (urban) were selected with the PPS sampling.
3. Each selected village or residential area was divided into groups of about 40 to 60 households (40 households in 2007 and 2010, 50 household in 2013 and 60 households since 2015), based on existing villager/resident groups in the village or residential area. One group was selected with random sampling. Before 2015, all households in the group selected were invited to participant the survey. Since 2015, 45 household were randomly selected from the selected group (60 households).
4. Within each selected household, the Kish method was used to select one eligible adult in the 2007, 2010 and 2013 surveys; while all eligible adults in that household were invited to participate in the 2015 and 2018 surveys.

Eligibility criteria for each survey include:

- 1. Aged 18 years or older;
  2. Having lived in the address for more than 6 months in the past 12 months;
  3. Not pregnant;
  4. Not having a serious health condition or illness that prevents from participating, including intellectual disability or language disorder.

1. In a household, if the selected eligible adult (for 2007, 2010, and 2013 surveys), or more than half of the family’s eligible members (for the 2015 and 2018 surveys) refused to participate or were not reachable, a replacement household with the similar sex and age structure was selected. In each survey, <10% households were replaced.

For surveys between 2007 and 2013, the sampling within selected DSPs, as described above, was conducted by the NCNCD centrally; since the 2015 survey, the sampling was performed by the provincial CDCs and approved by the NCNCD.

**Sample size calculation**

Sample size of each round of CCDRFS was calculated using N =

where *u* = 1.96 (corresponding to 95% confidence level), *deff* is design effects (1.5 to 3.5), *r* is relative error (20%), and *d* is margin of error (*d*= *r* × *p*). The *p* is the prevalence of factor studied for calculation. By using obesity prevalence (2004/2007) [4, 5] or diabetes prevalence (2010/2013) from previous national surveys (7-10.4%) [6, 7], the table below shows the calculated target sample sizes in each survey. Since the previous smoking prevalence was higher than the prevalence of obesity or diabetes in each survey year, the calculated sample size could satisfy the accurate estimation of smoking rate.

| Survey | Indicators | Prevalence | Target sample size |
| --- | --- | --- | --- |
| 2007 | Obesity | 7% in 2004 | 52,020 |
| 2010 | Obesity | 8.0% in 2007 | 96,870 |
| 2013 | Diabetes | 9.7% in 2010 | 181,000 |
| 2015 | Diabetes | 9.7% in 2010 | 184,773 |
| 2018 | Diabetes | 10.4% in 2013 | 181,059 |

**Sample weights**

Across all CCDRFS surveys, we developed sample weights to account for multi-stage sampling design, post-stratification and, for the 2015 and 2018 surveys only, non-response within household as all members were selected. For an individual in the sample, his/her sample weights were developed as follows.

1. Base weights for multi-stage design (W_design_)

W_design_ = W_d1_ × W_d2_ × W_d3_ × W_d4_ × W_d5_ × W_d6_

- W_d1_ is the total number of counties (rural) or districts (urban) in the stratum divided by the number of selected DSPs (Primary Sampling Unit) in the stratum where the individual was from;
- W_d2_ is the total number of townships (rural) or subdistricts (urban) in the PSU where the individual belonged divided by the number of selected townships or subdistricts;
- W_d3_ is the total number of villages (rural) or residential areas (urban) in the township or subdistrict where the individual was from divided by the number of selected villages or residential areas;
- W_d4_ is the total number of groups in the village or residential area where the individual was from;
- W_d5_ is the total number of households in the group where the individual belonged divided by the number of selected households in the group where the individual was from.
- W_d6_ is the total number of eligible adults in the household where the individual was from (2013 or earlier) or 1 (since 2015).

1. Non-response weights (W_nr_) in the 2015 and 2018 surveys

W_nr_ = the number of eligible adults in the household where the individual was from divided by the number of participating adults in the household.

1. Post-stratification weights (W_ps_)

Stratifications included: province (31 levels), urban or rural (2 levels), sex (2 levels), age group (10 levels: 18-24, 25-29, 30-34, 35-39, 40-44, 45-49, 50-54, 55-59, 60-64, 65-69). The 2010 census population was also stratified in the same way. In k^th^ stratum, the post-stratification weights (Wps,k) are:

$$Wps,k= \frac{Population in the k^{\mathrm{th}} stratum of the 2010 census population}{Sum of Wdesign\times Wnr for all individuals in the k^{\mathrm{th}}\mathrm{stratum}}$$

# **Appendix B. Data collection of CCDRFS during 2007-2018**

**Overview**

CCDRFS field work was coordinated by the NCNCD and implemented by the local CDCs. All filed staff received mandatory training provided by certified instructors from the NCNCD and hosted by provincial CDCs.

The field work in each round of survey started in August and finished in the same year (survey 2007) or before summer of the next year (surveys 2010, 2013, 2015 and 2018). Trained interviewers from local CDCs carried out the face-to-face interview, physical measurement, and biochemical sample collection and pre-treatment. Interview was conducted during a home visit. Physical measurement and biochemical sample collection were arranged at a community health centre.

**Home visits**

During the home visit, the head of household or an adult who knew well the details of the household (e.g., economic and environmental information) was interviewed firstly using the household questionnaire that assessed the eligibility of all household members. Then, all eligible subjects were given a personal questionnaire that covered demographic characteristics, lifestyle factors (e.g., smoking and cessation, diet behavior, physical activity, alcohol consumption), and the history of chronic diseases. At the end of the home visit, the investigator gave each eligible member an appointment letter with the instruction of the location, time and precautions of the physical measurements.

**Physical measurements**

All subjects were invited to a community health centre to attend a physical measurement session. Participants who did not complete their personal interviews during home visits also completed their interviewers in the health centre. Weight, height, waist circumference, and blood pressure were measured using a standard protocol for each survey.

**Data management**

From 2007 to 2013, all data were recorded on paper questionnaires first, and then entered into computer via a bespoke data management system and delivered to the NCNCD via email or an online data transfer system. All the data entry and processing were done by the local CDCs, except in 2007 when the data entry was outsourced to a company. Since 2015, NCNCD deployed an integrated electronic platform consisting of a tablet-assisted interview system and an online information management system, which was adopted by all local CDCs. The data of the questionnaire and physical measurement was collected electronically using the tablet-assisted system. The internet-based information management system was used to generate samples (as described in Appendix 1), on-site identity confirmation, quality control, physical examination reports, and data download.

# **Appendix C. Analysis plan.**

The aim of the present study is to describe the trends from 2007 to 2018 in smoking prevalence and cessation as well as the smoking patterns in mainland China, overall and by subgroups of Chinese population.

**Study participants**

Men and women aged 18-69 years

**Main outcomes**

Prevalence of smoking (ever, current, and regular)

**Secondary outcomes**

Patterns of smoking among smokers, defined by a) mean age first started daily smoking, b) mean numbers of cigarettes smoked per day, and c) proportions of cigarette smokers in current regular smokers, d) proportion of smoking cessation among ever smokers, e) estimated number of smokers.

**Stratification**

**Sex** (men, women), **age** (18-29, 30-39, 40-49, 50-59, 60-69 years), **year of birth** (1930-1940s, 1950s, 1960s, 1970s, 1980s, 1990s), **area of residence** (rural, urban), **education** (no formal or primary school, secondary school, high school, college or university), **region** (North, East, Northeast, Middle, South, Southwest, Northwest), **occupation** (agriculture, manufacture, service provider, managers or professionals, others, unemployed or students, retired), **self-reported noncommunicable diseases** (hypertension, diabetes, stroke, myocardial infarction, chronic obstructive pulmonary disease).

All of 31 provinces in mainland China are categorized into 7 regions:

- North: Beijing, Tianjin, Hebei, Shanxi, Inner Mongolia
- Northeast: Liaoning, Jilin, Heilongjiang
- East: Shanghai, Jiangsu, Zhejiang, Anhui, Fujian, Jiangxi, Shandong
- Middle: Henan, Hubei, Hunan
- South: Guangdong, Guangxi, Hainan
- Southwest: Chongqing, Sichuan, Guizhou, Yunan, Tibet
- Northwest: Shaanxi, Gansu, Qinghai, Ningxia, Xinjiang

Interactions of grouping are limited to sex plus one other group.

**Statistics**

Outcome (main and secondary) levels, overall and in subgroups as defined in ‘Stratification’.

- SAS is used to account for clustering, stratification, and sample weights in the sample design.
- For the prevalence and means, standard error and 95% confidence interval are estimated using *proc* *surveylogistic*  procedure and *proc surveymeans* procedure in SAS, which use Taylor series linearisation and accounts for finite population correction [8, 9].

Change in outcome (main and secondary) levels from 2007 to 2018, overall and in groups as defined in ‘Change’.

- Annual rate of change in prevalence and means:

$\mathrm{annual}rate of \mathrm{change}=\left( \frac{{Level}_{2018}}{{Level}_{2007}} \right)^{\frac{1}{t_{2018}-t_{2007}}}$-1

We used the Horvitz–Thompson estimator to compute the population totals ant their variance for current and former smokers due to the complex sample design of 2018 survey. In the case of the current survey design including stratification (with strata indexed by h = 1, …, H) and clustering (with clusters within stratum h indexed by α = 1, 2, …, ah), the simple weighted estimator for the current smoker total can be written as follows:

$$\hat{\gamma}=\sum_{h=1}^{H} \sum_{\alpha=1}^{a_{h}} \sum_{i=1}^{n_{h\alpha}} w_{hai}y_{hai}$$

Where w_hai represents the complex weight for the i_th individual in cluster α within stratum h. y_hai is a binary indicator for current smoking (1=yes, 0=no).

Population totals and their 95% CIs can be promptly calculated in the procedures that support complex sample analysis from SAS and other software systems.

**Missing data**

Individuals with missing information on smoking or key demographic factors are excluded from the analyses.

**References**

1 Yang G, Hu J, Rao KQ, Ma J, Rao C, Lopez AD. Mortality registration and surveillance in China: History, current situation and challenges. Popul Health Metr. 2005; 3: 3.

2 Yang G. [Selection of DSP points in second stage and their presentation]. Zhonghua Liu Xing Bing Xue Za Zhi 1992; 13: 197-201. (in Chinese)

3 Zhou MG, Jiang Y, Huang ZJ, Wu F. Adjustment and representativeness evaluation of national disease surveillance points system. Dis Surveill 2010; 25: 239-44.

4 National Centre for Chronic and Non-communicable Disease Control and Prevention, Chinese Centre for Disease Control and Prevention. Report on the chronic disease and risk factors among Chinese adults (2004). Beijing. People's Medical Publishing House, 2007. (in Chinese)

5 National Centre for Chronic and Non-communicable Disease Control and Prevention, Chinese Centre for Disease Control and Prevention. Report on the chronic disease and risk factors among Chinese adults (2010). Beijing. Military Medical Press, 2012. (in Chinese)

6 Xu Y, Wang L, He J et al. Prevalence and control of diabetes in Chinese adults. JAMA. 2013;310(9): 948-59.

7 Wang L, Gao P, Zhang M et al. Prevalence and Ethnic Pattern of Diabetes and Prediabetes in China in 2013. JAMA. 2017;317(24):2515-23.

8 SAS Institute Inc. SAS/STAT 14.3 User’s Guide The SURVEYLOGISTIC Procedure. SAS/STAT 143 User’s Guide. Cary, NC: SAS Institute Inc.; 2017.

9 SAS Institute Inc. SAS/STAT 14.3 User’s Guide The SURVEYMEANS Procedure. SAS/STAT 143 User’s Guide. Cary, NC: SAS Institute Inc.; 2017.
