# Supplementary material for: Trends in smoking prevalence in urban and rural China, 2007 to 2018: Findings from 5 consecutive nationally representative cross-sectional surveys
Source: PLoS Med. 2022 Aug 25;19(8):e1004064. doi: 10.1371/journal.pmed.1004064 (PMC9409540; doi:10.1371/journal.pmed.1004064)
Supplement: S2 Text — Flow diagram of study design and sampling procedure of CCDRFS 2007–2018. Fig B in S2 Text. Regional variation in prevalence of current smoking among women aged ≥18 years in 2018. Fig C in S2 Text. Number of manufactured cigarettes produced annually in China from 2000–2020. (DOCX) [file pmed.1004064.s002.docx]

**S2 TEXT: SUPPORTING FIGURES**


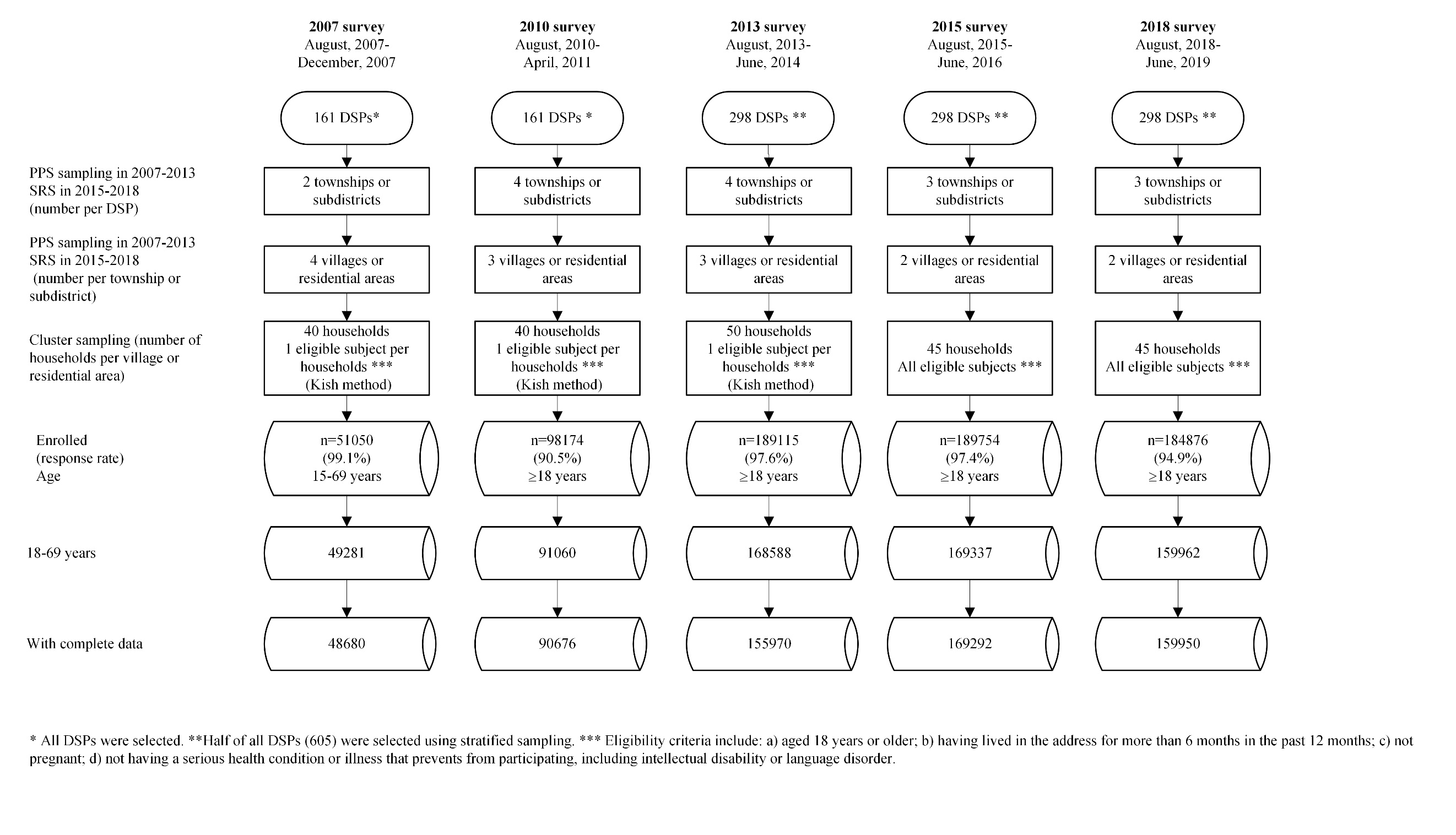


# **Fig A. Flow diagram of study design and sampling procedure of CCDRFS 2007-2018.** CCDRFS, China Chronic Disease and Risk Factor Surveillance; DSPs, Disease Surveillance Points; PPS, probability proportional to size; SRS, systematic random sampling. Biochemical data were available since 2010 survey.


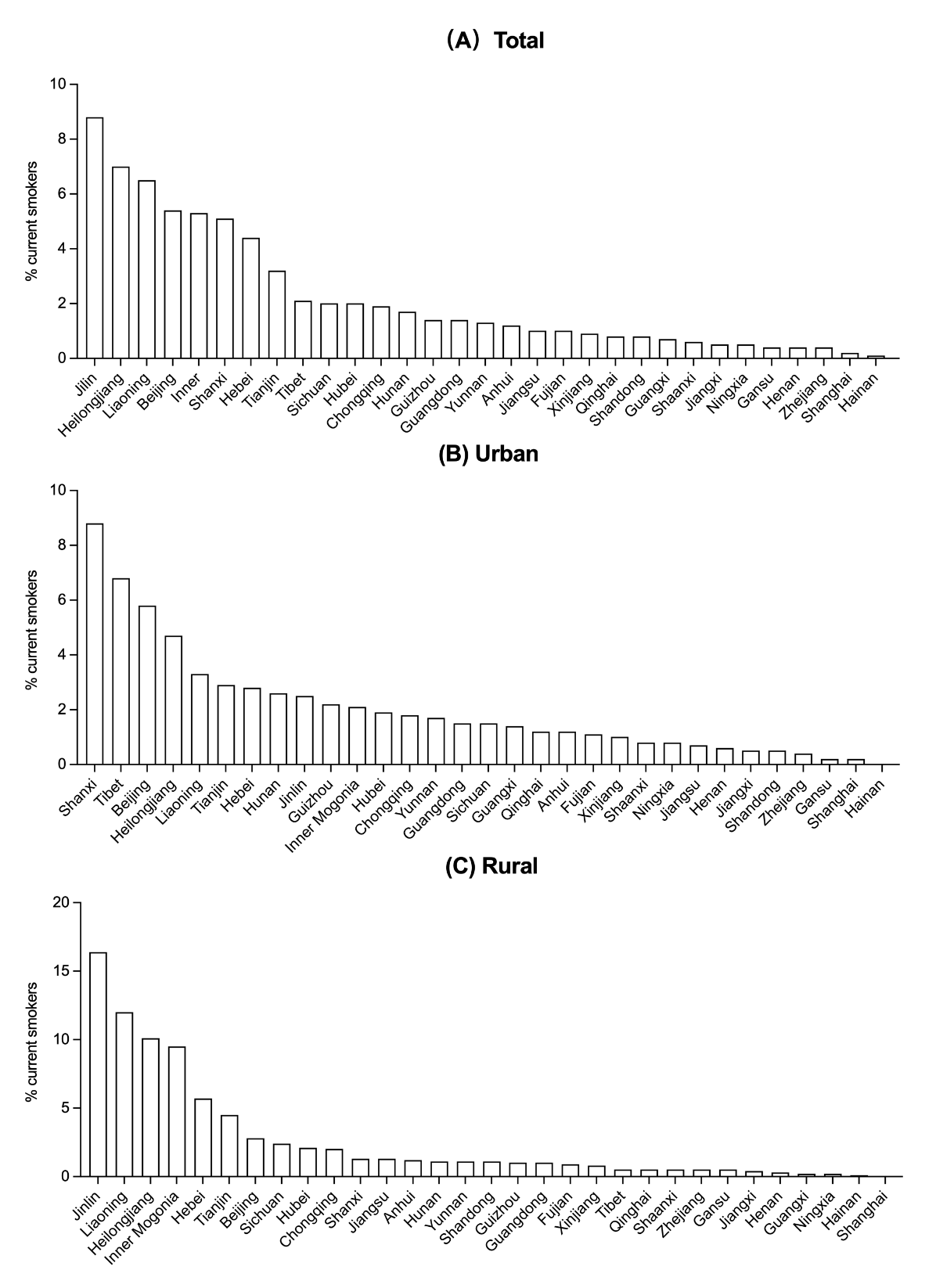


# **Fig B. Prevalence of current smoking among women aged ≥ 18 years in 2018, by province and area of residence.** Date from the 2018 CCDRFS. CCDRFS: China Chronic Disease and Risk Factor Surveillance.

# **Fig C. Number of manufactured cigarettes produced annually in China from 2000 to 2020.** Data were available from the web site of National Bureau of Statistics [cited 2021 2/12/21]: https://data.stats.gov.cn/easyquery.htm?cn=C01.
