# Supplementary material for: Trends in smoking prevalence in urban and rural China, 2007 to 2018: Findings from 5 consecutive nationally representative cross-sectional surveys
Source: PLoS Med. 2022 Aug 25;19(8):e1004064. doi: 10.1371/journal.pmed.1004064 (PMC9409540; doi:10.1371/journal.pmed.1004064)
Supplement: S3 Text — Specific questions related to smoking in CCDRFS 2007–2018. Table B in S3 Text. The prevalence of current smoking among men in China, 2007–2018. Values are weighted percentages (95% CI). Table C in S3 Text. The prevalence of regular smoking among men in China, 2007–2018. Values are weighted percentages (95% CI). Table D in S3 Text. The prevalence of occasional smoking among men in China, 2007–2018. Values are weighted percentages (95% CI). Table E in S3 Text. The prevalence of former smoking among men in China, 2007–2018. Values are weighted percentages (95% CI). Table F in S3 Text. The prevalence of current smoking among men and women living in poor and non-poor rural areas in China, 2007–2018. Values are weighted percentages (95% CI). Table G in S3 Text. The prevalence of current smoking among men and women by area, age, and year of birth in China, 2007–2018. Values are weighted percentages (95% CI). Table H in S3 Text. The prevalence of current smoking among women in China, 2007–2018. Values are weighted percentages (95% CI). Table I in S3 Text. The prevalence of regular smoking among women in China, 2007–2018. Values are weighted percentages (95% CI). Table J in S3 Text. The prevalence of occasional smoking among women in China, 2007–2018. Values are weighted percentages (95% CI). Table K in S3 Text. The prevalence of former smoking among women in China, 2007–2018. Values are weighted percentages (95% CI). Table L in S3 Text. Percentages of former smokers among ever smokers among men and women in China, 2007–2018. Values are weighted percentages (95% CI). Table M in S3 Text. The prevalence of current smoking among adults with and without major NCDs in China, 2007–2018. Values are weighted percentages (95% CI). Table N in S3 Text. The prevalence of current and former smoking among men with at least 1 NCD or specific major NCDs, 2007–2018. Table O in S3 Text. Mean age of participants with at least 1 NCD or specific NCD across 5 CCDRFS. Table P in S3 Text. Age first [file pmed.1004064.s003.docx]

**S3 TEXT: SUPPORTING TABLES**

# **Table A. Specific questions related to smoking in CCDRFS 2007-2018**

|  | **CCDRFS 2007/2010/2013/2015/2018** |
| --- | --- |
| **Smoking status** | **Do you currently smoke tobacco every day, some days or not at all?**   - Never (never smoker) - Not currently, but in the past (former smoker) - Yes, some days (current occasional smoker) - Yes, every day (current regular smoker) |
| **Age started daily smoking** | **How old were you when you first began daily smoking?** |
| **Number of cigarettes smoked per day** | **On average, how many manufacture cigarettes do you smoke per day/week?**   - _ cig./day (ask regular smoker) - _ cig./week (ask occasional smoker) - Don’t smoke manufacture cigarette |
| **Proportion of smokers smoking manufacture cigarettes** | **On average, how many manufacture cigarettes do you smoke per day/week?**   - _ cig./day (ask regular smoker) - _ cig./week (ask occasional smoker) - Don’t smoke manufacture cigarette |

# **Table B. The prevalence of current smoking among men in China, 2007-2018. Values are weighted percentages (95% CI).**

|  | **2007** | **2010** | **2013** | **2015** | **2018** | **Annual rate of change** | ***p* for trend** |
| --- | --- | --- | --- | --- | --- | --- | --- |
| **Overall** | 58.4（56.1~60.7） | 54.0（52.2~55.8） | 53.1（51.6~54.6） | 53.3（51.9~54.7） | 50.8（49.1~52.5） | -1.1^*^ | <0.001 |
| **Age, y** |  |  |  |  |  |  |  |
| 18-19 | 33.6（24.1~43.1） | 36.3（31.7~41.0） | 29.0（23.7~34.3） | 40.3（34.1~46.6） | 31.6（23.7~39.6） | -0.2 | 0.92 |
| 20-24 | 43.4（38~48.9） | 46.6（42.6~50.6） | 47.8（43.9~51.7） | 50.1（46.2~54.1） | 45.2（40.0~50.3） | 0.6 | 0.47 |
| 25-29 | 57.0（52.8~61.1） | 49.1（45.8~52.5） | 54.8（51.8~57.7） | 49.3（45.6~53） | 50.6（46.4~54.7） | -0.8 | 0.06 |
| 30-39 | 62.5（60.2~64.9） | 56.6（54.4~58.8） | 52.8（51.1~54.5） | 52.9（50.8~55.0） | 50.8（48.4~53.3） | -1.8 | <0.001 |
| 40-49 | 66.2（63.9~68.5） | 59.4（57.4~61.4） | 57.1（55.5~58.7） | 56.3（54.5~58.2） | 53.5（51.7~55.3） | -1.8 | <0.001 |
| 50-59 | 63.3（61.0~65.6） | 58.4（56.6~60.3） | 57.9（56.3~59.4） | 58.5（57.0~59.9） | 56.0（54.5~57.5） | -0.9 | <0.001 |
| 60-69 | 54.0（51.1~56.9） | 50.8（48.4~53.3） | 50.4（48.7~52.2） | 50.5（48.9~52.1） | 50.1（48.3~51.8） | -0.6 | 0.07 |
| **Birth cohort** |  |  |  |  |  |  |  |
| 1930-40s | 56.2（53.0~59.5） | 50.3（47.8~52.8） | 47.4（45.2~49.6） | 45.7（43.6~47.7） | 44.7（41.3~48.1） | -2.0 | <0.001 |
| 1950s | 64.7（62.5~66.9） | 58.1（56.2~60.1） | 56.6（55.1~58.1） | 55.3（53.8~56.9） | 51.4（49.7~53.0） | -1.9 | <0.001 |
| 1960s | 65.3（63.1~67.4） | 59.5（57.4~61.5） | 57.9（56.4~59.5） | 58.5（57.0~59.9） | 55.4（53.8~56.9） | -1.3 | <0.001 |
| 1970s | 62.1（59.5~64.6） | 57.5（55.6~59.5） | 54.6（52.9~56.3） | 54.3（52.5~56.1） | 53.3（51.5~55.2） | -1.4 | <0.001 |
| 1980s | 43.6（38.4~48.8） | 48.0（44.9~51.2） | 52.8（50.3~55.3） | 51.5（49.2~53.8） | 50.6（48.0~53.2） | 1.4 | 0.008 |
| 1990s | - | 38.2（34.0.0~42.4） | 41.6（37.6~45.6） | 48.6（45.0~52.2） | 45.3（41.4~49.3） | 2.4* | 0.02 |
| **Areas** |  |  |  |  |  |  |  |
| Urban | 55.7（51.2~60.3） | 52.8（49.9~55.7） | 48.6（46.5~50.8） | 48.3（46.2~50.4） | 46.3（43.7~49.0） | -1.7 | <0.001 |
| Rural | 59.9（57.5~62.4） | 54.7（52.4~57.1） | 55.6（53.8~57.5） | 56.9（55.3~58.5） | 54.6（52.6~56.6） | -0.6 | 0.05 |
| **Region** |  |  |  |  |  |  |  |
| North | 54.2（48.7~59.6） | 53.6（51.1~56.1） | 52.4（49.0~55.7） | 53.8（50.5~57） | 49.0（45.3~52.7） | -0.7 | 0.24 |
| Northeast | 58.2（54.8~61.6） | 55.1（50.5~59.6） | 49.7（45.1~54.3） | 47.7（44.4~51.1） | 45（39.6~50.5） | -2.4 | <0.001 |
| East | 56.6（53.4~59.8） | 51.2（47.7~54.6） | 50.5（48.3~52.7） | 49.1（46.5~51.8） | 45.9（43.4~48.4） | -1.7 | ＜0.001 |
| Middle | 63.3（58.2~68.4） | 56.3（51.6~60.9） | 58.5（54.6~62.3） | 56.5（53.4~59.5） | 56.9（53.2~60.5） | -0.8 | 0.13 |
| South | 51.8（48.5~55.0） | 52.2（49.3~55.1） | 49.1（44.7~53.4） | 53.4（48.6~58.2） | 48.7（41.8~55.5） | -0.4 | 0.65 |
| Southwest | 67.7（61.2~74.2） | 59.6（54.4~64.9） | 58.5（53.9~63.1） | 62.8（59.7~65.8） | 60.6（56.5~64.6） | -0.7 | 0.16 |
| Northwest | 52.1（41.0~63.2） | 50.6（39.4~61.7） | 52.6（47.3~58.0） | 51.3（46.2~56.4） | 53.3（48.9~57.7） | 0.2 | 0.85 |
| **Education** |  |  |  |  |  |  |  |
| No formal/primary school | 63.8（61.1~66.6） | 58.5（56.1~60.9） | 57.9（56.1~59.6） | 58.8（57.0~60.7） | 57.5（55.6~59.4） | -0.8 | 0.005 |
| Secondary school | 60.5（58.0~62.9） | 55.9（53.7~58.2） | 56.1（54.3~57.9） | 57.9（56.3~59.5） | 55.5（53.6~57.3） | -0.5 | 0.04 |
| High school | 53.4（50.6~56.1） | 51.6（49.4~53.8） | 48.7（46.4~51.0） | 50.8（48.2~53.4） | 51.1（48.2~54.0） | -0.4 | 0.29 |
| College /university | 40.4（36.6~44.2） | 41.3（38.4~44.2） | 38.8（35.7~41.8） | 36.4（33.9~39.0） | 32.9（29.7~36.0） | -1.9 | <0.001 |
| **Occupation** |  |  |  |  |  |  |  |
| Agriculture | 62.9（60.6~65.1） | 57.2（54.7~59.7） | 57.0（55.3~58.7） | 58.0（56.2~59.7） | 56.3（54.5~58.1） | -0.8 | 0.003 |
| Manufacture | 65.4（62~68.9） | 58.6（54.4~62.8） | 57.6（54.5~60.7） | 56.9（54.1~59.7） | 55.9（52.8~59.0） | -1.2 | ＜0.001 |
| Service provider | 59.2（54.0~64.4） | 55.4（51.8~58.9） | 53.9（50.5~57.2） | 53.0（50.1~56.0） | 46.4（42.7~50.1） | -2.0 | <0.001 |
| Managers/professionals | 53.0（49.0~57.1） | 49.1（46.6~51.6） | 45.1（42.5~47.7） | 46.5（44.1~49） | 42.6（39.9~45.3） | -1.8 | <0.001 |
| Others | 59.7（55.9~63.6） | 58.4（55.7~61.1） | 55.6（52.7~58.4） | 56.7（54.4~59.1） | 52.3（48.9~55.7） | -1.1 | 0.004 |
| Unemployed/students | 37.7（32.3~43.2） | 42.2（38.7~45.8） | 42.4（38.4~46.3） | 44.2（41.4~47.0） | 47.7（43.3~52.2） | 1.9 | 0.006 |
| Retired | 47.0（43.3~50.7） | 44.4（41.4~47.3） | 42.1（38.9~45.4） | 44.1（41.9~46.2） | 45.6（42.4~48.8） | -0.3 | 0.51 |

CI, confidence interval. *Since 2010.

# **Table C. The prevalence of regular smoking among men in China, 2007-2018. Values are weighted percentages (95% CI).**

|  | **2007** | **2010** | **2013** | **2015** | **2018** | **Annual rate of change** | ***p* for trend** |
| --- | --- | --- | --- | --- | --- | --- | --- |
| **Overall** | 50.4（48.0~52.8） | 47.6（45.8~49.5） | 47.4（45.9~49.0） | 47.6（46.1~49.1） | 45.6（43.9~47.3） | -0.8 | 0.008 |
| **Age, y** |  |  |  |  |  |  |  |
| 18-19 | 22.8（13.8~31.7） | 28.0（23.5~32.5） | 22.3（17.3~27.3） | 32.6（26.6~38.6） | 24.9（17.7~32.2） | 1.1 | 0.56 |
| 20-24 | 34.7（29.5~39.9） | 38.0（34.1~41.9） | 41.0（37.2~44.9） | 43.1（39.4~46.7） | 39.7（34.5~44.9） | 1.5 | 0.10 |
| 25-29 | 44.8（40.1~49.5） | 42.4（39.3~45.5） | 46.8（43.6~49.9） | 42.3（39.0~45.6） | 43.8（39.5~48.1） | -0.1 | 0.74 |
| 30-39 | 53.3（50.7~56.0） | 50.1（48.0~52.2） | 46.9（44.9~48.8） | 47.2（45.3~49.1） | 45.3（42.8~47.7） | -1.4 | <0.001 |
| 40-49 | 59.5（56.7~62.2） | 53.5（51.6~55.4） | 51.9（50.3~53.5） | 50.8（49.0~52.6） | 48.3（46.6~50.0） | -1.7 | <0.001 |
| 50-59 | 57.2（54.8~59.6） | 53.4（51.4~55.3） | 53.4（51.9~54.9） | 53.8（52.1~55.6） | 52.0（50.4~53.5） | -0.7 | 0.006 |
| 60-69 | 48.8（45.7~51.9） | 45.5（43.1~47.9） | 46.5（44.7~48.3） | 46.2（44.6~47.8） | 46.1（44.4~47.7） | -0.4 | 0.28 |
| **Birth cohort** |  |  |  |  |  |  |  |
| 1930-40s | 51.0（47.7~54.3） | 45.1（42.6~47.5） | 44.1（41.9~46.3） | 41.5（39.5~43.6） | 40.4（37.1~43.8） | -2.0 | <0.001 |
| 1950s | 58.2（55.9~60.5） | 53.1（51.1~55.1） | 52.0（50.5~53.5） | 50.6（48.9~52.4） | 47.5（45.8~49.1） | -1.7 | <0.001 |
| 1960s | 58.2（55.9~60.4） | 53.6（51.6~55.6） | 53.2（51.7~54.8） | 53.5（51.8~55.3） | 51.2（49.7~52.8） | -1.0 | ＜0.001 |
| 1970s | 51.8（48.5~55.0） | 51.0（49.1~52.9） | 48.8（46.9~50.6） | 48.6（46.8~50.4） | 48.1（46.3~49.9） | -0.7 | 0.02 |
| 1980s | 33.8（28.9~38.8） | 40.6（37.6~43.6） | 45.8（43.0~48.7） | 45.4（43.1~47.6） | 45.0（42.4~47.5） | 2.6 | <0.001 |
| 1990s | - | 29.8（25.6~34.1） | 34.7（30.7~38.6） | 41.5（38.3~44.7） | 39.1（35.1~43.2） | 3.8* | 0.004 |
| **Areas** |  |  |  |  |  |  |  |
| Urban | 48.4（43.9~52.9） | 46.2（43.5~49） | 42.7（40.4~45.1） | 42.4（40.3~44.4） | 40.8（38.2~43.4） | -1.6 | ＜0.001 |
| Rural | 51.5（48.8~54.3） | 48.5（46.0~50.9） | 50.1（48.2~52.0） | 51.3（49.6~53.0） | 49.6（47.6~51.6） | -0.1 | 0.83 |
| **Region** |  |  |  |  |  |  |  |
| North | 47.1（41.8~52.3） | 47.8（45.0~50.7） | 46.4（42.7~50.0） | 48.2（44.8~51.5） | 45.4（41.7~49.2） | -0.3 | 0.74 |
| Northeast | 53.7（50.2~57.1） | 51.1（46.6~55.6） | 46.1（41.6~50.5） | 44.5（41.3~47.7） | 41.9（36.6~47.1） | -2.3 | ＜0.001 |
| East | 48.0（45.0~51） | 44.9（41.6~48.3） | 44.8（42.7~47.0） | 43.6（40.9~46.4） | 40.6（38.2~43.0） | -1.3 | 0.005 |
| Middle | 52.4（48.2~56.6） | 47.5（42.5~52.5） | 52.0（47.2~56.7） | 49.1（45.7~52.5） | 49.7（46.3~53.2） | -0.3 | 0.63 |
| South | 42.4（37.4~47.5） | 45.0（41.6~48.4） | 42.3（37.4~47.3） | 46.5（41.7~51.3） | 42.5（35.3~49.7） | 0.1 | 0.89 |
| Southwest | 61.7（54.9~68.6） | 53.8（48.6~58.9） | 53.7（49.2~58.1） | 57.8（54.4~61.1） | 55.4（51.0~59.8） | -0.6 | 0.29 |
| Northwest | 43.3（33.1~53.4） | 44.9（34.4~55.3） | 46.9（41.5~52.4） | 45.6（40.9~50.2） | 48.5（44.2~52.7） | 0.9 | 0.51 |
| **Education** |  |  |  |  |  |  |  |
| No formal/primary school | 56.1（52.9~59.3） | 53.3（50.8~55.8） | 53.1（51.2~55.0） | 53.9（51.9~55.8） | 53.2（51.2~55.2） | -0.4 | 0.24 |
| Secondary school | 52.9（50.3~55.5） | 49.9（47.7~52.1） | 50.5（48.6~52.4） | 52.4（50.8~54） | 50.6（48.8~52.4） | -0.2 | 0.61 |
| High school | 44.8（42.4~47.2） | 44.2（42.0~46.3） | 42.9（40.7~45.1） | 45.0（42.6~47.4） | 45.0（42.0~48） | 0.1 | 0.79 |
| College /university | 30.8（27.3~34.3） | 33.1（30.5~35.8） | 30.9（27.5~34.4） | 29.2（26.8~31.6） | 26.8（24.0~29.7） | -1.4^†^ | 0.006 |
| **Occupation** |  |  |  |  |  |  |  |
| Agriculture | 55.3（52.6~57.9） | 51.5（48.9~54） | 51.9（50~53.7） | 53.0（51.0~54.9） | 51.4（49.7~53.2） | -0.5 | 0.16 |
| Manufacture | 55.9（52.2~59.7） | 52.5（48.4~56.5） | 51.1（48.1~54.2） | 50.3（47.4~53.2） | 50.6（47.4~53.9） | -0.9 | 0.03 |
| Service provider | 47.7（42.7~52.7） | 48.0（44.6~51.3） | 46.0（42.6~49.5） | 46.2（43.2~49.3） | 40.1（36.3~43.9） | -1.4 | 0.008 |
| Managers/professionals | 44.5（40.5~48.5） | 42.1（39.5~44.7） | 38.8（35.3~42.3） | 40.6（38.2~42.9） | 36.1（33.6~38.7） | -1.7 | ＜0.001 |
| Others | 52.5（48.5~56.6） | 50.9（48.2~53.6） | 49.4（46.6~52.2） | 50.2（47.9~52.5） | 47.8（44.5~51.1） | -0.8 | 0.08 |
| Unemployed/students | 31.1（26.3~35.8） | 34.6（30.6~38.6） | 37.8（34.1~41.4） | 38.5（35.7~41.2） | 43.1（38.6~47.6） | 2.9 | ＜0.001 |
| Retired | 41.7（38.2~45.2） | 40.8（38.1~43.6） | 38.8（35.6~42.1） | 40.9（38.8~42.9） | 42.0（38.7~45.3） | 0.0 | 0.99 |

CI, confidence interval. * Since 2010.

# **Table D. The prevalence of occasional smoking among men in China, 2007-2018. Values are weighted percentages (95%CI).**

|  | **2007** | **2010** | **2013** | **2015** | **2018** | **Annual rate of change** | ***p* for trend** |
| --- | --- | --- | --- | --- | --- | --- | --- |
| **Overall** | 8.0（7.1~8.9） | 6.4（5.9~6.9） | 5.7（5.2~6.2） | 5.7（5.3~6.2） | 5.2（4.8~5.7） | -3.6^*^ | <0.001 |
| **Age, y** |  |  |  |  |  |  |  |
| 18-19 | 10.8（6.7~15.0） | 8.3（6.6~10.0） | 6.7（3.2~10.2） | 7.8（5.1~10.5） | 6.7（2.7~10.7） | -3.8 | 0.11 |
| 20-24 | 8.7（6.6~10.7） | 8.6（7.1~10.1） | 6.8（5.3~8.2） | 7.0（5.4~8.7） | 5.5（3.8~7.2） | -4.1 | 0.02 |
| 25-29 | 12.1（9.1~15.2） | 6.7（5.6~7.9） | 8.0（6.2~9.8） | 7.0（5.7~8.3） | 6.8（5.3~8.2） | -4.1 | 0.004 |
| 30-39 | 9.2（7.6~10.9） | 6.5（5.7~7.2） | 5.9（5.2~6.7） | 5.7（5.0~6.4） | 5.6（4.7~6.4） | -4.2 | ＜0.001 |
| 40-49 | 6.7（5.7~7.8） | 5.9（5.3~6.5） | 5.2（4.6~5.7） | 5.5（4.8~6.3） | 5.2（4.5~5.9） | -2.2 | 0.02 |
| 50-59 | 6.1（5.3~6.9） | 5.1（4.5~5.7） | 4.5（4.0~5.0） | 4.6（4.0~5.3） | 4.1（3.7~4.5） | -3.3 | <0.001 |
| 60-69 | 5.2（4.2~6.2） | 5.3（4.4~6.2） | 3.9（3.4~4.4） | 4.3（3.8~4.8） | 4.0（3.6~4.4） | -2.7 | 0.008 |
| **Birth cohort** |  |  |  |  |  |  |  |
| 1930-40s | 5.3（4.4~6.1） | 5.2（4.3~6.1） | 3.3（2.8~3.9） | 4.1（3.4~4.8） | 4.3（3.1~5.5） | -2.5 | 0.007 |
| 1950s | 6.5（5.5~7.5） | 5.1（4.4~5.7） | 4.5（4.0~5.0） | 4.7（4.0~5.4） | 3.9（3.5~4.3） | -4.1 | <0.001 |
| 1960s | 7.1（5.9~8.3） | 5.9（5.3~6.5） | 4.7（4.2~5.2） | 4.9（4.1~5.7） | 4.2（3.7~4.6） | -4.6 | <0.001 |
| 1970s | 10.3（8.5~12.1） | 6.6（5.8~7.3） | 5.8（5.1~6.6） | 5.7（5.0~6.3） | 5.3（4.5~6.0） | -5.4 | <0.001 |
| 1980s | 9.8（7.9~11.6） | 7.4（6.4~8.5） | 7.0（5.8~8.2） | 6.2（5.3~7.0） | 5.6（4.9~6.4） | -4.7 | <0.001 |
| 1990s | - | 8.4（6.9~9.9） | 7.0（5.4~8.5） | 7.1（5.7~8.5） | 6.2（5.0~7.4） | -3.5* | 0.08 |
| **Areas** |  |  |  |  |  |  |  |
| Urban | 7.3（6.3~8.4） | 6.6（5.9~7.2） | 5.9（5.1~6.8） | 5.9（5.3~6.5） | 5.5（4.9~6.2） | -2.5 | 0.004 |
| Rural | 8.4（7.1~9.7） | 6.2（5.6~6.9） | 5.5（4.9~6.1） | 5.6（5.0~6.2） | 5.0（4.4~5.5） | -4.2 | <.0001 |
| **Region** |  |  |  |  |  |  |  |
| North | 7.1（5.0~9.3） | 5.7（4.5~6.9） | 6.0（4.9~7.2） | 5.6（4.6~6.6） | 3.6（2.9~4.3） | -5.0 | 0.007 |
| Northeast | 4.5（3.6~5.4） | 4.0（3.3~4.7） | 3.7（2.9~4.5） | 3.2（2.6~3.9） | 3.2（1.9~4.4） | -3.3 | 0.06 |
| East | 8.6（7.3~9.9） | 6.2（5.5~7.0） | 5.6（5.1~6.2） | 5.5（4.7~6.3） | 5.3（4.5~6.0） | -4.0 | ＜0.001 |
| Middle | 10.9（8.8~13.0） | 8.8（7.5~10.0） | 6.5（5.0~.08） | 7.3（5.9~8.8） | 7.1（5.7~8.5） | -3.9 | 0.006 |
| South | 9.3（6.1~12.6） | 7.2（5.2~9.2） | 6.7（4.6~8.9） | 7.0（5.9~.08） | 6.2（5.0~7.3） | -3.1 | 0.07 |
| Southwest | 6.0（4.0~7.9） | 5.9（4.8~6.9） | 4.8（3.6~6.1） | 5.0（4.1~5.8） | 5.2（3.9~6.4） | -1.7 | 0.35 |
| Northwest | 8.8（7.1~10.6） | 5.7（4.6~6.9） | 5.7（4.5~6.9） | 5.7（4.5~6.9） | 4.8（3.7~6.0） | -4.5 | 0.002 |
| **Education** |  |  |  |  |  |  |  |
| No formal/primary school | 7.8（6.5~9.0） | 5.2（4.6~5.8） | 4.7（4.1~5.4） | 5.0（4.4~5.6） | 4.3（3.8~4.9） | -4.6 | <0.001 |
| Secondary school | 7.6（6.4~8.8） | 6.0（5.4~6.6） | 5.6（5.0~6.2） | 5.6（4.9~6.2） | 4.9（4.2~5.5） | -3.5 | <0.001 |
| High school | 8.5（7.1~10.0） | 7.5（6.5~8.4） | 5.8（4.9~6.7） | 5.7（4.9~6.5） | 6.2（5.1~7.3） | -3.3 | ＜0.001 |
| College /university | 9.6（6.5~12.7） | 8.1（7.0~9.2） | 7.8（6.3~9.4） | 7.2（6.0~8.5） | 6.0（4.8~7.3） | -3.8 | 0.02 |
| **Occupation** |  |  |  |  |  |  |  |
| Agriculture | 7.6（6.4~8.9） | 5.7（5.1~6.3） | 5.2（4.5~5.9） | 5.0（4.3~5.7） | 4.9（4.2~5.5） | -3.7 | ＜0.001 |
| Manufacture | 9.5（7.5~11.5） | 6.1（4.8~7.4） | 6.5（5.4~7.6） | 6.6（5.4~7.9） | 5.2（4.0~6.4） | -4.2 | 0.002 |
| Service provider | 11.5（8.5~14.5） | 7.4（6.1~8.7） | 7.8（6.0~9.6） | 6.8（5.6~8.1） | 6.3（4.9~7.7） | -4.7 | 0.003 |
| Managers/professionals | 8.5（6.5~10.6） | 7.0（6.0~7.9） | 6.3（4.5~8.0） | 5.9（5.1~6.7） | 6.5（5.1~7.8） | -2.6 | 0.07 |
| Others | 7.2（5.9~8.6） | 7.5（6.3~8.7） | 6.2（5.1~7.2） | 6.5（5.5~7.5） | 4.5（3.7~5.4） | -3.9 | ＜0.001 |
| Unemployed/students | 6.7（4.5~8.8） | 7.6（6.3~8.9） | 4.6（3.1~6.1） | 5.7（4.3~7.1） | 4.6（3.6~5.6） | -3.9 | 0.01 |
| Retired | 5.3（4.0~6.7） | 3.5（2.7~4.4） | 3.3（2.6~4.0） | 3.2（2.6~3.9） | 3.6（2.9~4.3） | -3.2 | 0.02 |

CI, confidence interval. *Since 2010.

# **Table E. The prevalence of former smoking among men in China, 2007-2018. Values are weighted percentages (95% CI).**

|  | **2007** | **2010** | **2013** | **2015** | **2018** | **Annual rate of change** | ***p* for trend** |
| --- | --- | --- | --- | --- | --- | --- | --- |
| **Overall** | 7.5（6.6~8.4） | 8.3（7.7~9.0） | 7.7（7.2~8.3） | 8.7（8.2~9.3） | 7.8（7.3~8.3） | 0.4 | 0.41 |
| **Age, y** |  |  |  |  |  |  |  |
| 18-19 | 1.5（0.0~3.0） | 2.4（1.2~3.7） | 0.9（0.2~1.6） | 3.0（1.3~4.6） | 3.1（0.0~6.1） | 5.8 | 0.34 |
| 20-24 | 3.4（1.9~4.9） | 3.7（2.8~4.6） | 2.5（1.7~3.2） | 2.9（1.9~3.9） | 2.8（1.6~4.0） | -2.4 | 0.37 |
| 25-29 | 3.0（1.7~4.4） | 4.2（3.3~5.1） | 3.1（2.3~4.0） | 6.2（3.5~8.9） | 2.7（1.8~3.6） | 0.4 | 0.56 |
| 30-39 | 5.3（4.4~6.2） | 6.0（5.3~6.6） | 5.2（4.5~5.9） | 5.4（4.7~6.0） | 5.2（4.4~6.0） | -0.5 | 0.60 |
| 40-49 | 7.7（6.6~8.9） | 8.6（7.8~9.4） | 8.3（7.5~9.1） | 8.6（7.8~9.4） | 7.8（7.0~8.5） | 0.1 | 0.93 |
| 50-59 | 11.2（9.4~13.1） | 12.3（11.3~13.3） | 12.7（11.8~13.5） | 13.5（12.5~14.5） | 12.7（11.8~13.5） | 1.3 | 0.10 |
| 60-69 | 17.9（14.7~21.1） | 18.8（17.0~20.7） | 17.8（16.3~19.2） | 20.7（19.4~22.0） | 19.5（18.2~20.7） | 0.9 | 0.23 |
| **Birth cohort** |  |  |  |  |  |  |  |
| 1930-40s | 16.3（13.5~19.1） | 19.2（17.4~21.1） | 19.2（17.4~21.0） | 23.4（21.5~25.2） | 20.9（17.8~23.9） | 2.6 | 0.004 |
| 1950s | 10.1（8.4~11.8） | 12.8（11.7~13.8） | 14.7（13.7~15.6） | 17.6（16.5~18.8） | 18.7（17.5~20.0） | 5.9 | <0.001 |
| 1960s | 7.4（6.3~8.4） | 8.9（8.1~9.8） | 9.7（8.9~10.5） | 10.5（9.8~11.2） | 12.1（11.3~13.0） | 4.4 | <0.001 |
| 1970s | 4.6（3.6~5.6） | 6.0（5.3~6.7） | 6.3（5.5~7.0） | 7.2（6.5~8.0） | 7.5（6.7~8.3） | 4.4 | <0.001 |
| 1980s | 2.8（1.7~3.8） | 3.9（3.2~4.6） | 3.4（2.8~4.0） | 4.8（4.1~5.5） | 4.5（3.8~5.2） | 4.3 | 0.007 |
| 1990s | - | 3.2（2.0~4.3） | 1.9（1.2~2.7） | 4.2（2.5~5.8） | 3.0（2.1~3.8） | 1.5* | 0.51 |
| **Areas** |  |  |  |  |  |  |  |
| Urban | 7.6（6.3~8.9） | 8.2（7.2~9.1） | 7.1（6.5~7.8） | 7.9（7.3~8.6） | 7.4（6.7~8.0） | -0.4 | 0.69 |
| Rural | 7.4（6.2~8.6） | 8.4（7.6~9.3） | 8.1（7.4~8.8） | 9.3（8.5~10.1） | 8.2（7.5~9.0） | 1.1 | 0.15 |
| **Region** |  |  |  |  |  |  |  |
| North | 5.3（3.5~7.1） | 5.3（3.5~7.1） | 6.0（4.8~7.2） | 7.5（6.5~8.4） | 7.5（5.9~9.2） | 3.8 | 0.15 |
| Northeast | 7.0（5.0~8.9） | 7.0（5.0~8.9） | 7.4（5.6~9.3） | 7.2（6.0~8.5） | 6.7（5.0~8.5） | -0.2 | 0.96 |
| East | 8.9（7.0~10.7） | 8.9（7.0~10.7） | 8.3（7.3~9.3） | 9.5（8.5~10.4） | 8.3（7.5~9.1） | -0.3 | 0.76 |
| Middle | 9.0（6.4~11.6） | 9.0（6.4~11.6） | 9.1（7.5~10.6） | 10.1（8.2~11.9） | 8.6（7.2~10.0） | 0.0 | 0.86 |
| South | 7.8（6.1~9.5） | 7.8（6.1~9.5） | 7.1（6.0~8.2） | 7.9（5.9~10.0） | 7.0（5.8~8.3） | -0.8 | 0.91 |
| Southwest | 5.8（3.8~7.8） | 5.8（3.8~7.8） | 7.6（6.3~8.8） | 8.8（7.4~10.2） | 8.0（6.7~9.3） | 3.9 | 0.20 |
| Northwest | 7.5（5.9~9.1） | 7.5（5.9~9.1） | 7.6（6.4~8.8） | 8.3（7.3~9.2） | 7.3（5.7~9.0） | -0.1 | 0.35 |
| **Education** |  |  |  |  |  |  |  |
| No formal/primary school | 9.1（7.6~10.6） | 11.3（10.3~12.4） | 10.0（9.2~10.8） | 11.4（10.6~12.3） | 10.3（9.6~11） | 1.0 | 0.19 |
| Secondary school | 6.8（5.8~7.9） | 7.7（6.9~8.5） | 7.3（6.7~7.9） | 8.4（7.7~9.1） | 7.9（7.2~8.6） | 1.4 | 0.06 |
| High school | 7.1（5.7~8.4） | 7.0（6.2~7.7） | 7.2（6.4~8.0） | 8.7（7.4~10） | 7.1（6.2~8.0） | 0.7 | 0.43 |
| College /university | 5.9（4.5~7.4） | 6.3（5.3~7.4） | 5.0（3.8~6.1） | 5.3（4.4~6.1） | 5.6（4.7~6.4） | -1.0 | 0.40 |
| **Occupation** |  |  |  |  |  |  |  |
| Agriculture | 8.1（6.7~9.5） | 8.9（8.0~9.8） | 8.7（7.9~9.4） | 9.5（8.6~10.3） | 9.1（8.3~9.9） | 1.1 | 0.19 |
| Manufacture | 5.6（4.3~6.9） | 5.8（4.5~7.2） | 7.2（6.1~8.4） | 7.8（6.4~9.2） | 6.3（5.2~7.4） | 2.0 | 0.06 |
| Service provider | 6.5（4.6~8.4） | 7.7（6.5~8.9） | 6.1（4.9~7.2） | 7.0（5.6~8.4） | 7.0（5.8~8.2） | 0.2 | 0.90 |
| Managers/professionals | 6.0（4.8~7.2） | 6.8（5.9~7.7） | 6.8（5.9~7.7） | 7.0（5.8~8.1） | 6.1（5.2~7.0） | 0.3 | 0.97 |
| Others | 6.0（4.3~7.7） | 7.2（6.1~8.3） | 5.9（5.0~6.9） | 7.2（6.2~8.2） | 6.0（5.2~6.9） | 0.0 | 0.97 |
| Unemployed/students | 5.3（3.7~6.8） | 7.5（6.2~8.9） | 5.4（4.5~6.2） | 10.1（7.7~12.5） | 7.7（6.4~8.9） | 3.7 | 0.01 |
| Retired | 21.3（18.2~24.3） | 22.2（19.5~24.9） | 20.6（17.9~23.2） | 20.9（19.0~22.8） | 19.6（16.7~22.6） | -0.8 | 0.46 |

CI, confidence interval. *Since 2010.

# **Table F. The prevalence of current smoking among men and women living in poor and non-poor rural areas in China, 2007-2018. Values are weighted percentages (95%CI).**

|  | **Men** | | | | | | |  | **Women** | | | | | | |
| --- | --- | --- | --- | --- | --- | --- | --- | --- | --- | --- | --- | --- | --- | --- | --- |
|  | **2007** | **2010** | **2013** | **2015** | **2018** | **Annual rate of change** | ***p* for trend** |  | **2007** | **2010** | **2013** | **2015** | **2018** | **Annual rate of change** | ***p* for trend** |
| **Poor rural areas*** | 60.6  (55.7~65.6) | 56.5  (50.5~62.5) | 57.5  (54.1~60.9) | 56.6  (52.9~60.2) | 56.2  (52.7~59.7) | -0.6 | 0.3334 |  | 2.7  (1.0~4.4) | 2.6  (1.5~3.8) | 1.7  (1.0~2.4) | 1.9  (0.8~3.0) | 2.1  (1.1~3.2) | -3.1 | 0.45 |
| 18-19 | 40.5  (15.6~65.3) | 45.4  (32.1~58.8) | 31.4  (19.7~43.1) | 44.6  (33.1~56.0) | 29.9  (13.3~46.6) | -2.4 | 0.5390 |  | 0.1  (0.0~0.3) | 1.2  (0.0~2.9) | 3.1  (0.0~8.9) | 0.0  (0.0~0.0) | 4.5  (0.0~12.1) | 38.1 | 0.17 |
| 20-24 | 51.2  (41.0~61.4) | 48.5  (36.7~60.4) | 56.0  (47.2~64.8) | 57.1  (48.6~65.6) | 63.7  (49.3~78.0) | 2.2 | 0.1383 |  | 3.0  (0.0~6.6) | 0.5  (0.0~1.1) | 0.6  (0.0~1.3) | 1.8  (0.0~4.2) | 0.6  (0.0~1.5) | -7.7 | 0.26 |
| 25-29 | 59.3  (49.5~69.0) | 51.5  (43.5~59.6) | 61.1  (53.3~69.0) | 56.2  (48.9~63.6) | 56.4  (46.9~66.0) | 0.0 | 0.9773 |  | 1.7  (0.1~3.3) | 0.6  (0.0~1.6) | 1.1  (0.2~2.1) | 0.5  (0.0~1.0) | 1.1  (0.0~2.3) | -3.6 | 0.57 |
| 30-39 | 66.5  (61.5~71.5) | 60.2  (55.5~64.9) | 56.5  (52.2~60.7) | 56.6  (51.4~61.9) | 58.0  (53.9~62.1) | -1.2 | 0.0251 |  | 2.5  (0.7~4.3) | 2.3  (0.6~3.9) | 1.5  (0.7~2.2) | 2.4  (0.7~4.1) | 2.1  (0.7~3.5) | -1.4 | 0.80 |
| 40-49 | 65.5  (60.8~70.3) | 60.6  (54.4~66.9) | 61.5  (57.4~65.5) | 57.1  (51.7~62.5) | 55.2  (51.0~59.3) | -1.5 | 0.0058 |  | 1.9  (0.5~3.2) | 2.9  (1.2~4.5) | 1.2  (0.6~1.9) | 1.9  (0.9~2.9) | 2.7  (1.1~4.2) | 0.8 | 0.84 |
| 50-59 | 65.0  (61.2~68.9) | 60.4  (54.7~66.0) | 57.9  (54.3~61.4) | 59.5  (55.9~63.1) | 56.9  (51.7~62.0) | -1.1 | 0.0609 |  | 3.6  (0.5~6.7) | 3.9  (2.0~5.8) | 2.5  (1.3~3.6) | 1.6  (0.8~2.4) | 2.4  (1.2~3.7) | -5.9 | 0.20 |
| 60-69 | 55.0  (47.9~62.2) | 53.8  (44.8~62.7) | 55.7  (51.9~59.5) | 54.0  (49.4~58.5) | 53.4  (48.7~58.0) | -0.2 | 0.8235 |  | 5.2  (0.3~10.1) | 5.8  (3.0~8.5) | 3.3  (1.5~5.0) | 3.8  (1.8~5.8) | 3.3  (1.2~5.3) | -4.9 | 0.29 |
| **Non-poor rural areas** | 59.7  (57.0~62.4) | 54.0  (51.9~56.2) | 55.0  (52.9~57.1) | 56.8  (55.1~58.5) | 53.9  (51.5~56.3) | -0.6 | 0.0626 |  | 2.3  (1.1~3.5) | 1.9  (1.0~2.7) | 1.7  (1.3~2.2) | 2.1  (1.5~2.8) | 1.8  (1.1~2.4) | -1.6 | 0.67 |
| 18-19 | 35.1  (23.3~46.8) | 35.9  (29.1~42.7) | 33.7  (25.8~41.6) | 49.8  (39.4~60.2) | 43.3  (27.7~58.8) | 2.6 | 0.1741 |  | 0.8  (0.0~2.2) | 0.6  (0.0~1.4) | 0.0  (0.0~0.0) | 0.0  (0.0~0.0) | 0.0  (0.0~0.0) | -9.1 | ＜0.001 |
| 20-24 | 44.2  (36.2~52.2) | 45.9  (39.8~52.1) | 49.4  (43.5~55.2) | 59.5  (54.0~65.0) | 47.0  (38.1~55.8) | 1.3 | 0.1359 |  | 2.3  (0.0~5.5) | 1.2  (0.3~2.0) | 0.1  (0.0~0.3) | 0.3  (0.0~0.6) | 0.7  (0.1~1.4) | -13.8 | 0.14 |
| 25-29 | 61.1  (55.9~66.4) | 51.1  (46.3~55.9) | 58.7  (54.4~63.0) | 53.6  (48.6~58.7) | 54.0  (47.9~60.1) | -0.7 | 0.1850 |  | 0.2  (0.0~0.4) | 1.3  (0.5~2.2) | 0.7  (0.3~1.2) | 0.7  (0.3~1.1) | 0.9  (0.2~1.5) | 9.7 | 0.35 |
| 30-39 | 60.4  (56.8~64.0) | 55.9  (53.3~58.5) | 55.7  (53.4~58.0) | 56.8  (54.2~59.4) | 56.0  (52.6~59.5) | -0.5 | 0.1807 |  | 0.9  (0.2~1.6) | 1.2  (0.4~1.9) | 1.0  (0.6~1.4) | 1.6  (0.9~2.4) | 1.1  (0.6~1.5) | 2.4 | 0.57 |
| 40-49 | 66.8  (63.4~70.2) | 58.3  (55.7~60.9) | 57.9  (55.9~60.0) | 57.6  (54.9~60.3) | 55.7  (53.0~58.3) | -1.4 | 0.0002 |  | 2.8  (0.5~5.0) | 1.6  (0.7~2.4) | 1.6  (0.9~2.2) | 2.0  (1.3~2.7) | 1.6  (0.9~2.3) | -3.4 | 0.49 |
| 50-59 | 64.3  (60.9~67.6) | 58.1  (55.5~60.7) | 59.2  (57.1~61.3) | 59.6  (57.6~61.7) | 56.8  (54.9~58.6) | -0.8 | 0.0079 |  | 3.8  (1.5~6.0) | 2.8  (1.4~4.2) | 3.5  (2.5~4.5) | 4.0  (2.6~5.3) | 2.9  (1.8~4.0) | -0.8 | 0.87 |
| 60-69 | 57.5  (54.0~61.0) | 51.7  (48.5~54.9) | 51.1  (48.6~53.6) | 52.0  (49.7~54.3) | 51.0  (48.4~53.6) | -0.9 | 0.0417 |  | 4.1  (1.7~6.5) | 4.1  (2.1~6.1) | 4.1  (2.8~5.4) | 4.1  (2.6~5.7) | 4.1  (2.6~5.6) | 0.0 | 0.99 |

CI, confidence interval. * Poor rural areas refer to areas where GDP and per capita income are relatively low and were classified as poverty counties by the Office of Poverty Alleviation of the State Council. By then end of 2020~ the Chinese government announced that all poverty counties were lifted out of poverty.

# **Table G. The prevalence of current smoking among men and women by area, age and year of birth in China, 2007-2018. Values are weighted percentages (95%CI).**

|  | **Men** | | | | | | |  | **Women** | | | | | | |
| --- | --- | --- | --- | --- | --- | --- | --- | --- | --- | --- | --- | --- | --- | --- | --- |
|  | **2007** | **2010** | **2013** | **2015** | **2018** | **Annual rate of change** | ***p* for trend** |  | **2007** | **2010** | **2013** | **2015** | **2018** | **Annual rate of change** | ***p* for trend** |
| **Urban areas** |  |  |  |  |  |  |  |  |  |  |  |  |  |  |  |
| **Age~ y** |  |  |  |  |  |  |  |  |  |  |  |  |  |  |  |
| 18-19 | 25.3  (13.8~36.7) | 32.4  (27.0~37.8) | 22.4  (14.6~30.3) | 29.2  (20.7~37.6) | 25.8  (15.2~36.5) | -0.3 | 0.86 |  | 0.0  (0.0~0.0) | 1.3  (0.0~3.2) | 1.1  (0.1~2.2) | 0.5  (0.0~1.5) | 6.9  (0.0~18.2) | 18.9 | 0.11 |
| 20-24 | 40.0  (30.7~49.2) | 46.0  (40.7~51.3) | 42.8  (37.2~48.4) | 38.8  (34.5~43.2) | 38.6  (32.8~44.3) | -0.8 | 0.34 |  | 1.6  (0.0~3.3) | 1.1  (0.3~1.8) | 2.1  (0.6~3.6) | 0.2  (0.0~0.5) | 1.6  (0.3~2.9) | -5.1 | 0.79 |
| 25-29 | 52.6  (44.9~60.3) | 46.6  (41.1~52.1) | 47.9  (43.6~52.3) | 42.8  (37.1~48.4) | 46.4  (40.2~52.5) | -1.2 | 0.13 |  | 1.1  (0.0~2.3) | 1.8  (1.0~2.5) | 1.2  (0.5~1.9) | 0.6  (0.2~1.0) | .1.2  (0.5~1.9) | -3.0 | 0.38 |
| 30-39 | 63.2  (59.5~66.9) | 55.7  (51.6~59.8) | 47.6  (45.1~50.0) | 47.8  (44.1~51.4) | 45.1  (41.5~48.7) | -2.2 | <0.001 |  | 2.2  (1.2~3.2) | 2.3  (1.3~3.2) | 2.1  (1.3~3.0) | 1.4  (0.9~1.9) | 1.1  (0.7~1.6) | -6.6 | 0.003 |
| 40-49 | 65.5  (61.6~69.3) | 60.4  (57.5~63.3) | 54.1  (51.3~56.9) | 54.7  (51.7~57.6) | 50.6  (47.7~53.4) | -2.3 | <0.001 |  | 2.2  (1.5~2.8) | 2.6  (1.8~3.4) | 2.4  (1.9~3.0) | 2.0  (1.4~2.6) | .2.1  (1.5~2.8) | -1.2 | 0.57 |
| 50-59 | 61.5  (57.1~65.8) | 57.9  (54.9~61.0) | 55.9  (53.1~58.7) | 56.7  (54.3~59.0) | 54.6  (52.0~57.2) | -1.0 | 0.004 |  | 2.4  (1.5~3.2) | 3.2  (2.2~4.3) | 3.3  (2.4~4.2) | 2.9  (1.9~3.9) | .2.3  (1.7~2.9) | -0.5 | 0.72 |
| 60-69 | 47.2  (43.0~51.4) | 47.2  (43.5~50.9) | 46.7  (43.8~49.5) | 47.1  (44.5~49.7) | 48.2  (45.6~50.8) | 0.1 | 0.76 |  | 3.9  (2.6~5.2) | 3.9  (2.2~5.5) | 3.6  (2.5~4.6) | 2.9  (2.1~3.7) | 2.2  (1.7~2.8) | -5.1 | 0.03 |
| **Birth cohort** |  |  |  |  |  |  |  |  |  |  |  |  |  |  |  |
| 1930-40s | 49.9  (44.4~55.4) | 46.0  (42.5~49.4) | 42.7  (39.6~45.8) | 41.9  (38.6~45.2) | .42.5  (38.0~46.9) | -1.5 | 0.004 |  | 3.4  (2.3~4.4) | 4.0  (2.4~5.6) | 3.9  (2.8~5.0) | 3.7  (2.6~4.9) | 2.7  (1.5~4.0) | -1.9 | 0.75 |
| 1950s | 63.5  (59.9~67.1) | 57.6  (54.6~60.7) | 54.1  (51.6~56.7) | 53.1  (50.8~55.4) | .49.6  (47.2~52.0) | -2.1 | <0.001 |  | 2.8  (1.8~3.7) | 3.2  (2.1~4.3) | 3.2  (2.4~4.1) | 2.7  (1.9~3.4) | 2.4  (1.8~3.0) | -1.6 | 0.52 |
| 1960s | 64.7  (60.9~68.4) | 60.8  (57.8~63.8) | 55.4  (52.9~57.8) | 57.5  (55.4~59.6) | .54.3  (51.6~56.9) | -1.5 | <0.001 |  | 1.9  (1.4~2.5) | 2.7  (1.9~3.6) | 2.9  (2.2~3.6) | 2.7  (1.7~3.7) | .2.1  (1.6~2.6) | 0.9 | 0.74 |
| 1970s | 61.6  (57.2~66.0) | 56.8  (53.3~60.4) | 51.3  (48.6~54.0) | 50.6  (47.8~53.4) | 49.9  (46.9~53.0) | -2.0 | <0.001 |  | 2.0  (0.7~3.3) | 2.3  (1.4~3.2) | 2.1  (1.3~2.9) | 1.7  (1.2~2.2) | .2.1  (1.5~2.8) | -0.6 | 0.80 |
| 1980s | 38.9  (30.3~47.5) | 46.3  (41.7~50.8) | 45.8  (42.7~48.8) | 46.1  (42.3~49.9) | 45.4  (41.7~49.2) | 1.1 | 0.14 |  | 1.1  (0.0~2.2) | 1.5  (0.9~2.2) | 2.0  (0.9~3.1) | 1.0  (0.6~1.3) | 1.1  (0.6~1.5) | -1.1 | 0.63 |
| 1990s | - | 35.4  (30.2~40.5) | 36.5  (30.1~42.9) | 37.6  (33.6~41.6) | 38.7. (34.5~42.9) | 1.1* | 0.35 |  | - | 1.0  (0.0~2.4) | 1.4  (0.3~2.4) | 0.3  (0.1~0.6) | .2.0  (0.5~3.5) | 4.0* | 0.32 |
| **Rural areas** |  |  |  |  |  |  |  |  |  |  |  |  |  |  |  |
| **Age~ y** |  |  |  |  |  |  |  |  |  |  |  |  |  |  |  |
| 18-19 | 39.7  (26.7~52.8) | 39.2  (32.3~46.0) | 33.4  (26.7~40.1) | 47.9  (40.0~55.8) | 38.4  (26.7~50.1) | -0.3 | 0.81 |  | 0.6  (0.0~1.7) | 0.8  (0.1~1.6) | 0.9  (0.0~2.6) | 0.0  (0.0~0.0) | 1.4  (0.0~4.0) | 7.7 | 0.84 |
| 20-24 | 45.7  (39.0~52.4) | 47.0  (41.3~52.6) | 51.0  (45.9~56.2) | 59.5  (54.9~64.1) | 52.2  (43.6~60.7) | 1.8 | 0.05 |  | 1.7  (0.0~4.0) | 1.0  (0.4~1.6) | 0.2  (0.0~0.3) | 0.7  (0.0~1.4) | 0.7  (0.1~1.2) | -8.3 | 0.30 |
| 25-29 | 60.0  (55.4~64.7) | 51.0  (46.8~55.1) | 59.3  (55.4~63.2) | 54.3  (50.1~58.6) | 54.5  (49.3~59.7) | -0.5 | 0.37 |  | 0.4  (0.0~0.8) | 1.2  (0.5~1.9) | 0.8  (0.4~1.2) | 0.6  (0.3~1.0) | 1.0  (0.4~1.6) | 4.7 | 0.50 |
| 30-39 | 62.1  (59.0~65.2) | 57.1  (54.7~59.6) | 55.9  (53.8~58.0) | 57.2  (54.9~59.5) | 56.4  (53.7~59.2) | -0.7 | 0.03 |  | 1.1  (0.4~1.8) | 1.6  (0.8~2.4) | 1.2  (0.8~1.6) | 1.8  (1.1~2.5) | 1.4  (0.8~2.0) | 2.1 | 0.63 |
| 40-49 | 66.6  (63.7~69.5) | 58.8  (56.3~61.4) | 58.7  (56.8~60.6) | 57.4  (54.9~59.9) | 55.6  (53.4~57.8) | -1.4 | <0.001 |  | 2.6  (0.8~4.4) | 2.0  (1.2~2.9) | 1.5  (1.0~2.0) | 2.0  (1.4~2.6) | 1.9  (1.1~2.6) | -2.5 | 0.52 |
| 50-59 | 64.3  (61.5~67.1) | 58.7  (56.3~61.1) | 58.9  (57.0~60.7) | 59.6  (57.8~61.4) | 57.0  (55.2~58.8) | -0.9 | 0.003 |  | 3.9  (1.9~5.8) | 3.2  (2.0~4.4) | 3.3  (2.5~4.1) | 3.5  (2.4~4.6) | 2.9  (2.0~3.8) | -2.0 | 0.56 |
| 60-69 | 57.2  (54.1~60.4) | 52.5  (49.4~55.7) | 52.1  (49.9~54.3) | 52.5  (50.4~54.5) | 51.3  (49.0~53.6) | -0.8 | 0.025 |  | 4.5  (2.2~6.9) | 4.7  (3.0~6.4) | 3.9  (2.8~5.0) | 4.2  (2.8~5.5) | 4.0  (2.7~5.2) | -1.3 | 0.65 |
| **Birth cohort** |  |  |  |  |  |  |  |  |  |  |  |  |  |  |  |
| 1930-40s | 59.3  (55.8~62.7) | 52.3  (49.1~55.6) | 49.5  (46.7~52.3) | 47.9  (45.4~50.5) | 46.2  (41.4~51.1) | -2.1 | <0.001 |  | 4.5  (2.3~6.8) | 4.8  (3.2~6.5) | 3.9  (2.7~5.1) | 4.3  (2.9~5.7) | 4.4  (2.5~6.4) | -0.6 | 0.77 |
| 1950s | 65.4  (62.7~68.2) | 58.4  (55.9~60.9) | 57.7  (55.9~59.6) | 56.6  (54.6~58.7) | 52.5  (50.3~54.8) | -1.7 | <0.001 |  | 3.6  (1.4~5.7) | 3.3  (2.0~4.6) | 3.8  (2.8~4.7) | 4.1  (2.8~5.3) | 4.0  (2.7~5.3) | 1.6 | 0.65 |
| 1960s | 65.6  (63.0~68.2) | 58.7  (56.1~61.4) | 59.3  (57.4~61.2) | 59.1  (57.1~61.0) | 56.1  (54.2~58.0) | -1.2 | <0.001 |  | 2.3  (0.8~3.8) | 2.1  (1.3~3.0) | 2.0  (1.4~2.6) | 2.8  (2.0~3.6) | 2.6  (1.7~3.5) | 1.8 | 0.62 |
| 1970s | 62.3  (59.3~65.4) | 58.0  (55.6~60.3) | 56.4  (54.4~58.4) | 56.9  (54.5~59.3) | 55.9  (53.7~58.1) | -0.9 | 0.007 |  | 0.8  (0.4~1.3) | 1.6  (0.8~2.4) | 1.3  (0.9~1.7) | 1.9  (1.3~2.5) | 2.0  (1.2~2.8) | 7.7 | 0.06 |
| 1980s | 46.8  (40.3~53.3) | 49.3  (44.9~53.6) | 57.3  (53.9~60.7) | 56.2  (53.7~58.7) | 55.6  (52.5~58.7) | 1.8 | 0.007 |  | 1.1  (0.0~2.4) | 1.1  (0.5~1.7) | 0.8  (0.5~1.1) | 1.1  (0.6~1.6) | 1.1  (0.7~1.6) | -1.7 | 0.99 |
| 1990s | - | 40.2  (34.0~46.4) | 45.1  (40.3~50.0) | 57.2  (53.1~61.3) | 52.1  (45.7~58.5) | 3.8* | 0.007 |  | - | 0.8  (0.2~1.5) | 0.3  (0.0~0.8) | 0.6  (0.0~1.1) | 0.9  (0.4~1.3) | 3.5* | 0.46 |

CI, confidence interval. * Since 2010.

# **Table H. The prevalence of current smoking among women in China, 2007-2018. Values are weighted percentages (95%CI).**

|  | **2007** | **2010** | **2013** | **2015** | **2018** | **Annual rate of change** | ***p* for trend** |
| --- | --- | --- | --- | --- | --- | --- | --- |
| **Overall** | 2.2（1.5~2.9） | 2.2（1.7~2.8） | 2.0（1.6~2.3） | 1.9（1.5~2.3） | 1.9（1.5~2.3） | -1.6 | 0.37 |
| **Age~ y** |  |  |  |  |  |  |  |
| 18-19 | 0.4（0.0~1.1） | 1.0（0.1~1.9） | 1.0（0.0~2.1） | 0.2（0.0~0.7） | 4.3（0.0~10.6） | 13.3 | 0.15 |
| 20-24 | 1.7（0.1~3.2） | 1.0（0.5~1.5） | 1.0（0.3~1.6） | 0.5（0.1~0.9） | 1.2（0.5~1.9） | -4.9 | 0.41 |
| 25-29 | 0.7（0.1~1.2） | 1.4（0.9~1.9） | 1.0（0.6~1.4） | 0.6（0.4~0.9） | 1.1（0.6~1.5） | 0.6 | 0.99 |
| 30-39 | 1.5（0.9~2.1） | 1.8（1.2~2.5） | 1.5（1.1~1.9） | 1.6（1.1~2.0） | 1.3（0.9~1.6） | -1.5 | 0.40 |
| 40-49 | 2.5（1.3~3.7） | 2.2（1.6~2.8） | 1.8（1.4~2.2） | 2.0（1.6~2.5） | 2.0（1.5~2.5） | -2.1 | 0.46 |
| 50-59 | 3.3（2.1~4.6） | 3.2（2.4~4.1） | 3.3（2.7~3.9） | 3.3（2.5~4.0） | 2.6（2.0~3.2） | -1.6 | 0.47 |
| 60-69 | 4.3（2.7~5.9） | 4.4（3.2~5.7） | 3.8（3.0~4.6） | 3.7（2.8~4.5） | 3.2（2.5~4.0） | -2.8 | 0.22 |
| **Birth cohort** |  |  |  |  |  |  |  |
| 1930-40s | 4.1（2.6~5.6） | 4.6（3.3~5.8） | 3.9（3.0~4.8） | 4.1（3.1~5.0） | 3.7（2.5~5.0） | -1.2 | 0.84 |
| 1950s | 3.3（1.9~4.7） | 3.2（2.3~4.2） | 3.6（2.9~4.3） | 3.5（2.7~4.3） | 3.3（2.5~4.1） | 0.4 | 0.84 |
| 1960s | 2.1（1.2~3.1） | 2.3（1.7~3.0） | 2.3（1.9~2.8） | 2.7（2.1~3.4） | 2.4（1.9~2.9） | 1.5 | 0.56 |
| 1970s | 1.3（0.7~1.9） | 1.9（1.3~2.5） | 1.6（1.2~1.9） | 1.8（1.4~2.2） | 2.1（1.5~2.6） | 3.4 | 0.11 |
| 1980s | 1.1（0.2~2.0） | 1.3（0.8~1.7） | 1.3（0.8~1.7） | 1.0（0.7~1.3） | 1.1（0.8~1.4） | -0.8 | 0.85 |
| 1990s | - | 0.9（0.2~1.6） | 0.7（0.2~1.2） | 0.5（0.1~0.8） | 1.4（0.7~2.2） | 4.3^*^ | 0.19 |
| **Areas** |  |  |  |  |  |  |  |
| Urban | 2.1（1.5~2.6） | 2.4（1.8~3.0） | 2.4（1.8~2.9） | 1.6（1.2~2.0） | 1.9（1.3~2.4） | -2.0 | 0.26 |
| Rural | 2.3（1.3~3.3） | 2.2（1.4~2.9） | 1.7（1.3~2.2） | 2.1（1.5~2.6） | 1.9（1.3~2.5） | -1.7 | 0.60 |
| **Region** |  |  |  |  |  |  |  |
| North | 1.7（1.1~2.3） | 3.4（2.1~4.7） | 3.0（2.0~4.0） | 3.3（1.9~4.8） | 4.3（2.8~5.9） | 7.1 | 0.02 |
| Northeast | 9.2（5.7~12.7） | 8.5（6.5~10.5） | 6.6（5.1~8.1） | 7.0（5.0~8.9） | 7.0（4.9~9.1） | -2.8 | 0.22 |
| East | 1.0（0.6~1.5） | 0.9（0.6~1.2） | 1.1（0.7~1.4） | 0.8（0.6~1.0） | 0.6（0.4~0.7） | -4.0 | 0.04 |
| Middle | 1.4（0.5~2.3） | 1.5（0.7~2.3） | 1.5（0.9~2.1） | 1.3（0.6~1.9） | 1.1（0.6~1.6） | -2.2 | 0.61 |
| South | 0.6（0.1~1.1） | 1.0（0.5~1.5） | 1.1（0.5~1.7） | 1.4（0.6~2.2） | 1.0（0.5~1.5） | 5.2 | 0.31 |
| Southwest | 2.3（1.4~3.2） | 1.9（0.7~3.1） | 1.7（1.0~2.5） | 1.3（0.7~2.0） | 1.6（1.1~2.1） | -4.0 | 0.11 |
| Northwest | 1.1（0.4~1.8） | 1.1（0.5~1.8） | 0.8（0.4~1.2） | 0.7（0.4~1.0） | 0.5（0.3~0.8） | -7.2 | 0.11 |
| **Education** |  |  |  |  |  |  |  |
| No formal/primary school | 3.2（1.8~4.6） | 3.1（2.2~4.1） | 2.7（2.1~3.2） | 3.1（2.4~3.8） | 3.0（2.2~3.9） | -0.6 | 0.87 |
| Secondary school | 1.8（1.2~2.4） | 1.9（1.4~2.4） | 1.7（1.4~2.0） | 1.3（1.0~1.6） | 1.5（1.2~1.8） | -2.6 | 0.10 |
| High school | 1.3（0.7~1.8） | 1.5（1.1~1.9） | 1.4（1.0~1.9） | 1.5（1.1~1.8） | 1.4（1.0~1.8） | 0.6 | 0.72 |
| College /university | 0.2（0.0~0.3） | 0.9（0.5~1.2） | 1.1（0.4~1.8） | 0.6（0.3~0.9） | 1.0（0.4~1.6） | 11.7 | 0.17 |
| **Occupation** |  |  |  |  |  |  |  |
| Agriculture | 2.4（1.2~3.6） | 2.6（1.7~3.5） | 2.2（1.7~2.7） | 2.6（1.9~3.3） | 2.6（1.8~3.3） | 0.5 | 0.89 |
| Manufacture | 1.3（0.5~2.2） | 1.1（0.5~1.7） | 1.0（0.5~1.5） | 1.3（0.7~1.9） | 0.7（0.2~1.1） | -4.0 | 0.38 |
| Service provider | 1.0（0.5~1.5） | 1.7（1.0~2.4） | 1.2（0.8~1.7） | 1.3（1.0~1.6） | 1.7（0.8~2.7） | 3.0 | 0.39 |
| Managers/professionals | 0.8（0.1~1.4） | 1.1（0.5~1.8） | 1.3（0.6~1.9） | 0.9（0.6~1.2） | 0.6（0.3~0.9） | -2.6 | 0.27 |
| Others | 4.3（1.9~6.7） | 2.2（1.4~3.0） | 1.9（1.2~2.7） | 1.4（0.9~1.9） | 1.9（1.0~2.7） | -7.5 | 0.02 |
| Unemployed/students | 1.8（1.2~2.4） | 2.5（1.9~3.0） | 2.1（1.6~2.6） | 1.8（1.4~2.1） | 2.0（1.2~2.8） | 0.3 | 0.62 |
| Retired | 3.0（1.9~4.1） | 2.5（1.8~3.3） | 3.1（2.3~3.9） | 2.5（1.7~3.3） | 2.3（1.6~3.0） | -1.9 | 0.45 |

CI, confidence interval. ^*^ Since 2010.

# **Table I. The prevalence of regular smoking among women in China, 2007-2018. Values are weighted percentages (95%CI).**

|  | **2007** | **2010** | **2013** | **2015** | **2018** | **Annual rate of change** | ***p* for trend** |
| --- | --- | --- | --- | --- | --- | --- | --- |
| **Overall** | 1.8（1.2~2.4） | 1.8（1.4~2.3） | 1.6（1.3~1.9） | 1.6（1.2~1.9） | 1.6（1.2~2.0） | -1.3 | 0.53 |
| **Age~ y** |  |  |  |  |  |  |  |
| 18-19 | 0.4（0.0~1.1） | 0.6（0.0~1.4） | 0.7（0.0~1.8） | 0.2（0.0~0.7） | 3.6（0.0~9.9） | 13.7 | 0.20 |
| 20-24 | 1.6（0.1~3.2） | 0.6（0.3~1.0） | 0.6（0.1~1.2） | 0.3（0.0~0.7） | 1.1（0.4~1.7） | -5.2 | 0.45 |
| 25-29 | 0.4（0.1~0.7） | 1.0（0.6~1.5） | 0.6（0.3~0.9） | 0.5（0.3~0.7） | 0.8（0.4~1.1） | 2.9 | 0.69 |
| 30-39 | 1.1（0.6~1.6） | 1.4（0.8~1.9） | 1.2（0.9~1.6） | 1.2（0.9~1.6） | 0.9（0.6~1.1） | -2.0 | 0.45 |
| 40-49 | 1.9（0.8~2.9） | 1.8（1.3~2.4） | 1.5（1.1~1.8） | 1.6（1.2~2.0） | 1.7（1.3~2.2） | -1.3 | 0.76 |
| 50-59 | 2.8（1.7~3.9） | 2.8（2.0~3.6） | 2.8（2.2~3.3） | 2.8（2.1~3.5） | 2.2（1.7~2.8） | -1.8 | 0.53 |
| 60-69 | 3.6（2.1~5.0） | 3.9（2.7~5.0） | 3.3（2.6~4.0） | 3.2（2.4~3.9） | 2.8（2.2~3.5） | -2.5 | 0.31 |
| **Birth cohort** |  |  |  |  |  |  |  |
| 1930-40s | 3.3（2.0~4.6） | 3.9（2.8~5.1） | 3.4（2.6~4.2） | 3.4（2.6~4.2） | 3.3（2.2~4.4） | -0.5 | 0.96 |
| 1950s | 2.8（1.5~4.1） | 2.8（2.0~3.7） | 3.1（2.5~3.8） | 3.1（2.3~3.9） | 3.0（2.3~3.7） | 0.9 | 0.73 |
| 1960s | 1.6（0.8~2.5） | 2.0（1.4~2.5） | 1.9（1.5~2.3） | 2.2（1.7~2.8） | 2.0（1.5~2.5） | 2.0 | 0.45 |
| 1970s | 0.8（0.5~1.1） | 1.4（0.9~1.9） | 1.3（0.9~1.6） | 1.5（1.1~1.9） | 1.8（1.3~2.3） | 6.5 | 0.01 |
| 1980s | 1.1（0.2~2.0） | 0.9（0.5~1.2） | 0.8（0.5~1.2） | 0.8（0.6~1.1） | 0.7（0.5~1.0） | -3.8 | 0.45 |
| 1990s | - | 0.6（0.0~1.2） | 0.5（0.0~1.0） | 0.3（0.0~0.6） | 1.2（0.4~1.9） | 6.9* | 0.16 |
| **Areas** |  |  |  |  |  |  |  |
| Urban | 1.5（1.0~1.9） | 1.8（1.3~2.4） | 1.8（1.4~2.3） | 1.3（0.9~1.6） | 1.6（1.0~2.1） | -0.5 | 0.67 |
| Rural | 1.9（1.0~2.9） | 1.8（1.1~2.4） | 1.4（1.1~1.8） | 1.8（1.2~2.3） | 1.6（1.1~2.1） | -1.4 | 0.63 |
| **Region** |  |  |  |  |  |  |  |
| North | 1.4（0.9~2.0） | 2.8（1.7~4.0） | 2.6（1.6~3.6） | 2.7（1.5~3.9） | 3.7（2.2~5.2） | 7.5 | 0.03 |
| Northeast | 8.3（5.2~11.4） | 7.3（5.1~9.5） | 5.8（4.4~7.2） | 6.4（4.4~8.3） | 6.5（4.5~8.6） | -2.4 | 0.33 |
| East | 0.8（0.5~1.1） | 0.8（0.5~1.0） | 0.7（0.5~1.0） | 0.6（0.5~0.8） | 0.5（0.3~0.6） | -4.4 | 0.04 |
| Middle | 0.9（0.2~1.7） | 1.1（0.4~1.7） | 1.1（0.6~1.7） | 0.9（0.4~1.5） | 0.8（0.4~1.2） | -1.5 | 0.75 |
| South | 0.3（0.1~0.5） | 0.6（0.3~0.9） | 0.8（0.3~1.2） | 1.2（0.5~1.9） | 0.7（0.3~1.1） | 9.3 | 0.05 |
| Southwest | 1.6（1.0~2.2） | 1.5（0.6~2.4） | 1.3（0.7~1.9） | 1.0（0.5~1.5） | 1.1（0.6~1.5） | -4.1 | 0.10 |
| Northwest | 0.9（0.3~1.5） | 0.9（0.4~1.4） | 0.5（0.3~0.7） | 0.4（0.2~0.7） | 0.5（0.2~0.7） | -7.1 | 0.13 |
| **Education** |  |  |  |  |  |  |  |
| No formal/primary school | 2.6（1.4~3.8） | 2.7（1.9~3.5） | 2.2（1.8~2.7） | 2.6（1.9~3.3） | 2.6（1.8~3.4） | 0.0 | 1.00 |
| Secondary school | 1.5（0.9~2.1） | 1.5（1.1~1.9） | 1.3（1.0~1.6） | 1.1（0.8~1.3） | 1.2（0.9~1.5） | -2.7 | 0.22 |
| High school | 0.9（0.5~1.4） | 1.2（0.8~1.5） | 1.0（0.7~1.3） | 1.1（0.8~1.4） | 1.1（0.7~1.5） | 1.2 | 0.74 |
| College /university | 0.1（0.0~0.2） | 0.5（0.2~0.7） | 0.9（0.2~1.6） | 0.5（0.2~0.8） | 0.8（0.3~1.3） | 17.5 | 0.09 |
| **Occupation** |  |  |  |  |  |  |  |
| Agriculture | 2.0（0.9~3.2） | 2.2（1.4~3.0） | 1.9（1.4~2.3） | 2.2（1.6~2.9） | 2.3（1.6~2.9） | 1.0 | 0.83 |
| Manufacture | 0.7（0.2~1.1） | 1.1（0.5~1.6） | 0.5（0.2~0.8） | 0.9（0.4~1.4） | 0.6（0.2~1.0） | -2.1 | 0.80 |
| Service provider | 0.5（0.3~0.8） | 1.1（0.5~1.7） | 0.8（0.5~1.1） | 1.1（0.8~1.4） | 1.3（0.4~2.2） | 7.3 | 0.11 |
| Managers/professionals | 0.6（0.0~1.3） | 0.8（0.2~1.4） | 1.0（0.3~1.6） | 0.7（0.5~1.0） | 0.4（0.2~0.6） | -3.2 | 0.21 |
| Others | 3.5（1.2~5.8） | 1.8（1.1~2.5） | 1.6（0.9~2.4） | 1.1（0.7~1.6） | 1.6（0.8~2.5） | -7.4 | 0.06 |
| Unemployed/students | 1.3（0.8~1.8） | 1.9（1.4~2.4） | 1.6（1.3~2.0） | 1.4（1.0~1.7） | 1.7（0.9~2.4） | 1.0 | 0.95 |
| Retired | 2.4（1.6~3.2） | 2.3（1.6~3.0） | 2.5（1.8~3.2） | 2.1（1.4~2.7） | 1.8（1.2~2.4） | -2.4 | 0.35 |

CI, confidence interval. ^*^ Since 2010.

# **Table J. The prevalence of occasional smoking among women in China, 2007-2018. Values are weighted percentages (95%CI).**

|  | **2007** | **2010** | **2013** | **2015** | **2018** | **Annual rate of change** | ***p* for trend** |
| --- | --- | --- | --- | --- | --- | --- | --- |
| **Overall** | 0.4（0.3~0.6） | 0.4（0.3~0.5） | 0.4（0.3~0.5） | 0.3（0.2~0.4） | 0.3（0.2~0.4） | -3.0 | 0.04 |
| **Age~ y** |  |  |  |  |  |  |  |
| 18-19 | - | 0.4（0.0~0.8） | 0.3（0.0~0.7） | - | 0.7（0.0~1.9） | 8.3 | 0.32 |
| 20-24 | 0.0（0.0~0.1） | 0.4（0.1~0.7） | 0.4（0.2~0.6） | 0.1（0.0~0.3） | 0.1（0.0~0.2） | -18.4 | 0.48 |
| 25-29 | 0.3（0.0~0.8） | 0.4（0.1~0.6） | 0.3（0.2~0.4） | 0.1（0.0~0.3） | 0.3（0.1~0.6） | -4.5 | 0.51 |
| 30-39 | 0.4（0.1~0.8） | 0.5（0.3~0.6） | 0.4（0.3~0.5） | 0.4（0.1~0.6） | 0.4（0.2~0.6） | -0.8 | 0.66 |
| 40-49 | 0.6（0.3~0.9） | 0.4（0.3~0.5） | 0.5（0.4~0.7） | 0.4（0.2~0.5） | 0.2（0.2~0.3） | -7.8 | 0.01 |
| 50-59 | 0.6（0.3~0.8） | 0.4（0.3~0.5） | 0.5（0.3~0.6） | 0.4（0.3~0.6） | 0.4（0.3~0.5） | -2.9 | 0.34 |
| 60-69 | 0.7（0.3~1.2） | 0.6（0.3~0.8） | 0.3（0.0~0.7） | 0.5（0.3~0.7） | 0.4（0.3~0.5） | -5.0 | 0.16 |
| **Birth cohort** |  |  |  |  |  |  |  |
| 1930-40s | 0.8（0.4~1.3） | 0.6（0.4~0.9） | 0.5（0.3~0.8） | 0.7（0.3~1.1） | 0.5（0.1~0.8） | -3.1 | 0.38 |
| 1950s | 0.5（0.3~0.7） | 0.4（0.2~0.5） | 0.5（0.3~0.6） | 0.4（0.3~0.5） | 0.4（0.2~0.5） | -1.6 | 0.35 |
| 1960s | 0.5（0.3~0.7） | 0.4（0.3~0.5） | 0.5（0.3~0.6） | 0.5（0.3~0.7） | 0.4（0.3~0.5） | -0.9 | 0.52 |
| 1970s | 0.5（0.0~.01） | 0.5（0.3~0.7） | 0.3（0.2~0.4） | 0.4（0.2~0.5） | 0.3（0.2~0.4） | -4.7 | 0.05 |
| 1980s | 0.0（0.0~.0.0） | 0.4（0.2~0.6） | 0.4（0.1~0.7） | 0.2（0.1~0.4） | 0.4（0.2~0.5） | -2.0 | 0.03 |
| 1990s | - | 0.3（0.0~0.7） | 0.2（0.1~0.4） | 0.1（0.0~0.2） | 0.2（0.1~0.4） | -6.6* | 0.82 |
| **Areas** |  |  |  |  |  |  |  |
| Urban | 0.6（0.3~0.9） | 0.5（0.4~0.7） | 0.6（0.4~0.7） | 0.3（0.2~0.4） | 0.3（0.2~0.4） | -6.5 | ＜0.001 |
| Rural | 0.4（0.2~0.5） | 0.4（0.2~0.5） | 0.3（0.2~0.4） | 0.3（0.2~0.4） | 0.3（0.2~0.4） | -3.2 | 0.60 |
| **Region** |  |  |  |  |  |  |  |
| North | 0.3（0.2~0.4） | 1.2（0.8~1.6） | 0.4（0.2~0.5） | 0.6（0.2~1.1） | 0.6（0.3~0.9） | 2.7 | 0.13 |
| Northeast | 0.9（0.3~1.5） | 0.2（0.1~0.3） | 0.8（0.4~1.1） | 0.6（0.3~0.8） | 0.5（0.3~0.6） | -0.4 | 0.009 |
| East | 0.3（0.1~0.4） | 0.4（0.2~0.6） | 0.3（0.1~0.5） | 0.2（0.1~0.3） | 0.1（0.1~0.2） | -9.9 | 0.14 |
| Middle | 0.5（0.2~0.7） | 0.4（0.1~0.6） | 0.4（0.2~0.5） | 0.4（0.2~0.6） | 0.3（0.1~0.5） | -3.8 | 0.38 |
| South | 0.3（0.0~0.6） | 0.4（0.1~0.7） | 0.3（0.1~0.6） | 0.2（0.1~0.4） | 0.3（0.1~0.5） | -2.3 | 0.69 |
| Southwest | 0.7（0.0~1.4） | 0.2（0.1~0.4） | 0.4（0.2~0.7） | 0.3（0.1~0.5） | 0.5（0.3~0.8） | -1.1 | 0.44 |
| Northwest | 0.2（0.0~0.4） | 1.2（0.8~1.6） | 0.3（0.1~0.6） | 0.3（0.1~0.4） | 0.1（0.0~0.1） | -9.5 | 0.13 |
| **Education** |  |  |  |  |  |  |  |
| No formal/primary school | 0.6（0.4~0.9） | 0.5（0.3~0.6） | 0.4（0.3~0.5） | 0.5（0.3~0.6） | 0.4（0.3~0.6） | -3.1 | 0.28 |
| Secondary school | 0.4（0.1~0.6） | 0.5（0.3~0.6） | 0.4（0.3~0.5） | 0.2（0.1~0.3） | 0.3（0.2~0.4） | -5.1 | 0.10 |
| High school | 0.3（0.1~0.5） | 0.3（0.2~0.4） | 0.5（0.2~0.8） | 0.4（0.2~0.6） | 0.3（0.2~0.4） | 1.2 | 0.80 |
| College /university | 0.1（0.0~0.2） | 0.4（0.2~0.6） | 0.2（0.0~0.4） | 0.1（0.0~0.2） | 0.2（0.1~0.3） | 0.6 | 0.47 |
| **Occupation** |  |  |  |  |  |  |  |
| Agriculture | 0.4（0.2~0.5） | 0.4（0.3~0.6） | 0.3（0.2~0.4） | 0.4（0.2~0.5） | 0.3（0.2~0.4） | -2.3 | 0.61 |
| Manufacture | 0.7（0.0~1.3） | 0.0（0.0~0.1） | 0.5（0.1~1.0） | 0.4（0.1~0.7） | 0.1（0.0~0.1） | -14.5 | 0.25 |
| Service provider | 0.5（0.1~0.9） | 0.6（0.3~0.9） | 0.4（0.2~0.7） | 0.2（0.1~0.3） | 0.5（0.1~0.8） | -3.7 | 0.45 |
| Managers/professionals | 0.1（0.0~0.2） | 0.3（0.1~0.5） | 0.3（0.1~0.5） | 0.2（0.0~0.3） | 0.2（0.1~0.4） | 4.2 | 0.97 |
| Others | 0.8（0.2~1.4） | 0.4（0.2~0.7） | 0.3（0.2~0.4） | 0.3（0.1~0.4） | 0.2（0.0~0.4） | -10.9 | 0.01 |
| Unemployed/students | 0.5（0.1~0.8） | 0.6（0.4~0.7） | 0.5（0.2~0.7） | 0.4（0.3~0.5） | 0.3（0.2~0.5） | -5.0 | 0.05 |
| Retired | 0.6（0.1~1.2） | 0.2（0.1~0.4） | 0.6（0.3~0.9） | 0.4（0.2~0.7） | 0.5（0.3~0.8） | -1.2 | 0.93 |

CI, confidence interval. ^*^ Since 2010.

# **Table K. The prevalence of former smoking among women in China, 2007-2018. Values are weighted percentages (95%CI).**

|  | **2007** | **2010** | **2013** | **2015** | **2018** | **Annual rate of change** | ***p* for trend** |
| --- | --- | --- | --- | --- | --- | --- | --- |
| **Overall** | 0.6（0.4~0.7） | 0.6（0.4~0.7） | 0.4（0.3~0.5） | 0.5（0.4~0.6） | 0.5（0.3~0.6） | -2.1 | <0.001 |
| **Age~ y** |  |  |  |  |  |  |  |
| 18-19 | 0.0（0.0~0.0） | 0.3（0.0~0.6） | 0.1（0.0~0.3） | 0.3（0.0~0.8） | 0.9（0.0~2.2） | 17.5 | 0.92 |
| 20-24 | 0.4（0.0~0.9） | 0.5（0.2~0.9） | 0.2（0.0~0.3） | 0.3（0.1~0.5） | 0.4（0.0~0.9） | -2.1 | 0.46 |
| 25-29 | 0.5（0.1~1.0） | 0.6（0.3~0.9） | 0.3（0.1~0.5） | 0.1（0.0~0.2） | 0.3（0.0~0.5） | -9.5 | 0.06 |
| 30-39 | 0.3（0.1~0.4） | 0.3（0.2~0.4） | 0.2（0.1~0.3） | 0.2（0.1~0.3） | 0.3（0.2~0.5） | -1.5 | <0.001 |
| 40-49 | 0.2（0.1~0.4） | 0.4（0.2~0.5） | 0.3（0.2~0.4） | 0.4（0.3~0.5） | 0.3（0.2~0.5） | 3.1 | <0.001 |
| 50-59 | 0.9（0.6~1.2） | 1.2（0.8~1.5） | 0.6（0.4~0.7） | 0.7（0.5~0.9） | 0.5（0.4~0.7） | -6.2 | <0.001 |
| 60-69 | 2.1（1.2~3.0） | 2.1（1.4~2.7） | 1.1（0.8~1.4） | 1.3（0.9~1.7） | 1.0（0.7~1.3） | -7.1 | 0.07 |
| **Birth cohort** |  |  |  |  |  |  |  |
| 1930-40s | 1.8（1.1~2.5） | 2.3（1.5~3.0） | 1.2（0.8~1.5） | 1.4（0.9~1.9） | 0.8（0.3~1.3） | -7.6 | <0.001 |
| 1950s | 0.6（0.4~0.9） | 1.1（0.8~1.5） | 0.8（0.5~1.0） | 1.0（0.7~1.3） | 1.0（0.7~1.3） | 3.5 | <0.001 |
| 1960s | 0.2（0.1~0.4） | 0.4（0.3~0.5） | 0.4（0.2~0.5） | 0.6（0.5~0.8） | 0.4（0.3~0.6） | 6.9 | <0.001 |
| 1970s | 0.5（0.2~0.8） | 0.3（0.1~0.4） | 0.2（0.1~0.3） | 0.3（0.2~0.4） | 0.3（0.2~0.5） | -4.0 | <0.001 |
| 1980s | 0.2（0.0~0.5） | 0.5（0.3~0.8） | 0.2（0.1~0.4） | 0.2（0.1~0.2） | 0.3（0.2~0.5） | 0.3 | 0.008 |
| 1990s | - | 0.4（0.1~0.8） | 0.2（0.0~0.3） | 0.3（0.1~0.5） | 0.4（0.1~0.8） | 1.2^*^ | 0.02 |
| **Areas** |  |  |  |  |  |  |  |
| Urban | 0.5（0.3~0.8） | 0.8（0.6~1.0） | 0.4（0.3~0.5） | 0.4（0.3~0.5） | 0.4（0.2~0.6） | -4.1 | <0.001 |
| Rural | 0.6（0.4~0.8） | 0.6（0.4~0.8） | 0.4（0.3~0.5） | 0.5（0.4~0.7） | 0.5（0.3~0.6） | -2.1 | 0.05 |
| **Region** |  |  |  |  |  |  |  |
| North | 0.6（0.2~1.0） | 0.7（0.4~1.1） | 0.6（0.3~0.8） | 0.8（0.4~1.1） | 0.9（0.3~1.4） | 3.4 | 0.24 |
| Northeast | 1.1（0.5~1.7） | 1.6（1.2~2.1） | 0.9（0.6~1.1） | 1.2（0.8~1.5） | 1.0（0.5~1.6） | -1.8 | <0.001 |
| East | 0.4（0.1~0.6） | 0.5（0.3~0.6） | 0.2（0.1~0.3） | 0.3（0.2~0.4） | 0.2（0.1~0.3） | -7.0 | ＜0.001 |
| Middle | 0.2（0.1~0.3） | 0.7（0.2~1.1） | 0.3（0.1~0.5） | 0.2（0.1~0.4） | 0.3（0.1~0.4） | 1.2 | 0.13 |
| South | 0.6（0.0~1.2） | 0.6（0.2~1.1） | 0.4（0.1~0.7） | 0.4（0.0~0.8） | 0.3（0.0~0.5） | -6.4 | 0.65 |
| Southwest | 1.0（0.7~1.3） | 0.7（0.1~1.3） | 0.5（0.3~0.6） | 0.5（0.3~0.7） | 0.8（0.3~1.3） | -3.0 | 0.16 |
| Northwest | 0.5（0.2~0.9） | 0.4（0.1~0.7） | 0.1（0.0~0.2） | 0.2（0.1~0.4） | 0.2（0.1~0.3） | -9.4 | 0.85 |
| **Education** |  |  |  |  |  |  |  |
| No formal/primary school | 0.8（0.5~1.0） | 0.9（0.7~1.2） | 0.6（0.4~0.7） | 0.8（0.6~1.0） | 0.7（0.4~1.0） | -1.5 | 0.005 |
| Secondary school | 0.4（0.2~0.6） | 0.6（0.4~0.8） | 0.3（0.2~0.3） | 0.3（0.2~0.4） | 0.3（0.2~0.5） | -4.6 | 0.04 |
| High school | 0.5（0.2~0.9） | 0.5（0.3~0.6） | 0.3（0.2~0.5） | 0.3（0.1~0.4） | 0.4（0.2~0.7） | -3.5 | 0.29 |
| College /university | 0.3（0.0~0.6） | 0.4（0.1~0.7） | 0.2（0.0~0.3） | 0.2（0.1~0.3） | 0.2（0.0~0.5） | -5.4 | <0.001 |
| **Occupation** |  |  |  |  |  |  |  |
| Agriculture | 0.6（0.4~0.8） | 0.7（0.5~0.9） | 0.5（0.3~0.6） | 0.6（0.4~0.7） | 0.6（0.4~0.8） | -0.6 | 0.003 |
| Manufacture | 0.3（0.0~0.7） | 0.4（0.0~0.7） | 0.3（0.1~0.5） | 0.1（0.0~0.2） | 0.1（0.0~0.2） | -12.0 | ＜0.001 |
| Service provider | 0.4（0.1~0.7） | 0.5（0.2~0.8） | 0.3（0.0~0.6） | 0.2（0.1~0.4） | 0.6（0.2~1.0） | 0.2 | <0.001 |
| Managers/professionals | 0.4（0.0~0.9） | 0.4（0.2~0.6） | 0.1（0.0~0.3） | 0.3（0.1~0.5） | 0.2（0.0~0.4） | -6.6 | <0.001 |
| Others | 0.5（0.1~0.8） | 0.6（0.3~0.9） | 0.3（0.1~0.5） | 0.3（0.1~0.5） | 0.3（0.1~0.5） | -6.2 | 0.004 |
| Unemployed/students | 0.8（0.3~1.4） | 0.9（0.6~1.2） | 0.4（0.3~0.5） | 0.5（0.4~0.7） | 0.5（0.2~0.8） | -5.6 | 0.006 |
| Retired | 1.0（0.5~1.5） | 1.3（1.0~1.7） | 0.6（0.3~0.9） | 0.7（0.4~1.0） | 0.6（0.3~0.9） | -6.0 | 0.51 |

CI, confidence interval. ^*^ Since 2010.

# **Table L. Percentages of former smokers among ever smokers among men and women in China, 2007-2018. Values are weighted percentages (95%CI).**

|  | **Men** | | | | | | |  | **Women** | | | | | | |
| --- | --- | --- | --- | --- | --- | --- | --- | --- | --- | --- | --- | --- | --- | --- | --- |
|  | **2007 (N=15862)** | **2010 (N=26921)** | **2013 (N=42950)** | **2015 (N=521079)** | **2018 (N=45452)** | **Annual rate of change** | ***p* for trend** |  | **2007 (N=929)** | **2010 (N=1844)** | **2013 (N=2996)** | **2015 (N=2969)** | **2018 (N=2877)** | **Annual rate of change** | ***p* for trend** |
| **Overall** | 11.3  (10.1~12.6) | 13.4  (12.5~14.3) | 12.7  (11.9~13.5) | 14.1  (13.2~14.9) | 13.4  (12.6~14.1) | 1.5 | 0.01 |  | 20.6  (15.5~25.6) | 23.7  (19.9~27.4) | 16.2  (13.5~18.9) | 19.5  (16.7~22.2) | 19.5  (15.4~23.7) | -1.2 | 0.42 |
| **Age~ y** |  |  |  |  |  |  |  |  |  |  |  |  |  |  |  |
| 18-19 | 4.2  (0.0~8.5) | 6.3  (3.0~9.6) | 3.1  (0.8~5.3) | 6.8  (3.2~10.4) | 8.8  (0.3~17.4) | 5.6 | 0.35 |  | 0.0  (0.0~0.0) | 23.1  (0.0~46.7) | 10.0  (0.0~27.5) | 52.8  (0.0~100.0) | 17.8  (0.0~48.7) | 1.8 | 0.85 |
| 20-24 | 7.2  (4.2~10.2) | 7.4  (5.6~9.1) | 4.9  (3.3~6.4) | 5.5  (3.6~7.4) | 5.9  (3.4~8.3) | -2.6 | 0.30 |  | 19.3  (0.0~45.4) | 33.7  (15.1~52.4) | 16.8  (2.2~31.4) | 35.5  (10.3~60.7) | 27.0  (7.7~46.4) | 2.4 | 0.73 |
| 25-29 | 5.0  (2.8~7.2) | 7.8  (6.3~9.3) | 5.4  (4.0~6.9) | 11.1  (6.4~15.9) | 5.1  (3.4~6.8) | 1.2 | 0.36 |  | 43.4  (24.6~62.2) | 29.7  (16.4~43.0) | 22.2  (8.6~35.9) | 17.1  (4.3~30.0) | 20.5  (5.0~36.0) | -7.3 | 0.08 |
| 30-39 | 7.8  (6.5~9.0) | 9.5  (8.5~10.6) | 9.0  (7.8~10.1) | 9.2  (8.0~10.4) | 9.3  (7.9~10.7) | 1.2 | 0.23 |  | 15.4  (7.5~23.4) | 13.5  (7.9~19.2) | 11.4  (5.5~17.2) | 11.9  (7.1~16.7) | 21.4  (11.9~31.0) | 1.9 | 0.50 |
| 40-49 | 10.4  (8.8~12.0) | 12.7  (11.6~13.8) | 12.7  (11.5~13.8) | 13.3  (12.1~14.5) | 12.7  (11.6~13.8) | 1.7 | 0.03 |  | 8.3  (3.6~13.0) | 14.0  (10.1~17.9) | 14.3  (10.0~18.6) | 17.3  (12.8~21.8) | 14.9  (9.4~20.4) | 5.3 | 0.05 |
| 50-59 | 15.0  (12.7~17.4) | 17.4  (16.2~18.6) | 18.0  (16.8~19.1) | 18.8  (17.5~20.0) | 18.4  (17.3~19.6) | 1.8 | 0.009 |  | 20.7  (14.7~26.8) | 26.8  (21.9~31.6) | 14.6  (11.1~18.0) | 17.9  (14.1~21.7) | 16.4  (12.6~20.1) | -3.2 | 0.06 |
| 60-69 | 24.8  (20.8~28.9) | 27.0  (24.7~29.4) | 26.0  (24.2~27.9) | 29.1  (27.4~30.8) | 28.0  (26.3~29.7) | 1.2 | 0.13 |  | 32.2  (19.7~44.7) | 31.6  (25.3~37.9) | 22.5  (18.3~26.7) | 26.1  (21.0~31.1) | 23.6  (19.3~27.9) | -3.1 | 0.16 |
| **Areas** |  |  |  |  |  |  |  |  |  |  |  |  |  |  |  |
| Urban | 12.0  (10.0~14.0) | 13.4  (12.1~14.6) | 12.8  (11.7~13.9) | 14.1  (13.0~15.2) | 13.7  (12.6~14.9) | 1.2 | 0.16 |  | 20.4  (14.8~26.1) | 25.3  (20.5~30.1) | 13.6  (9.5~17.7) | 19.0  (14.7~23.3) | 19.2  (12.7~25.7) | -1.7 | 0.34 |
| Rural | 11.0  (9.3~12.6) | 13.4  (12.1~14.6) | 12.7  (11.7~13.7) | 14.1  (12.9~15.3) | 13.1  (12.1~14.1) | 1.5 | 0.04 |  | 20.6  (13.6~27.7) | 22.6  (17.4~27.9) | 18.1  (14.5~21.7) | 19.7  (16.2~23.2) | 19.8  (14.4~25.1) | -0.8 | 0.68 |
| **Education** |  |  |  |  |  |  |  |  |  |  |  |  |  |  |  |
| No formal/primary school | 12.4  (10.6~14.3) | 16.2  (14.8~17.6) | 14.8  (13.7~15.9) | 16.3  (15.1~17.5) | . 15.2  (14.2~16.2) | 1.6 | 0.03 |  | 19.5  (12.8~26.1) | 23.0  (18.1~27.8) | 17.7  (14.6~20.8) | 20.3  (16.8~23.7) | 18.7  (12.7~24.8) | -0.8 | 0.73 |
| Secondary school | 10.1  (8.6~11.6) | 12.1  (11.0~13.2) | 11.5  (10.6~12.4) | 12.7  (11.7~13.7) | . 12.5  (11.4~13.5) | 1.8 | 0.01 |  | 17.1  (9.3~25.0) | 24.1  (17.8~30.5) | 13.2  (9.1~17.4) | 18.6  (14.1~23.1) | 18.5  (11.1~25.9) | -0.5 | 0.74 |
| High school | 11.7  (9.5~13.8) | 11.9  (10.7~13.1) | 12.9  (11.5~14.3) | 14.6  (12.4~16.8) | 12.2  (10.7~13.7) | 1.0 | 0.28 |  | 30.3  (17.7~43.0) | 23.9  (15.8~32.0) | 18.0  (9.5~26.4) | 16.0  (7.8~24.2) | 24.4  (12.9~35.9) | -3.1 | 0.35 |
| College/university | 12.7  (10.1~15.4) | 13.3  (11.1~15.4) | 11.4  (9.0~13.8) | 12.6  (10.5~14.7) | 14.5  (12.5~16.4) | 0.7 | 0.37 |  | 63.0  (33.7~92.2) | 29.7  (10.8~48.6) | 13.6  (0.3~26.9) | 21.2  (6.9~35.6) | 19.9  (7.7~32.2) | -9.7 | 0.13 |
| **Occupation** |  |  |  |  |  |  |  |  |  |  |  |  |  |  |  |
| Agriculture | 11.3  (9.5~13.2) | 13.5  (12.2~14.8) | 13.2  (12.1~14.2) | 14.0  (12.8~15.2) | 13.9  (12.8~15.0) | 1.7 | 0.03 |  | 18.4  (10.8~26.0) | 20.5  (15.1~26.0) | 17.2  (13.7~20.7) | 17.6  (14.7~20.5) | 18.6  (13.1~24.1) | -0.5 | 0.82 |
| Manufacture | 7.9  (6.1~9.6) | 9.1  (7.4~10.7) | 11.1  (9.5~12.8) | 12.1  (9.9~14.2) | 10.2  (8.6~11.8) | 3.0 | 0.004 |  | 19.9  (2.6~37.1) | 24.7  (2.8~46.6) | 22.8  (6.5~39.1) | 5.4  (0.0~11.0) | 11.5  (0.0~25.3) | -8.6 | 0.21 |
| Service provider | 9.8  (6.9~12.8) | 12.2  (10.5~14.0) | 10.2  (8.4~12.0) | 11.7  (9.3~14.0) | 13.1  (11.0~15.3) | 2.0 | 0.20 |  | 26.3  (8.3~44.3) | 23.0  (9.8~36.2) | 20.1  (3.8~36.3) | 14.6  (5.4~23.7) | 24.5  (7.3~41.7) | -2.1 | 0.79 |
| Managers/professionals | 10.2  (8.0~12.3) | 12.1  (10.6~13.6) | 13.1  (11.5~14.7) | 13.0  (10.9~15.2) | 12.5  (10.7~14.3) | 1.8 | 0.11 |  | 35.2  (1.1~69.3) | 26.0  (10.7~41.4) | 10.2  (1.2~19.2) | 25.3  (11.3~39.2) | 25.8  (3.5~48.1) | -2.9 | 0.73 |
| Others | 9.1  (6.6~11.6) | 10.9  (9.4~12.5) | 9.7  (8.3~11.1) | 11.3  (9.8~12.8) | 10.3  (9.0~11.7) | 1.0 | 0.35 |  | 9.9  (2.5~17.3) | 20.6  (10.7~30.6) | 13.4  (3.2~23.6) | 18.1  (10.9~25.3) | 12.7  (2.7~22.8) | 1.4 | 0.55 |
| Unemployed/students | 12.2  (9.2~15.2) | 15.1  (12.6~17.7) | 11.2  (9.6~12.8) | 18.6  (14.6~22.5) | 13.8  (11.5~16.1) | 1.5 | 0.25 |  | 31.7  (17.6~45.8) | 26.8  (21.0~32.5) | 15.3  (11.1~19.6) | 23.2  (17.7~28.8) | 20.0  (9.2~30.8) | -4.1 | 0.21 |
| Retired | 31.1  (27.0~35.2) | 33.4  (29.9~36.8) | 32.8  (29.2~36.3) | 32.2  (29.7~34.7) | 30.1  (26.0~34.2) | -0.4 | 0.76 |  | 24.8  (13.6~36.1) | 34.6  (26.0~43.3) | 16.6  (9.2~24.0) | 22.0  (15.4~28.5) | 19.9  (12.0~27.8) | -3.4 | 0.22 |

CI, confidence interval.

# **Table M. The prevalence of current smoking among adults with and without major NCDs in China, 2007-2018. Values are weighted percentages (95%CI).**

|  |  | **With major NCDs** | | | | | | |  | **Without major NCDs** | | | | | | |
| --- | --- | --- | --- | --- | --- | --- | --- | --- | --- | --- | --- | --- | --- | --- | --- | --- |
|  |  | **2007** | **2010** | **2013** | **2015** | **2018** | **Annual rate of change** | ***p* for trend** |  | **2007** | **2010** | **2013** | **2015** | **2018** | **Annual rate of change** | ***p* for trend** |
| **Men** | | 53.9  (51.3~56.6) | 50.5  (48.7~52.3) | 49.1  (47.4~50.7) | 51.2  (49.5~52.8) | 50.8  (49.0~52.6) | -0.4 | 0.22 |  | 58.9  (56.5~61.4) | 54.6  (52.6~56.6) | 53.8  (52.2~55.5) | 53.6  (52.1~55.2) | 50.8  (48.9~52.8) | -1.2 | <0.001 |
| **Areas** | |  |  |  |  |  |  |  |  |  |  |  |  |  |  |  |
|  | Urban | 54.2  (50.4~58.0) | 51.0  (48.1~53.9) | 49.6  (47.0~52.2) | 50.3  (47.6~53.0) | 48.9  (46.6~51.3) | -0.8 | 0.04 |  | 56.0  (51.2~60.7) | 53.1  (50.0~56.3) | 48.5  (46.0~51.0) | 47.9  (45.7~50.2) | 45.8  (42.8~48.8) | -1.9 | <0.001 |
|  | Rural | 53.7  (50.1~57.4) | 50.2  (47.9~52.5) | 48.7  (46.6~50.8) | 51.8  (49.7~53.9) | 52.3  (49.7~54.9) | -0.1 | 0.99 |  | 60.7  (57.9~63.4) | 55.4  (52.9~58.0) | 56.8  (54.8~58.7) | 57.8  (56.0~59.5) | 55.0  (52.7~57.3) | -0.6 | 0.06 |
| **Education** | |  |  |  |  |  |  |  |  |  |  |  |  |  |  |  |
|  | No formal/primary school | 51.2  (46.8~55.6) | 50.4  (47.9~53.0) | 49.2  (46.7~51.7) | 51.1  (48.9~53.4) | 52.4  (49.1~55.7) | 0.2 | 0.91 |  | 65.8  (62.9~68.7) | 60.2  (57.6~62.9) | 59.8  (57.9~61.8) | 60.7  (58.5~62.8) | 59.1  (57.0~61.2) | -0.8 | 0.004 |
|  | Secondary school | 59.7  (56.3~63.0) | 50.9  (48.2~53.7) | 50.9  (48.4~53.4) | 53.4  (51.0~55.7) | 53.1  (50.8~55.4) | -0.7 | 0.20 |  | 60.5  (57.9~63.1) | 56.7  (54.3~59.1) | 57.0  (55.0~58.9) | 58.6  (56.9~60.4) | 55.9  (53.8~58.0) | -0.5 | 0.04 |
|  | High school | 52.4  (47.2~57.6) | 52.3  (48.7~55.9) | 48.2  (44.8~51.7) | 51.1  (48.0~54.3) | 49.2  (45.4~52.9) | -0.6 | 0.35 |  | 53.7  (50.8~56.6) | 51.5  (49.2~53.8) | 48.7  (46.0~51.3) | 50.7  (47.8~53.6) | 51.5  (48.0~55.0) | -0.4 | 0.34 |
|  | College/university | 46.4  (40.6~52.2) | 46.1  (42.2~49.9) | 41.0  (35.8~46.1) | 43.3  (36.9~49.6) | 40.5  (34.8~46.2) | -1.3 | 0.09 |  | 39.8  (35.7~44.0) | 40.5  (37.3~43.6) | 38.6  (35.2~41.9) | 35.7  (33.2~38.3) | 32.0  (28.8~35.3) | -2.0^*^ | ＜0.001 |
|  |  |  |  |  |  |  |  |  |  |  |  |  |  |  |  |  |
| **Women** | | 2.9  (1.8~4.1) | 3.7  (2.8~4.6) | 3.3  (2.5~4.0) | 3.0  (2.4~3.6) | 3.4  (2.1~4.6) | -0.5 | 0.99 |  | 2.1  (1.4~2.8) | 2.0  (1.5~2.5) | 1.8  (1.5~2.1) | 1.7  (1.4~2.1) | 1.6  (1.3~2.0) | -2.6 | 0.27 |
| **Areas** | |  |  |  |  |  |  |  |  |  |  |  |  |  |  |  |
|  | Urban | 2.1  (1.4~2.9) | 3.6  (2.3~4.8) | 3.8  (2.2~5.4) | 3.0  (2.1~3.9) | 4.2  (1.6~6.7) | 4.8 | 0.29 |  | 2.0  (1.4~2.6) | 2.2  (1.6~2.8) | 2.1  (1.6~2.7) | 1.4  (1.1~1.8) | 1.5  (1.2~1.9) | -3.5 | 0.03 |
|  | Rural | 3.5  (1.6~5.3) | 3.8  (2.6~5.0) | 2.9  (2.2~3.7) | 3.0  (2.1~3.9) | 2.8  (1.8~3.7) | -2.5 | 0.35 |  | 2.2  (1.2~3.2) | 1.9  (1.2~2.6) | 1.6  (1.2~1.9) | 1.9  (1.4~2.5) | 1.7  (1.2~2.3) | -2.0 | 0.65 |
| **Education** | |  |  |  |  |  |  |  |  |  |  |  |  |  |  |  |
|  | No formal/primary school | 3.9  (2.1~5.7) | 4.2  (2.9~5.4) | 3.5  (2.7~4.3) | 3.8  (2.9~4.7) | 3.9  (1.9~6.0) | -0.4 | 0.93 |  | 3.0  (1.6~4.4) | 2.9  (2.0~3.8) | 2.4  (1.9~2.9) | 2.9  (2.2~3.7) | 2.8  (2.0~3.5) | -0.6 | 0.73 |
|  | Secondary school | 1.8  (1.0~2.7) | 3.6  (2.4~4.8) | 2.4  (1.7~3.2) | .2.1  (1.6~2.7) | 2.4  (1.7~3.2) | 0.3 | 0.64 |  | 1.8  (1.1~2.5) | 1.8  (1.3~2.2) | 1.6  (1.3~1.9) | 1.2  (0.9~1.5) | 1.3  (1.0~1.6) | -3.7 | 0.02 |
|  | High school | 0.5  (0.0~1.0) | 2.7  (1.6~3.8) | 2.5  (1.6~3.4) | 1.9  (0.8~3.1) | 3.9  (1.6~6.3) | 15.5 | 0.008 |  | 1.3  (0.7~1.8) | 1.3  (0.9~1.7) | 1.3  (0.8~1.8) | 1.4  (1.1~1.8) | 1.1  (0.7~1.5) | -1.0 | 0.66 |
|  | College/university | 0.0  (0.0~0.0) | 1.5  (0.4~2.5) | 6.8  (0.0~15.4) | 0.5  (0.1~0.9) | 0.8  (0.0~1.5) | -14.0 | 0.10 |  | 0.2  (0.0~0.3) | 0.8  (0.4~1.2) | 0.7  (0.3~1.2) | 0.6  (0.3~0.9) | 1.0  (0.4~1.6) | 11.9 | 0.09 |

CI, confidence interval; NCDs, noncommunicable diseases. Major NCDs refer to any of hypertension, diabetes, stroke, myocardial infarction, and chronic obstructive pulmonary disease (COPD).

# **Table N. The prevalence of current and former smoking among men with at least one NCD or specific major NCDs, 2007-2018.**

|  | **2007** | |  | **2010** | |  | **2013** | |  | **2015** | |  | **2018** | | ***p* for trend** |
| --- | --- | --- | --- | --- | --- | --- | --- | --- | --- | --- | --- | --- | --- | --- | --- |
|  | **N** | **% (95%CI)** |  | **N** | **% (95%CI)** |  | **N** | **% (95%CI)** |  | **N** | **% (95%CI)** |  | **N** | **% (95%CI)** |  |
| **Current smoking** |  |  |  |  |  |  |  |  |  |  |  |  |  |  |  |
| **Total** |  |  |  |  |  |  |  |  |  |  |  |  |  |  |  |
| At least one NCD | 3206 | 53.9(51.3~56.6) |  | 7370 | 50.5(48.7~52.3) |  | 14317 | 49.1(47.4~50.7) |  | 17056 | 51.2(49.5~52.8) |  | 19668 | 50.8(49.0~52.6) | 0.22 |
| Hypertension | 2150 | 51.3(47.9~54.7) |  | 5168 | 49.5(47.5~51.5) |  | 10140 | 48.7(46.9~50.4) |  | 10854 | 49.7(47.8~51.6) |  | 12283 | 51.4(49.0~53.8) | 0.79 |
| Diabetes | 441 | 49.4(44.0~54.8) |  | 1656 | 45.4(42.7~48.0) |  | 3383 | 45.1(42.3~47.8) |  | 2943 | 50.7(47.3~54.1) |  | 4515 | 46.5(43.8~49.3) | 0.67 |
| MI | 128 | 46.8(33.3~60.2) |  | 217 | 52.4(44.5~60.4) |  | 573 | 40.4(35.2~45.5) |  | 665 | 41.9(36.6~47.1) |  | 825 | 46.5(36.0~57.0) | 0.27 |
| Stroke | 241 | 43.6(36.4~50.8) |  | 260 | 44.5(37.2~51.9) |  | 1248 | 42.0(37.1~46.9) |  | 2590 | 44.0(40.8~47.2) |  | 3468 | 48.1(41.8~54.5) | 0.47 |
| COPD | 785 | 59.4(54.1~64.6) |  | 1276 | 57.6(53.4~61.7) |  | 2052 | 51.7(47.6~55.8) |  | 4298 | 55.5(52.3~58.6) |  | 4461 | 51.7(48.4~54.9) | 0.005 |
| **Urban** |  |  |  |  |  |  |  |  |  |  |  |  |  |  |  |
| At least one NCD | 1457 | 54.2(50.4~58.0) |  | 3262 | 51.0(48.1~53.9) |  | 6290 | 49.6(47.0~52.2) |  | 7694 | 50.3(47.6~53.0) |  | 9406 | 48.9(46.6~51.3) | 0.04 |
| Hypertension | 1044 | 51.5(47.7~55.2) |  | 2261 | 48.1(44.7~51.5) |  | 4414 | 49.3(46.6~52.0) |  | 5185 | 50.7(47.8~53.7) |  | 6069 | 50.2(47.3~53.2) | 0.88 |
| Diabetes | 284 | 48.8(42.2~55.5) |  | 930 | 48.5(45.4~51.7) |  | 1784 | 47.4(43.8~51.1) |  | 1723 | 50.9(46.3~55.4) |  | 2672 | 45.8(42.5~49.2) | 0.55 |
| MI | 66 | 53.2(35.3~71.2) |  | 87 | 55.6(43.2~68.0) |  | 274 | 43.2(35.7~50.6) |  | 338 | 48.0(41.5~54.5) |  | 422 | 52.0(35.2~68.7) | 0.20 |
| Stroke | 110 | 37.9(28.4~47.4) |  | 114 | 36.0(25.0~46.9) |  | 555 | 48.2(42.1~54.3) |  | 1159 | 43.0(38.3~47.8) |  | 1592 | 45.1(41.4~48.8) | 0.38 |
| COPD | 244 | 66.8(58.3~75.3) |  | 477 | 59.4(52.8~66.1) |  | 782 | 48.3(42.0~54.6) |  | 1533 | 50.0(45.5~54.6) |  | 1734 | 48.3(43.0~53.7) | <0.001 |
| **Rural** |  |  |  |  |  |  |  |  |  |  |  |  |  |  |  |
| At least one NCD | 1749 | 53.7(50.1~57.4) |  | 4108 | 50.2(47.9~52.5) |  | 8027 | 48.7(46.6~50.8) |  | 9362 | 51.8(49.7~53.9) |  | 10262 | 52.3(49.7~54.9) | 0.99 |
| Hypertension | 1106 | 51.1(45.8~56.5) |  | 2907 | 50.4(48.0~52.7) |  | 5726 | 48.3(46.0~50.6) |  | 5669 | 48.8(46.5~51.2) |  | 6214 | 52.3(48.8~55.9) | 0.81 |
| Diabetes | 157 | 50.1(41.3~58.9) |  | 726 | 41.7(37.6~45.7) |  | 1599 | 42.8(38.8~46.8) |  | 1220 | 50.5(45.4~55.6) |  | 1843 | 47.4(42.9~51.9) | 0.30 |
| MI | 62 | 40.4(20.9~59.8) |  | 130 | 50.5(40.2~60.8) |  | 299 | 38.6(31.6~45.5) |  | 327 | 36.3(28.8~43.8) |  | 403 | 40.0(32.0~48.1) | 0.66 |
| Stroke | 131 | 48.3(38.5~58.1) |  | 146 | 51.1(42.2~60.1) |  | 693 | 38.8(32.4~45.2) |  | 1431 | 44.6(40.3~48.8) |  | 1876 | 50.0(40.4~59.7) | 0.47 |
| COPD | 541 | 55.7(50.0~61.4) |  | 799 | 56.5(51.3~61.6) |  | 1270 | 53.3(48.1~58.5) |  | 2765 | 58.0(54.0~61.9) |  | 2727 | 53.6(49.5~57.6) | 0.53 |
| **Former smoking** |  |  |  |  |  |  |  |  |  |  |  |  |  |  |  |
| **Total** |  |  |  |  |  |  |  |  |  |  |  |  |  |  |  |
| At least one NCD | 3195 | 18.2(15.5~21.0) |  | 7370 | 16.4(15.0~17.7) |  | 14317 | 16.1(14.9~17.4) |  | 17056 | 18.4(17.4~19.5) |  | 19668 | 16.0(15.0~17.0) | 0.56 |
| Hypertension | 2144 | 18.5(15.5~21.6) |  | 5168 | 16.7(15.2~18.2) |  | 10140 | 15.9(14.5~17.2) |  | 10854 | 18.9(17.7~20.1) |  | 12283 | 15.6(14.3~16.8) | 0.31 |
| Diabetes | 440 | 18.8(15.2~22.4) |  | 1656 | 14.4(11.8~16.9) |  | 3383 | 15.8(13.5~18.2) |  | 2943 | 19.5(17.3~21.8) |  | 4515 | 14.9(13.0~16.8) | 0.35 |
| MI | 128 | 31.5(15.7~47.4) |  | 217 | 18.2(12.1~24.3) |  | 573 | 31.2(25.4~37.1) |  | 665 | 34.8(29.4~40.3) |  | 825 | 23.2(17.0~29.4) | 0.006 |
| Stroke | 239 | 32.0(25.2~38.7) |  | 260 | 29.4(23.1~35.7) |  | 1248 | 29.7(24.7~34.6) |  | 2590 | 27.8(25.2~30.3) |  | 3468 | 22.5(19.0~26.0) | 0.99 |
| COPD | 779 | 19.2(15.6~22.8) |  | 1276 | 20.3(17.4~23.1) |  | 2052 | 21.6(18.8~24.4) |  | 4298 | 18.7(16.5~20.9) |  | 4461 | 18.3(16.0~20.6) | 0.95 |
| **Urban** |  |  |  |  |  |  |  |  |  |  |  |  |  |  |  |
| At least one NCD | 1454 | 19.3(15.4~23.1) |  | 3262 | 16.0(13.7~18.2) |  | 6290 | 14.7(13.2~16.2) |  | 7694 | 17.8(16.6~19.0) |  | 9406 | 16.4(14.8~18.1) | 0.53 |
| Hypertension | 1041 | 21.6(17.2~26.1) |  | 2261 | 17.6(15.1~20.2) |  | 4414 | 15.3(13.6~17.0) |  | 5185 | 17.9(16.3~19.4) |  | 6069 | 16.4(14.8~18.1) | 0.10 |
| Diabetes | 284 | 18.0(13.4~22.6) |  | 930 | 12.8(9.5~16.1) |  | 1784 | 13.5(11.1~16.0) |  | 1723 | 18.4(15.6~21.2) |  | 2672 | 15.2(12.4~17.9) | 0.90 |
| MI | 66 | 36.4(15.3~57.6) |  | 87 | 23.3(12.9~33.7) |  | 274 | 32.4(24.2~40.7) |  | 338 | 31.8(24.8~38.8) |  | 422 | 19.1(10.7~27.4) | 0.02 |
| Stroke | 109 | 34.0(22.4~45.6) |  | 114 | 40.8(32.8~48.7) |  | 555 | 27.7(19.7~35.7) |  | 1159 | 26.6(23.3~30.0) |  | 1592 | 23.3(19.8~26.8) | 0.37 |
| COPD | 244 | 14.4(10.0~18.8) |  | 477 | 19.4(15.0~23.9) |  | 782 | 21.5(16.7~26.2) |  | 1533 | 20.5(16.8~24.2) |  | 1734 | 18.4(13.7~23.1) | 0.20 |
| **Rural** |  |  |  |  |  |  |  |  |  |  |  |  |  |  |  |
| At least one NCD | 1741 | 17.4(13.7~21.2) |  | 4108 | 16.7(15.0~18.3) |  | 8027 | 17.0(15.3~18.8) |  | 9362 | 18.9(17.3~20.4) |  | 10262 | 15.6(14.3~16.9) | 0.82 |
| Hypertension | 1103 | 16.1(12.5~19.6) |  | 2907 | 16.1(14.3~17.9) |  | 5726 | 16.2(14.4~18.1) |  | 5669 | 19.7(17.9~21.6) |  | 6214 | 14.8(13.0~16.6) | 0.89 |
| Diabetes | 156 | 19.7(14.0~25.5) |  | 726 | 16.1(12.5~19.8) |  | 1599 | 18.0(14.3~21.7) |  | 1220 | 20.8(17.2~24.4) |  | 1843 | 14.6(11.9~17.2) | 0.19 |
| MI | 62 | 26.6(3.5~49.8) |  | 130 | 15.1(7.7~22.5) |  | 299 | 30.5(22.4~38.5) |  | 327 | 37.6(29.5~45.7) |  | 403 | 28.0(20.8~35.2) | 0.11 |
| Stroke | 130 | 30.2(22.5~37.9) |  | 146 | 20.6(13.3~28.0) |  | 693 | 30.7(24.3~37.1) |  | 1431 | 28.5(24.9~32.1) |  | 1876 | 21.9(16.8~27.1) | 0.37 |
| COPD | 535 | 21.7(16.7~26.6) |  | 799 | 20.8(17.2~24.3) |  | 1270 | 21.6(18.2~25.1) |  | 2765 | 17.9(15.2~20.6) |  | 2727 | 18.3(15.9~20.7) | 0.46 |

CI, confidence interval; N, the number of subjects self-reported the specific disease; COPD, Chronic obstructive pulmonary disease; NCD, noncommunicable disease; MI, myocardial infarction. NCD refer to any of hypertension, diabetes, myocardial infarction, stroke, and COPD. Percentages and 95% CI are weighted.

# **Table O. Mean age of participants with at least one NCD or specific NCD across five CCDRFS.**

|  | **2007** | **2010** | **2013** | **2015** | **2018** |
| --- | --- | --- | --- | --- | --- |
| **Total** |  |  |  |  |  |
| At least one NCD | 53.8 | 52.4 | 55.6 | 56.9 | 58.3 |
| Hypertension | 54.1 | 52.9 | 56.0 | 57.4 | 58.7 |
| Diabetes | 54.0 | 51.9 | 54.8 | 57.4 | 58.4 |
| MI | 55.6 | 54.4 | 58.1 | 59.2 | 60.0 |
| Stroke | 57.1 | 56.3 | 59.1 | 60.1 | 60.7 |
| COPD | 54.0 | 53.3 | 56.5 | 56.2 | 58.0 |
| **Urban** |  |  |  |  |  |
| At least one NCD | 54.4 | 52.8 | 55.9 | 57.3 | 58.7 |
| Hypertension | 54.8 | 53.6 | 56.3 | 57.6 | 59.0 |
| Diabetes | 54.4 | 52.2 | 55.5 | 58.1 | 59.0 |
| MI | 58.2 | 57.2 | 58.3 | 59.5 | 60.3 |
| Stroke | 58.7 | 57.0 | 59.4 | 60.4 | 60.7 |
| COPD | 54.3 | 53.6 | 56.8 | 56.9 | 58.5 |
| **Rural** |  |  |  |  |  |
| At least one NCD | 53.3 | 52.1 | 55.3 | 56.6 | 58.0 |
| Hypertension | 53.4 | 52.4 | 55.7 | 57.1 | 58.5 |
| Diabetes | 53.3 | 51.5 | 54.1 | 56.4 | 57.5 |
| MI | 52.7 | 52.5 | 57.9 | 58.9 | 59.6 |
| Stroke | 55.7 | 55.8 | 58.9 | 60.0 | 60.7 |
| COPD | 53.9 | 53.1 | 56.4 | 55.8 | 57.6 |

CCDRFS, China Chronic Disease and Risk Factor Surveillance; COPD, chronic obstructive pulmonary disease; NCD, noncommunicable disease; MI, myocardial infarction.

# **Table P. Age first started daily smoking among current regular smokers among men and women in China, 2007-2018. Values are weighted means (95%CI).**

|  | | **Men** | | | | | | |  | **Women** | | | | | | |
| --- | --- | --- | --- | --- | --- | --- | --- | --- | --- | --- | --- | --- | --- | --- | --- | --- |
|  | | **2007** | **2010** | **2013** | **2015** | **2018** | **Annual rate of change** | ***p* for trend** |  | **2007** | **2010** | **2013** | **2015** | **2018** | **Annual rate of change** | ***p* for trend** |
| **Overall** | | 20.7(20.4~20.9) | 20.2(20.0~20.4) | 20.0(19.8~20.2) | 19.7(19.6~19.9) | 19.7(19.6~19.9) | -0.4 | <0.001 |  | 26.4(24.1~28.7) | 25.9(24.7~27.1) | 25.1(24.2~25.9) | 24.6(23.7~25.4) | 24.6(23.8~25.4) | -0.6 | 0.11 |
| **Age~ y** | |  |  |  |  |  |  |  |  |  |  |  |  |  |  |  |
|  | 18-19 | 16.4 (15.7~17.2) | 16.2 (15.9~16.5) | 16.0 (15.7~16.3) | 16.7 (16.4~17.0) | 15.7 (15.3~16.1) | -0.2 | 0.45 |  | 16.0 (13.4~18.6) | 16.6 (16.4~16.9) | 17.4 (16.1~18.6) | 16.0 (16.0~16.0) | - | 0.3 | 0.64 |
|  | 20-24 | 18.4 (18.1~18.7) | 17.7 (17.5~17.9) | 17.6 (17.3~17.8) | 17.7 (17.5~18.0) | 17.4 (16.9~17.9) | -0.4 | 0.006 |  | 21.3 (19.6~22.9) | 18.1 (16.7~19.5) | 17.4 (16.4~18.4) | 19.1 (16.1~22.1) | 18.8 (17.4~20.1) | -0.8 | 0.25 |
|  | 25-29 | 19.2 (18.8~19.5) | 18.8 (18.6~19.1) | 18.6 (18.4~18.8) | 18.5 (18.3~18.7) | 18.2 (17.9~18.6) | -0.5 | ＜0.001 |  | 20.8 (19.5~22.2) | 19.8 (18.9~20.7) | 19.2 (18.0~20.5) | 20.5 (18.6~22.3) | 21.9 (20.2~23.5) | 0.5 | 0.15 |
|  | 30-39 | 20.1 (19.8~20.5) | 19.8 (19.5~20.0) | 19.6 (19.4~19.8) | 19.6 (19.3~19.8) | 19.4 (19.2~19.7) | -0.3 | 0.002 |  | 22.9 (20.9~24.9) | 22.2 (21.3~23.2) | 22.3 (21.0~23.6) | 22.0 (20.8~23.3) | 22.4 (21.2~23.7) | -0.2 | 0.62 |
|  | 40-49 | 21.0 (20.7~21.2) | 20.5 (20.3~20.8) | 20.3 (20.1~20.5) | 20.1 (19.9~20.2) | 20.3 (20.1~20.5) | -0.3 | <0.001 |  | 25.8 (23.0~28.5) | 26.6 (25.3~28.0) | 24.4 (23.3~25.5) | 24.6 (23.3~25.9) | 25.2 (23.9~26.4) | -0.5 | 0.38 |
|  | 50-59 | 21.9 (21.5~22.2) | 21.5 (21.2~21.8) | 21.2 (21.0~21.4) | 20.8 (20.5~21.0) | 20.9 (20.6~21.1) | -0.5 | <0.001 |  | 26.8 (24.4~29.1) | 28.4 (26.6~30.2) | 26.9 (25.7~28.2) | 25.6 (24.3~26.9) | 25.9 (24.7~27.1) | -0.6 | 0.23 |
|  | 60-69 | 23.4 (22.7~24.0) | 23.0 (22.6~23.4) | 22.6(22.2~22.9) | 21.7 (21.4~22.0) | 21.8 (21.5~22.1) | -0.7 | <0.001 |  | 31.8 (28.6~35.1) | 29.0 (26.8~31.2) | 28.8 (27.5~30.1) | 26.9 (25.0~28.7) | 27.7 (25.9~29.4) | -1.3 | 0.03 |
| **Areas** | |  |  |  |  |  |  |  |  |  |  |  |  |  |  |  |
|  | Urban | 20.3 (19.9~20.8) | 20.1 (19.8~20.5) | 19.9 (19.6~20.2) | 19.8 (19.6~20.0) | 19.7 (19.4~19.9) | -0.3 | 0.01 |  | 27.3 (25.1~29.4) | 26.1 (24.6~27.5) | 25.0 (23.6~26.5) | 25.6 (24.2~26.9) | 25.2 (23.9~26.5) | -0.7 | 0.09 |
|  | Rural | 20.9 (20.6~21.1) | 20.3 (20.0~20.5) | 20.0 (19.8~20.2) | 19.7 (19.5~19.9) | 19.8 (19.5~20.0) | -0.5 | <0.001 |  | 25.9 (22.8~29.1) | 25.8 (24.1~27.5) | 25.1 (24.0~26.2) | 24.0 (23.0~25.1) | 24.2 (23.1~25.3) | -0.8 | 0.18 |
| **Education** | |  |  |  |  |  |  |  |  |  |  |  |  |  |  |  |
|  | No formal/primary school | 20.9 (20.4~21.3) | 20.3 (20.0~20.6) | 20.0 (19.8~20.3) | 19.6 (19.3~19.8) | 19.5 (19.2~19.9) | -0.6 | <0.001 |  | 26.8 (23.8~29.9) | 26.4 (24.7~28.1) | 25.4 (24.3~26.4) | 23.8 (22.6~25.0) | 24.8 (23.5~26.1) | -0.9 | 0.06 |
|  | Secondary school | 20.3 (20.0~20.5) | 20.0 (19.7~20.2) | 19.8 (19.6~20.0) | 19.5 (19.3~19.7) | 19.7 (19.5~19.9) | -0.3 | <0.001 |  | 25.7 (23.5~27.9) | 25.1 (23.8~26.5) | 24.8 (23.6~26.0) | 26.4 (25.4~27.5) | 25.7 (24.6~26.8) | 0.2 | 0.60 |
|  | High school | 21.0 (20.7~21.4) | 20.4 (20.1~20.6) | 20.3 (20.1~20.5) | 20.2 (19.9~20.4) | 20.0 (19.7~20.3) | -0.4 | 0.002 |  | 25.2 (23.0~27.4) | 25.1 (22.7~27.5) | 27.3 (25.2~29.3) | 26.5 (24.6~28.5) | 24.0 (22.1~26.0) | -0.1 | 0.21 |
|  | College/university | 21.5 (21.1~21.9) | 20.6 (20.4~20.9) | 20.4 (20.0~20.7) | 20.4 (20.1~20.7) | 20.0 (19.8~20.2) | -0.6 | <0.001 |  | 20.7 (16.1~25.2) | 24.2 (21.1~27.3) | 20.1 (17.1~23.1) | 21.9 (19.7~24.1) | 21.6 (20.1~23.1) | -0.1 | 0.14 |
| **Occupation** | |  |  |  |  |  |  |  |  |  |  |  |  |  |  |  |
|  | Agriculture | 20.9 (20.6~21.3) | 20.4 (20.1~20.6) | 20.1 (19.9~20.3) | 19.9 (19.7~20.1) | 20.0 (19.7~20.2) | -0.4 | <0.001 |  | 25.3 (22.3~28.3) | 25.7 (23.8~27.5) | 24.4 (23.3~25.5) | 24.1 (23.1~25.2) | 24.2 (23.1~25.3) | -0.6 | 0.24 |
|  | Manufacture | 20.1 (19.7~20.6) | 19.7 (19.3~20.0) | 19.8 (19.4~20.2) | 19.4 (19.0~19.8) | 19.5 (19.1~19.8) | -0.3 | 0.001 |  | 29.5 (24.1~35.0) | 22.5 (19.1~25.8) | 22.4 (20.2~24.5) | 21.8 (20.0~23.6) | 28.3 (22.5~34.1) | -0.5 | 0.65 |
|  | Service provider | 19.9 (19.4~20.3) | 19.7 (19.3~20.0) | 19.3 (18.9~19.6) | 19.5 (19.2~19.7) | 19.6 (19.2~20.0) | -0.2 | 0.32 |  | 24.7 (21.4~28.1) | 23.0 (20.9~25.2) | 22.2 (20.4~24.0) | 23.9 (21.6~26.3) | 23.0 (20.8~25.3) | -0.4 | 0.85 |
|  | Managers/professionals | 21.0 (20.6~21.4) | 20.5 (20.2~20.8) | 20.3 (20.0~20.6) | 20.0 (19.7~20.2) | 19.9 (19.6~20.2) | -0.5 | <0.001 |  | 23.8 (22.5~25.1) | 24.2 (20.3~28.1) | 23.4 (19.5~27.3) | 23.1 (20.4~25.8) | 24.7 (22.3~27.1) | 0.1 | 0.56 |
|  | Others | 20.6 (20.1~21.0) | 20.0 (19.7~20.3) | 19.8 (19.5~20.0) | 19.7 (19.4~19.9) | 19.4 (19.1~19.8) | -0.5 | <0.001 |  | 25.4 (22.7~28.1) | 22.5 (20.5~24.5) | 23.8 (20.2~27.5) | 25.3 (22.1~28.4) | 25.7 (22.1~29.4) | 0.5 | 0.57 |
|  | Unemployed/students | 19.2 (18.7~19.8) | 19.3 (18.9~19.8) | 19.2 (18.8~19.5) | 19.0 (18.7~19.3) | 19.1 (18.7~19.5) | -0.1 | 0.72 |  | 29.0 (25.9~32.0) | 27.0 (25.8~28.3) | 25.8 (24.7~26.9) | 24.4 (22.9~26.0) | 24.3 (22.8~25.7) | -1.7 | 0.001 |
|  | Retired | 22.6 (22.0~23.2) | 22.2 (21.7~22.8) | 22.2 (21.7~22.7) | 21.2 (20.8~21.5) | 21.0 (20.6~21.4) | -0.7 | <0.001 |  | 32.4 (29.2~35.7) | 30.9 (28.8~33.1) | 31.9 (29.3~34.5) | 30.9 (28.8~32.9) | 29.1 (27.5~30.7) | -0.8 | 0.17 |

CI, confidence interval.

# **Table Q. Age first started daily smoking among current regular smokers by sex, area, and year of birth. Values are weighted means (95%CI).**

|  | **Men** | | |  | **Women** | | |
| --- | --- | --- | --- | --- | --- | --- | --- |
|  | **Overall** | **Urban** | **Rural** |  | **Overall** | **Urban** | **Rural** |
| 1930-40s | 23.1  (22.8~23.4) | 22.9  (22.4~23.4) | 23.2  (22.8~23.6) |  | 29.8  (28.1~31.6) | 30.5  (28.4~32.5) | 29.5  (27.2~31.8) |
| 1950s | 21.5  (21.3~21.7) | 21.2  (20.9~21.5) | 21.7  (21.4~21.9) |  | 26.9  (25.5~28.2) | 29.0  (27.7~30.3) | 25.9  (24.2~27.6) |
| 1960s | 20.6  (20.5~20.8) | 20.5  (20.3~20.8) | 20.7  (20.5~20.9) |  | 26.0  (25.0~26.9) | 27.1  (26.1~28.2) | 25.4  (24.1~26.6) |
| 1970s | 19.9  (19.8~20.1) | 19.9  (19.7~20.1) | 20.0  (19.8~20.1) |  | 23.6  (22.9~24.2) | 24.3  (23.4~25.2) | 23.0  (22.2~23.8) |
| 1980s | 18.8  (18.7~18.9) | 19.0  (18.7~19.2) | 18.7  (18.6~18.9) |  | 20.7  (20.0~21.3) | 21.1  (20.2~22.1) | 20.1  (19.3~21.0) |
| 1990s | 17.4  (17.2~17.6) | 17.5  (17.3~17.7) | 17.4  (17.1~17.6) |  | 18.4  (17.6~19.3) | 18.7  (17.6~19.7) | 18.1  (16.7~19.5) |
| *p* value | <0.0001 | <0.0001 | <0.0001 |  | <0.0001 | <0.0001 | <0.0001 |

CI, confidence interval.

# **Table R. Percentages of manufactured cigarette smokers among current regular smokers among men and women in China, 2007-2018. Values are weighted percentages (95%CI).**

|  | | **Men** | | | | | | |  | **Women** | | | | | | | |
| --- | --- | --- | --- | --- | --- | --- | --- | --- | --- | --- | --- | --- | --- | --- | --- | --- | --- |
|  | | **2007** | **2010** | **2013** | **2015** | **2018** | **Annual rate of change** | ***p* for trend** |  | **2007** | **2010** | **2013** | **2015** | **2018** | **Annual rate of change** | ***p* for trend** |  |
| **Overall** | | 95.0(93.7~96.2) | 93.1(91.5~94.6) | 97.9(97.2~98.7) | 96.3(94.9~97.6) | 97.7(97.0~98.4) | 0.3 | ＜0.001 |  | 79.5(69.5~89.4) | 82.9(76.4~89.5) | 95.8(93.6~98.0) | 90.0(84.8~95.2) | 95.4(93.2~97.7) | 1.7 | ＜0.001 |  |
| **Age~ y** | |  |  |  |  |  |  |  |  |  |  |  |  |  |  |  |  |
|  | 18-19 | 99.6(98.7~100.0) | 98.9(97.4~100.0) | 100.0(100.0~100.0) | 96.9(93.7~100.0) | 99.1(97.6~100.0) | -0.1 | 0.26 |  | - | 89.4(67.5~100.0) | 100.0(100.0~100.0) | 100.0(100.0~100.0) | 100.0(100.0~100.0) | 1.3 | <0.001 |  |
|  | 20-24 | 98.0(95.9~100.0) | 98.4(97.5~99.3) | 99.8(99.5~100.0) | 99.5(99.0~99.9) | 98.1(95.9~100.0) | 0.1 | 0.83 |  | 97.1(92.4~100.0) | 74.7(45.2~100.0) | 100.0(100.0~100.0) | 100.0(100.0~100.0) | 100.0(100.0~100.0) | 1.3 | 0.03 |  |
|  | 25-29 | 98.9(98.0~99.7) | 98.1(97.0~99.1) | 99.1(98.2~100.0) | 97.6(96.4~98.9) | 98.6(97.1~100.0) | 0.0 | 0.58 |  | 96.2(89.5~100.0) | 95.4(90.3~100.0) | 96.5(90.8~100.0) | 98.6(96.0~100.0) | 93.2(82.1~100.0) | -0.1 | 0.78 |  |
|  | 30-39 | 97.1(96.0~98.1) | 96.9(95.6~98.1) | 98.8(98.1~99.6) | 98.5(97.9~99.1) | 98.8(98.1~99.6) | 0.2 | 0.001 |  | 86.2(74.9~97.5) | 89.1(82.9~95.2) | 97.6(95.1~100.0) | 94.2(85.0~100.0) | 98.6(96.5~100.0) | 1.2 | 0.06 |  |
|  | 40-49 | 95.9(94.3~97.6) | 94.7(92.9~96.4) | 98.6(97.6~99.5) | 96.7(94.5~98.9) | 98.7(98.1~99.3) | 0.3 | 0.01 |  | 75.8(64.6~87.0) | 86.6(78.8~94.4) | 95.2(91.0~99.5) | 91.7(84.5~98.9) | 97.7(95.5~99.8) | 2.2 | ＜0.001 |  |
|  | 50-59 | 92.1(90.0~94.2) | 87.0(84.1~89.9) | 96.9(95.7~98.2) | 93.9(91.8~96.1) | 97.0(95.9~98.1) | 0.7 | <0.001 |  | 73.7(62.9~84.4) | 79.5(70.0~89.1) | 94.5(91.4~97.7) | 87.5(83.0~92.0) | 92.9(89.2~96.6) | 2.2^*^ | 0.002 |  |
|  | 60-69 | 84.3(80.5~88.2) | 79.4(75.5~83.4) | 92.6(90.2~94.9) | 89.0(85.8~92.2) | 92.9(90.7~95.0) | 1.2 | <0.001 |  | 78.2(67.2~89.3) | 75.8(66.6~85.0) | 95.1(92.7~97.4) | 85.1(77.3~93.0) | 90.2(85.7~94.7) | 1.6^*^ | 0.01 |  |
| **Areas** | |  |  |  |  |  |  |  |  |  |  |  |  |  |  |  |  |
|  | Urban | 98.1(97.3~98.9) | 96.4(94.8~98.0) | 98.7(98.1~99.2) | 98.5(97.9~99.0) | 98.7(98.0~99.4) | 0.1 | 0.04 |  | 93.9(89.0~98.7) | 91.4(85.4~97.5) | 98.4(96.6~100.0) | 97.7(95.9~99.4) | 98.5(96.5~100.0) | 0.6 | 0.01 |  |
|  | Rural | 93.1(91.3~94.9) | 91.2(89.0~93.3) | 97.6(96.5~98.6) | 94.9(92.9~97.0) | 97.0(96.0~98.1) | 0.5 | 0.002 |  | 72.1(59.6~84.6) | 77.8(69.1~86.4) | 93.9(90.4~97.4) | 86.0(78.4~93.7) | 93.0(89.5~96.5) | 2.4 | 0.001 |  |
| **Education** | | |  |  |  |  |  |  |  |  |  |  |  |  |  |  |  |
|  | No formal/primary | 90.4(88.2~92.7) | 85.0(81.9~88.1) | 95.5(94.0~97.1) | 93.0(90.9~95.2) | 96.1(94.9~97.2) | 0.8 | <0.001 |  | 70.4(59.5~81.2) | 75.9(67.6~84.2) | 93.9(90.9~96.9) | 86.3(79.8~92.8) | 92.4(88.6~96.2) | 2.6 | ＜0.001 |  |
|  | Secondary | 96.9(95.6~98.2) | 95.4(94.2~96.5) | 98.8(98.3~99.4) | 97.0(95.8~98.3) | 97.8(96.9~98.8) | 0.1 | 0.08 |  | 91.1(82.5~99.6) | 91.4(85.0~97.8) | 97.8(95.7~99.9) | 95.4(90.8~100.0) | 98.7(97.6~99.9) | 0.8 | 0.01 |  |
|  | High | 97.4(96.1~98.6) | 97.7(96.8~98.6) | 98.9(98.1~99.6) | 97.9(96.5~99.2) | 98.8(97.8~99.8) | 0.1 | 0.25 |  | 97.8(94.5~100.0) | 96.7(92.6~100.0) | 98.8(97.5~100.0) | 97.5(95.4~99.6) | 99.2(98.3~100.0) | 0.1 | 0.20 |  |
|  | College/university | 99.7(99.3~100.0) | 99.0(98.2~99.9) | 99.7(99.4~100.0) | 99.2(98.7~99.6) | 99.3(98.5~100.0) | 0.0 | 0.49 |  | 100.0(100.0~100.0) | 99.0(97.0~100.0) | 98.9(97.2~100.0) | 97.3(92.2~100.0) | 100.0(100.0~100.0) | -0.1 | 0.55 |  |
| **Occupation** | |  |  |  |  |  |  |  |  |  |  |  |  |  |  |  |  |
|  | Agriculture | 92.2(90.2~94.2) | 88.6(86.2~91.0) | 97.1(96.1~98.2) | 94.1(91.8~96.5) | 96.2(94.8~97.6) | 0.6 | ＜0.001 |  | 67.5(55.2~79.8) | 75.5(67.2~83.8) | 93.7(90.8~96.6) | 88.3(83.8~92.8) | 92.9(89.6~96.2) | 3.1 | <0.001 |  |
|  | Manufacture | 98.6(97.9~99.4) | 98.8(98.0~99.5) | 98.5(97.4~99.7) | 97.0(95.0~99.0) | 98.5(96.3~100.0) | -0.1 | 0.47 |  | 83.4(55.7~100.0) | 95.6(87.3~100.0) | 100.0(100.0~100.0) | 96.5(90.0~100.0) | 100.0(100.0~100.0) | 1.4 | 0.25 |  |
|  | Service provider | 97.5(94.8~100.0) | 99.1(98.5~99.6) | 99.6(98.9~100.0) | 99.0(98.4~99.6) | 98.4(95.8~100.0) | 0.1 | 0.74 |  | 98.8(96.6~100.0) | 82.3(55.7~100.0) | 99.6(99.0~100.0) | 97.5(93.6~100.0) | 100.0(100.0~100.0) | 0.7 | 0.04 |  |
|  | Manager/professionals | 99.4(98.9~99.8) | 98.2(97.3~99.2) | 99.5(99.2~99.8) | 98.1(97.1~99.0) | 99.0(98.2~99.7) | 0.0 | 0.48 |  | 100.0(100.0~100.0) | 99.5(98.5~100.0) | 99.3(97.9~100.0) | 97.5(94.5~100.0) | 97.7(94.7~100.0) | 0.2 | 0.05 |  |
|  | Others | 95.9(94.0~97.7) | 96.5(95.2~97.9) | 98.6(97.8~99.4) | 97.5(96.5~98.5) | 98.6(97.9~99.3) | 0.3 | 0.001 |  | 91.3(83.2~99.5) | 96.3(90.9~100.0) | 96.4(92.1~100.0) | 85.1(65.5~100.0) | 98.5(96.1~100.0) | 0.2 | 0.33 |  |
|  | Unemployed/students | 96.6(94.7~98.6) | 93.7(91.4~95.9) | 96.9(95.0~98.9) | 96.3(94.9~97.6) | 98.7(98.2~99.3) | 0.3 | ＜0.001 |  | 92.4(85.9~98.8) | 87.8(81.7~93.9) | 96.9(94.2~99.5) | 88.0(77.4~98.6) | 96.1(92.2~100.0) | 0.3 | 0.40 |  |
|  | Retired | 96.1(92.5~99.7) | 94.7(92.4~96.9) | 98.7(97.4~100.0) | 98.3(97.3~99.3) | 99.0(98.5~99.6) | 0.4 | 0.007 |  | 98.3(95.2~100.0) | 96.4(91.1~100.0) | 99.4(98.4~100.0) | 97.5(94.7~100.0) | 98.9(97.1~100.0) | 0.1 | 0.86 |  |

CI, confidence interval.

# **Table S. Percentages of manufactured cigarette smokers among current regular smokers by sex, area, and year of birth. Values are weighted percentages (95%CI).**

|  | **Men** | | |  | **Women** | | |
| --- | --- | --- | --- | --- | --- | --- | --- |
|  | **Overall** | **Urban** | **Rural** |  | **Overall** | **Urban** | **Rural** |
| 1930-40s | 84.9  (82.3~87.4) | 93.0  (91.1~94.9) | 81.4  (77.9~84.8) |  | 81.5  (75.1~87.9) | 92.5  (88.7~96.2) | 76.4  (68.2~84.6) |
| 1950s | 92.0  (90.4~93.5) | 96.1  (94.9~97.2) | 89.7  (87.4~92.0) |  | 84.5  (79.2~89.8) | 93.2  (89.9~96.6) | 80.6  (73.7~87.4) |
| 1960s | 96.2  (95.1~97.3) | 98.3  (97.5~99.0) | 95.1  (93.4~96.7) |  | 88.1  (82.3~93.9) | 97.3  (94.9~99.6) | 83.0  (75.4~90.6) |
| 1970s | 97.8  (97.1~98.5) | 99.0  (98.5~99.4) | 97.2  (96.1~98.2) |  | 94.4  (91.4~97.4) | 98.6  (97.0~100.0) | 91.2  (86.3~96.2) |
| 1980s | 98.7  (98.3~99.1) | 99.1  (98.5~99.7) | 98.4  (97.9~99.0) |  | 92.7  (86.9~98.5) | 95.6  (89.5~100.0) | 90.1  (80.7~99.5) |
| 1990s | 98.8  (98.2~99.4) | 99.0  (97.9~100.0) | 98.6  (97.9~99.4) |  | 98.0  (95.4~100.0) | 98.1  (94.6~100.0) | 97.9  (93.9~100.0) |
| *p* value | <0.0001 | <0.0001 | <0.0001 |  | <0.0001 | 0.1525 | <0.0001 |

CI, confidence interval.

# **Table T. Daily number of cigarettes smoked among current regular smokers among men and women in China, 2007-2018. Values are weighted means (95%CI).**

|  | | **Men** | | | | | | |  | **Women** | | | | | | |
| --- | --- | --- | --- | --- | --- | --- | --- | --- | --- | --- | --- | --- | --- | --- | --- | --- |
|  | | **2007** | **2010** | **2013** | **2015** | **2018** | **Annual rate of change** | ***p* for trend** |  | **2007** | **2010** | **2013** | **2015** | **2018** | **Annual rate of change** | ***p* for trend** |
| **Overall** | | 17.9(17.5~18.4) | 17.9(17.4~18.3) | 18.0(17.6~18.3) | 17.3(16.9~17.6) | 17.2(16.8~17.5) | -0.4 | 0.005 |  | 13.7(12.9~14.5) | 13.3(12.6~14.1) | 13.3(12.7~13.9) | 13.7(12.9~14.5) | 14.4(11.6~17.2) | 0.5 | 0.57 |
| **Age~ y** | |  |  |  |  |  |  |  |  |  |  |  |  |  |  |  |
|  | 18-19 | 11.9(10.7~13.0) | 12.6(11.7~13.6) | 13.5(12.1~14.9) | 12.3(11.1~13.4) | 12.4(10.0~14.9) | 0.3 | 0.74 |  | - | 16.5(15.1~17.8) | 9.0(6.9~11.2) | 5.0(5.0~5.0) | 39.8(39.4~40.3) | 9.0 | <0.001 |
|  | 20-24 | 15.2(14.4~16.0) | 13.1(12.4~13.7) | 13.8(12.9~14.7) | 12.6(11.8~13.5) | 13.4(12.5~14.2) | -1.1 | 0.01 |  | 18.6(16.7~20.5) | 8.9(5.3~12.5) | 11.2(9.6~12.9) | 7.8(3.1~12.6) | 10.4(8.2~12.5) | -4.7 | ＜0.001 |
|  | 25-29 | 15.2(14.5~16.0) | 15.2(14.5~16.0) | 15.3(14.9~15.8) | 14.5(14.0~15.1) | 14.9(14.1~15.7) | -0.3 | 0.28 |  | 13.3(7.4~19.1) | 14.9(12.3~17.5) | 13.8(10.9~16.7) | 10.5(8.2~12.7) | 7.4(4.2~10.6) | -5.3 | 0.007 |
|  | 30-39 | 17.9(17.4~18.4) | 18.0(17.3~18.6) | 17.6(17.1~18.0) | 16.8(16.4~17.3) | 16.5(16.0~17.0) | -0.8 | <0.001 |  | 13.1(10.9~15.3) | 13.3(11.7~14.8) | 12.3(11.0~13.5) | 13.2(11.0~15.4) | 10.8(9.0~12.6) | -1.5 | 0.24 |
|  | 40-49 | 19.2(18.6~19.7) | 20.2(19.7~20.7) | 19.8(19.3~20.3) | 19.1(18.7~19.6) | 18.7(18.2~19.1) | -0.4 | 0.03 |  | 12.8(11.4~14.2) | 13.7(12.2~15.1) | 15.2(13.9~16.5) | 14.2(13.1~15.3) | 14.7(13.5~16.0) | 1.2 | 0.10 |
|  | 50-59 | 19.8(19.2~20.4) | 19.3(18.8~19.8) | 20.1(19.7~20.5) | 19.8(19.3~20.3) | 19.8(19.4~20.2) | 0.1 | 0.64 |  | 13.7(12.7~14.8) | 13.8(12.5~15.1) | 13.7(12.9~14.5) | 14.8(13.4~16.1) | 14.1(13.0~15.1) | 0.4 | 0.39 |
|  | 60-69 | 17.3(16.4~18.3) | 17.7(17.2~18.2) | 18.6(18.1~19.0) | 18.3(17.9~18.8) | 17.8(17.4~18.3) | 0.4 | 0.20 |  | 11.3(9.1~13.5) | 12.5(11.1~13.8) | 12.4(11.3~13.6) | 13.9(12.8~15.0) | 13.0(11.9~14.1) | 1.4 | 0.08 |
| **Areas** | |  |  |  |  |  |  |  |  |  |  |  |  |  |  |  |
|  | Urban | 17.5(16.9~18.1) | 16.8(16.1~17.5) | 17.3(16.8~17.8) | 16.4(15.9~16.8) | 16.4(16.0~16.9) | -0.6 | 0.01 |  | 14.4(12.7~16.1) | 13.8(12.6~15.0) | 12.7(12.0~13.5) | 13.4(12.1~14.8) | 15.3(9.5~21.1) | 0.3 | 0.86 |
|  | Rural | 18.2(17.6~18.8) | 18.5(18.0~19.0) | 18.3(17.8~18.7) | 17.8(17.3~18.2) | 17.7(17.1~18.2) | -0.3 | 0.10 |  | 13.2(11.3~15.2) | 13.0(12.0~14.0) | 13.7(12.9~14.5) | 13.9(12.8~14.9) | 13.7(12.8~14.5) | 0.5 | 0.42 |
| **Education** | | |  |  |  |  |  |  |  |  |  |  |  |  |  |  |
|  | No formal/primary school | 18.6(18.0~19.2) | 19.5(18.9~20.1) | 19.8(19.3~20.3) | 19.2(18.7~19.6) | 18.9(18.4~19.4) | 0.1 | 0.74 |  | 12.1(10.9~13.4) | 13.2(12.1~14.2) | 13.6(12.8~14.3) | 13.9(13.1~14.8) | 16.4(12.0~20.9) | 2.5 | 0.02 |
|  | Secondary school | 18.3(17.8~18.8) | 18.1(17.7~18.6) | 18.1(17.7~18.6) | 17.5(17.1~17.8) | 17.7(17.3~18.1) | -0.4 | 0.003 |  | 15.7(13.5~17.9) | 14.3(13.0~15.6) | 13.7(12.8~14.7) | 13.4(12.2~14.6) | 13.1(12.2~14.1) | -1.6 | 0.03 |
|  | High school | 16.9(16.3~17.5) | 16.4(15.9~16.9) | 16.3(15.8~16.8) | 15.9(15.4~16.4) | 16.1(15.5~16.7) | -0.5 | 0.15 |  | 14.8(11.8~17.7) | 12.5(11.2~13.7) | 12.0(10.4~13.7) | 13.5(10.9~16.2) | 12.3(9.6~14.9) | -1.2 | 0.33 |
|  | College/university | 14.6(13.8~15.4) | 14.6(13.9~15.2) | 14.1(13.5~14.7) | 13.6(13.0~14.3) | 13.2(12.5~13.9) | -1.0 | 0.05 |  | 13.3(6.0~20.5) | 10.6(7.4~13.8) | 10.8(9.2~12.5) | 13.0(6.3~19.7) | 9.1(7.0~11.2) | -2.2 | 0.63 |
| **Occupation** | |  |  |  |  |  |  |  |  |  |  |  |  |  |  |  |
|  | Agriculture | 18.4(17.7~19.0) | 19.1(18.6~19.6) | 18.8(18.3~19.2) | 18.6(18.1~19.1) | 18.2(17.8~18.7) | -0.2 | 0.36 |  | 12.4(10.8~13.9) | 12.5(11.6~13.3) | 13.7(12.8~14.6) | 13.9(12.8~14.9) | 13.3(12.4~14.2) | 0.1 | 0.14 |
|  | Manufacture | 18.0(17.1~18.9) | 16.7(15.8~17.5) | 17.5(16.9~18.2) | 16.7(15.8~17.5) | 17.3(16.4~18.2) | -0.3 | 0.12 |  | 12.5(9.9~15.1) | 18.2(13.2~23.1) | 12.6(9.8~15.5) | 18.1(12.0~24.2) | 13.3(8.0~18.6) | 0.3 | 0.87 |
|  | Service provider | 17.7(16.9~18.5) | 17.0(16.0~17.9) | 17.5(16.5~18.6) | 16.0(15.5~16.5) | 15.8(15.2~16.4) | -1.0 | <0.001 |  | 13.3(8.4~18.1) | 12.0(9.1~14.8) | 14.3(10.5~18.1) | 11.9(8.8~15.0) | 11.5(8.6~14.3) | -1.0 | 0.66 |
|  | Managers/professionals | 16.8(16.1~17.5) | 16.1(15.6~16.6) | 15.8(15.2~16.4) | 15.6(15.1~16.1) | 15.3(14.6~15.9) | -0.8 | 0.01 |  | 18.1(15.7~20.6) | 12.7(10.9~14.6) | 11.1(9.1~13.1) | 15.6(11.6~19.5) | 10.6(6.3~14.9) | -3.4 | 0.25 |
|  | Others | 17.3(16.5~18.1) | 17.6(16.7~18.4) | 17.8(17.2~18.5) | 17.3(16.9~17.8) | 17.4(16.8~18.0) | 0.0 | 0.70 |  | 16.0(12.9~19.1) | 15.1(13.2~17.0) | 12.0(9.8~14.2) | 14.2(12.2~16.2) | 11.0(9.6~12.4) | -3.1 | 0.003 |
|  | Unemployed/students | 18.0(17.0~19.0) | 16.7(15.8~17.5) | 17.9(16.9~18.9) | 16.1(15.3~17.0) | 16.9(15.8~17.9) | -0.6 | 0.20 |  | 14.3(11.5~17.0) | 14.3(13.1~15.6) | 13.3(12.2~14.4) | 12.7(11.5~13.9) | 18.7(11.0~26.3) | 1.6 | 0.34 |
|  | Retired | 17.6(16.5~18.8) | 17.4(16.6~18.1) | 17.0(16.2~17.7) | 17.0(16.5~17.6) | 16.9(16.2~17.7) | -0.4 | 0.22 |  | 13.1(10.8~15.4) | 14.2(12.5~15.8) | 13.5(12.2~14.8) | 15.0(12.7~17.2) | 12.9(11.5~14.4) | -0.1 | 0.82 |

CI, confidence interval.

# **Table U. Daily number of cigarettes smoked among current regular smokers by sex, residence, and year of birth. Values are weighted means (95%CI).**

|  | **Men** | | |  | **Women** | | |
| --- | --- | --- | --- | --- | --- | --- | --- |
|  | **Overall** | **Urban** | **Rural** |  | **Overall** | **Urban** | **Rural** |
| 1930-40s | 17.7  (17.3~18.1) | 17.6  (17.0~18.2) | 17.8  (17.3~18.3) |  | 12.2  (11.2~13.2) | 12.7  (11.6~13.7) | 12.0  (10.5~13.4) |
| 1950s | 19.2  (18.9~19.6) | 18.8  (18.3~19.3) | 19.5  (19.1~20.0) |  | 13.6  (13.0~14.1) | 13.9  (12.9~14.8) | 13.4  (12.7~14.1) |
| 1960s | 19.8  (19.5~20.1) | 18.8  (18.4~19.2) | 20.4  (20.0~20.8) |  | 14.0  (13.3~14.7) | 13.7  (12.9~14.6) | 14.1  (13.2~15.1) |
| 1970s | 18.2  (17.9~18.5) | 17.1  (16.7~17.5) | 18.8  (18.5~19.2) |  | 14.1  (13.4~14.7) | 13.9  (12.7~15.0) | 14.2  (13.5~15.0) |
| 1980s | 15.3  (15.0~15.6) | 14.6  (14.2~15.0) | 15.8  (15.4~16.2) |  | 13.2  (11.5~15.0) | 12.7  (10.6~14.7) | 13.8  (11.1~16.4) |
| 1990s | 13.2  (12.8~13.7) | 13.0  (12.2~13.8) | 13.4  (12.8~13.9) |  | 15.6  (6.9~24.3) | 18.2  (6.3~30.1) | 10.7  (7.6~13.8) |
| *p* value | <0.0001 | 0.0001 | 0.0150 |  | 0.4095 | 0.4739 | 0.5414 |

CI, confidence interval.

# **Table V. The prevalence of current smoking among adults aged ≥ 18 years by province in China in 2018*. Values are weighted percentages (95%CI).**

| **Province*** | **Overall** | **Men** | | |  | **Women** | | |
| --- | --- | --- | --- | --- | --- | --- | --- | --- |
|  |  | **Total** | **Urban** | **Rural** |  | **Total** | **Urban** | **Rural** |
| Yunnan | 35.7(32.8~38.5) | 68.0(63.0~73.1) | 68.4(61.5~75.2) | 67.8(63.0~72.7) |  | 1.3(0.8~1.9) | 1.7(0.5~2.8) | 1.1(0.9~1.4) |
| Guizhou | 32.8(29.9~35.6) | 63.4(57.6~69.1) | 57.0(53.5~60.6) | 66.9(60.0~73.7) |  | 1.4(0.8~2.1) | 2.2(1.1~3.4) | 1.0(0.3~1.7) |
| Hunan | 31.9(30.9~33.0) | 61.5(59.4~63.6) | 58.4(54.1~62.7) | 63.8(62.0~65.7) |  | 1.7(1.1~2.4) | 2.6(1.7~3.4) | 1.1(0.7~1.5) |
| Qinghai | 31.7(30.8~32.5) | 60.2(58.6~61.8) | 66.4(63.0~69.8) | 54.6(51.4~57.8) |  | 0.8(0.0~1.8) | 1.2(0.0~2.7) | 0.5(0.0~1.0) |
| Shanxi | 30.5(29.4~31.6) | 56.7(53.3~60.1) | 57.6(56.0~59.3) | 56.0(50.7~61.2) |  | 5.1(0.0~10.5) | 8.8(3.3~14.2) | 1.3(0.7~2.0) |
| Sichuan | 29.5(25.1~33.9) | 56.7(50.6~62.9) | 60.6(44.7~76.6) | 54.0(47.5~60.5) |  | 2.0(0.7~3.3) | 1.5(0.0~3.5) | 2.4(1.3~3.4) |
| Gansu | 28.9(25.9~31.8) | 56.7(50.3~63.2) | 57.7(42.0~73.5) | 56.1(54.5~57.7) |  | 0.4(0.1~0.7) | 0.2(0.0~0.8) | 0.5(0.4~0.6) |
| Henan | 28.3(22.3~34.3) | 54.8(47.4~62.1) | 57.8(45.7~70.0) | 52.1(46.4~57.9) |  | 0.6(0.2~1.1) | 0.8(0.2~1.3) | 0.5(0.0~1.1) |
| Shaanxi | 27.0(23.4~30.5) | 54.8(46.9~62.7) | 52.0(45.0~59.1) | 56.7(48.7~64.6) |  | 0.4(0.2~0.6) | 0.6(0.2~1.1) | 0.3(0.1~0.5) |
| Beijing | 29.9(24.4~35.4) | 53.4(46.0~60.8) | 53.6(45.3~62.0) | 51.7(49.8~53.7) |  | 5.4(4.2~6.6) | 5.8(5.0~6.7) | 2.8(2.1~3.4) |
| Chongqing | 27.2(22.6~31.8) | 52.8(46.3~59.2) | 49.0(37.2~60.8) | 57.4(51.4~63.4) |  | 1.9(1.3~2.4) | 1.8(0.8~2.7) | 2.0(1.7~2.2) |
| Hainan | 27.1(23.1~31.2) | 51.7(44.6~58.9) | 44.9(42.6~47.3) | 58.7(53.6~63.7) |  | 0.1(0.0~0.1) | 0.0(0.0~0.1) | 0.1(0.0~0.3) |
| Hubei | 26.6(22.0~31.1) | 50.7(46.4~55.1) | 48.7(39.8~57.5) | 52.8(47.3~58.3) |  | 2.0(0.5~3.5) | 1.9(0.2~3.6) | 2.1(0.2~3.9) |
| Jiangxi | 25.1(21.6~28.6) | 49.7(43.0~56.5) | 45.9(41.9~50.0) | 52.8(41.4~64.2) |  | 0.5(0.1~0.9) | 0.5(0.0~1.1) | 0.4(0.0~0.9) |
| Anhui | 25.0(23.7~26.3) | 49.1(46.2~52.1) | 46.4(39.0~53.9) | 51.3(47.1~55.5) |  | 1.2(0.9~1.5) | 1.2(0.7~1.6) | 1.2(0.7~1.7) |
| Tianjin | 27.4(23.6~31.3) | 48.7(42.7~54.7) | 47.7(41.0~54.3) | 53.3(49.5~57.1) |  | 3.2(2.0~4.3) | 2.9(1.6~4.1) | 4.5(0.9~8.0) |
| Guangdong | 25.4(21.2~29.6) | 48.2(38.6~57.7) | 44.2(35.8~52.7) | 57.3(43.3~71.3) |  | 1.4(0.5~2.3) | 1.5(0.3~2.8) | 1.0(0.0~2.1) |
| Guangxi | 24.6(21.3~27.9) | 47.5(40.7~54.3) | 46.9(41.9~51.9) | 48.0(38.3~57.7) |  | 0.7(0.1~1.3) | 1.4(0.4~2.4) | 0.2(0.0~0.4) |
| Jiangsu | 23.8(21.6~26.1) | 47.3(43.8~50.7) | 45.8(41.1~50.6) | 49.5(45.6~53.3) |  | 1.0(0.7~1.3) | 0.7(0.4~1.1) | 1.3(0.9~1.8) |
| Inner Mogonia | 26.4(24.1~28.7) | 46.7(42.6~50.8) | 38.6(31.0~46.2) | 56.1(51.8~60.4) |  | 5.3(4.0~6.7) | 2.1(1.4~2.9) | 9.5(5.0~14.1) |
| Liaoning | 25.9(22.8~29.0) | 45.5(40.4~50.5) | 38.7(31.6~45.7) | 57.0(51.4~62.6) |  | 6.5(4.3~8.7) | 3.3(1.6~4.9) | 12.0(9.5~14.6) |
| Xinjiang | 23.4(19.5~27.3) | 44.7(38.9~50.6) | 47.0(43.1~51.0) | 42.8(34.6~51.1) |  | 0.9(0.0~1.8) | 1.0(0.0~2.0) | 0.8(0.0~2.2) |
| Fujian | 23.0(18.3~27.7) | 44.4(36.2~52.7) | 35.7(27.1~44.4) | 56.3(48.3~64.3) |  | 1.0(0.2~1.9) | 1.1(0.3~2.0) | 0.9(0.0~2.0) |
| Heilongjiang | 25.6(19.8~31.3) | 43.7(32.9~54.4) | 40.3(20.7~59.8) | 48.0(45.8~50.2) |  | 7.0(4.1~9.9) | 4.7(2.5~6.8) | 10.1(3.7~16.4) |
| Zhejiang | 22.3(17.4~27.2) | 43.4(35.0~51.9) | 36.6(25.5~47.7) | 54.4(49.5~59.2) |  | 0.4(0.2~0.6) | 0.4(0.2~0.5) | 0.5(0.0~0.9) |
| Shandong | 21.8(19.9~23.7) | 43.1(38.5~47.7) | 42.2(36.9~47.5) | 44.1(39.2~48.9) |  | 0.8(0.3~1.4) | 0.5(0.1~0.9) | 1.1(0.3~2.0) |
| Jilin | 26.1(15.7~36.4) | 43.1(27.5~58.7) | 34.7(7.3~62.1) | 52.8(47.8~57.9) |  | 8.8(6.0~11.6) | 2.5(0.2~4.7) | 16.4(13.5~19.4) |
| Hebei | 23.6(21.1~26.0) | 42.9(40.2~45.6) | 38.5(33.4~43.6) | 46.4(42.1~50.6) |  | 4.4(3.2~5.7) | 2.8(0.1~5.6) | 5.7(5.0~6.4) |
| Tibet | 22.6(21.6~23.7) | 42.1(37.8~46.5) | 40.2(33.2~47.1) | 42.9(38.1~47.6) |  | 2.1(0.0~4.9) | 6.8(0.0~14.2) | 0.5(0.4~0.6) |
| Ningxia | 21.0(18.3~23.7) | 40.6(38.4~42.8) | 39.9(39.4~40.4) | 41.3(37.3~45.4) |  | 0.5(0.3~0.7) | 0.8(0.8~0.8) | 0.2(0.1~0.3) |
| Shanghai | 17.8(13.6~22.0) | 34.8(27.3~42.3) | 32.7(27.3~38.2) | 51.2(49.4~53.0) |  | 0.2(0.0~0.3) | 0.2(0.0~0.3) | 0.0 |

*Provinces are sorted by male current smoking rate from high to low.

# **Table W. The cigarettes production by province in China in 2018*.**

| **Province*** | **Cigarettes (billion)** | **Population in 2018 (thousand)** | **Cigarettes per capita** |
| --- | --- | --- | --- |
| Yunnan | 350,152 | 47,030 | 7,445 |
| Shanghai | 89,270 | 24,750 | 3,607 |
| Guizhou | 107,579 | 38,220 | 2,815 |
| Hunan | 163,556 | 66,350 | 2,465 |
| Hubei | 127,584 | 59,170 | 2,156 |
| Fujian | 85,514 | 41,040 | 2,084 |
| Jilin | 51,628 | 24,840 | 2,079 |
| Shaanxi | 79,439 | 39,310 | 2,021 |
| Gansu | 47,150 | 25,150 | 1,875 |
| Anhui | 111,441 | 60,760 | 1,834 |
| Chongqing | 52,000 | 31,630 | 1,644 |
| Henan | 152,536 | 98,640 | 1,546 |
| Jiangsu | 103,102 | 84,460 | 1,540 |
| Tianjin | 21,160 | 13,830 | 1,530 |
| Zhejiang | 93,947 | 62,730 | 1,498 |
| Guangxi | 70,005 | 49,470 | 1,415 |
| Jiangxi | 63,801 | 4,5130 | 1,414 |
| Shandong | 125,284 | 100,770 | 1,243 |
| Hainan | 12,000 | 9,820 | 1,222 |
| Heilongjiang | 37,950 | 33,270 | 1,140 |
| Ningxia | 8,000 | 7,100 | 1,127 |
| Inner Mogonia | 26,260 | 24,220 | 1,084 |
| Guangdong | 128,368 | 123,480 | 1,040 |
| Hebei | 76,200 | 74,260 | 1,026 |
| Sichuan | 75,519 | 83,210 | 908 |
| Beijing | 16,964 | 21,920 | 774 |
| Xinjiang | 17,685 | 25,200 | 698 |
| Liaoning | 26,875 | 42,910 | 626 |
| Shanxi | 14,900 | 35,020 | 426 |
| Qinghai | - | 5,870 | - |
| Tibet | - | 3,540 | - |

*Provinces are sorted by per capita cigarettes production. Data were available from the web site of National Bureau of Statistics [cited 2021 2/12/21]: <https://data.stats.gov.cn/easyquery.htm?cn=E0103>. Data of Qinghai and Tibet were unavailable.
